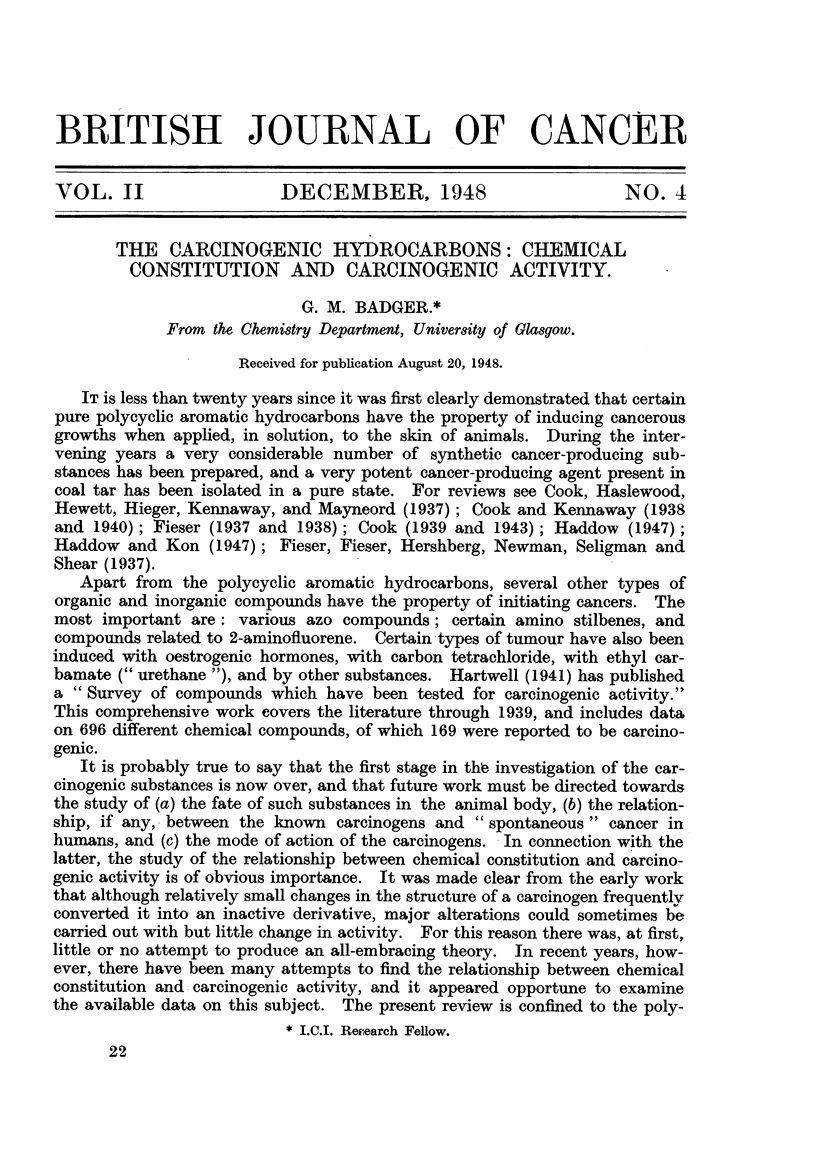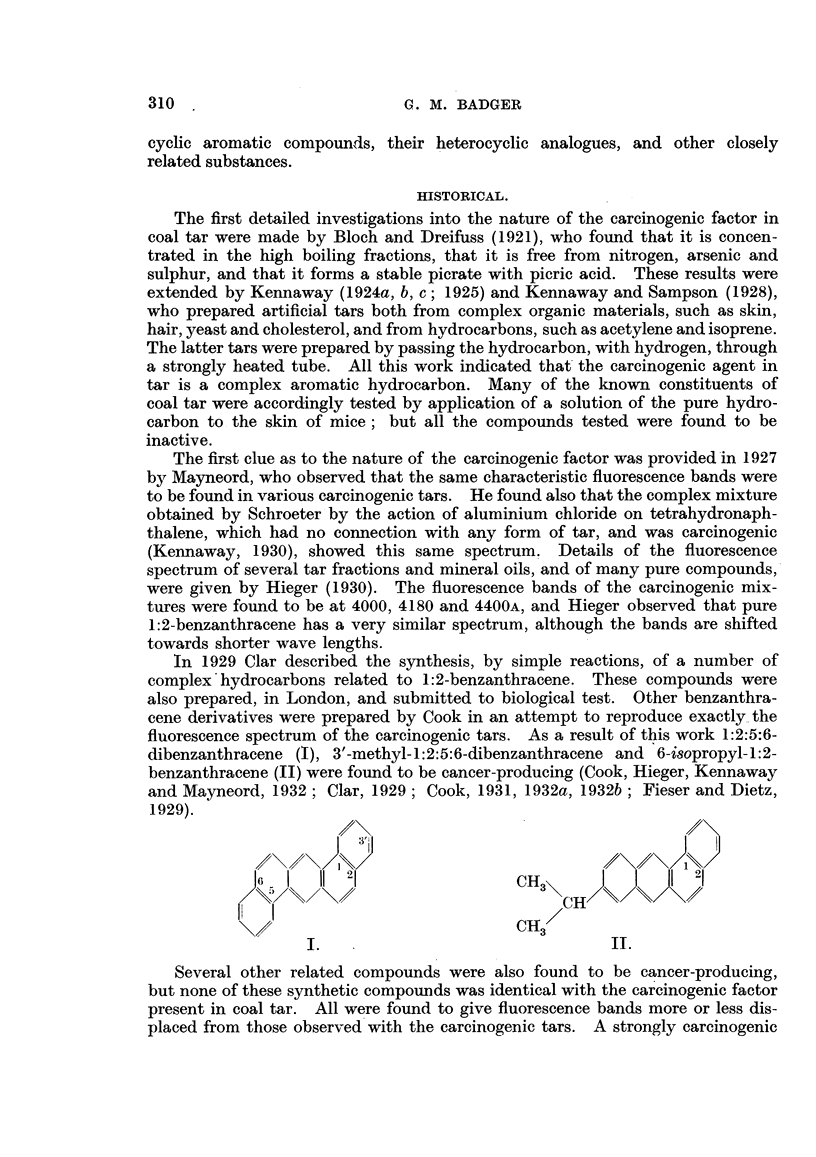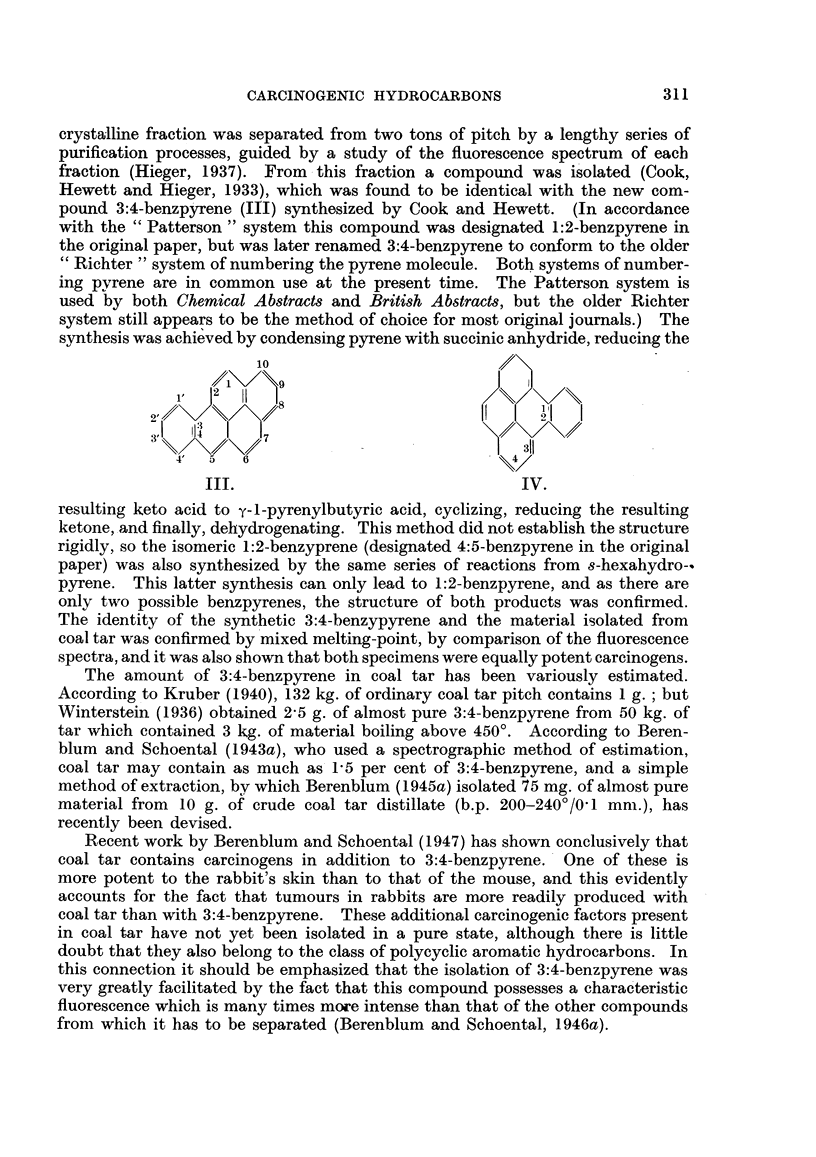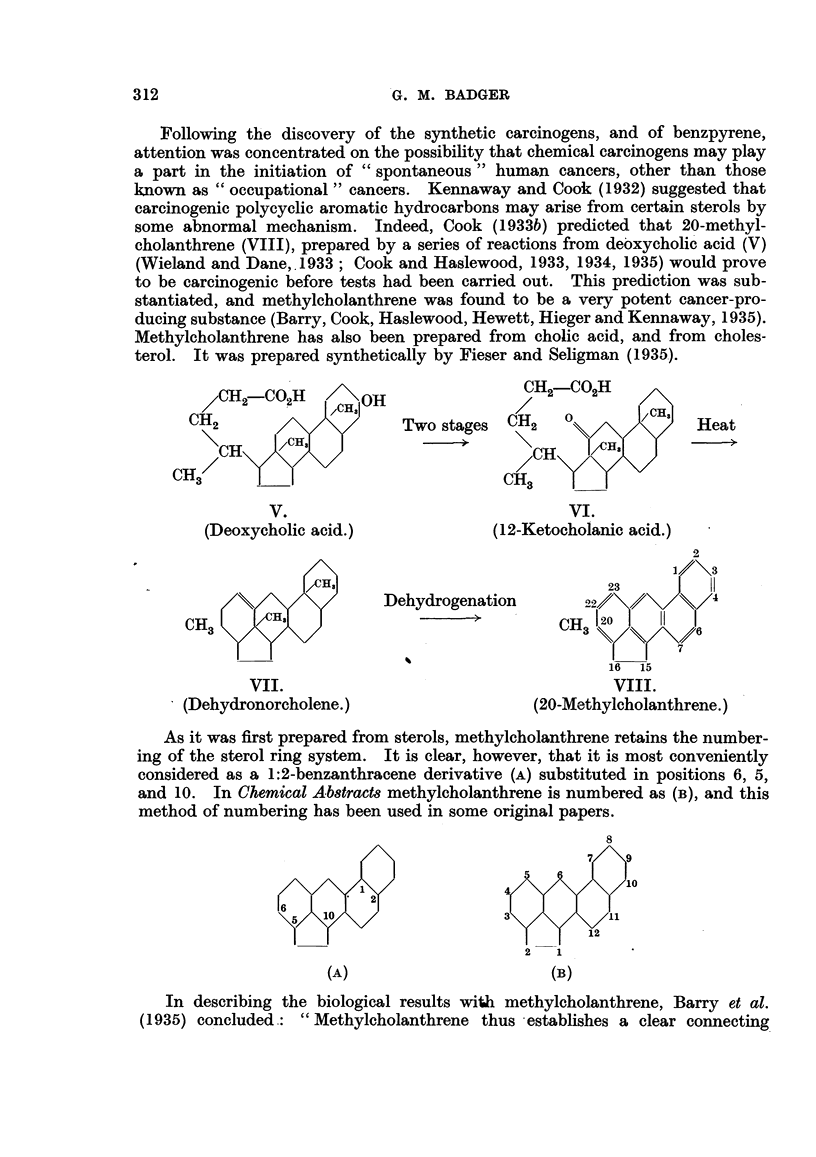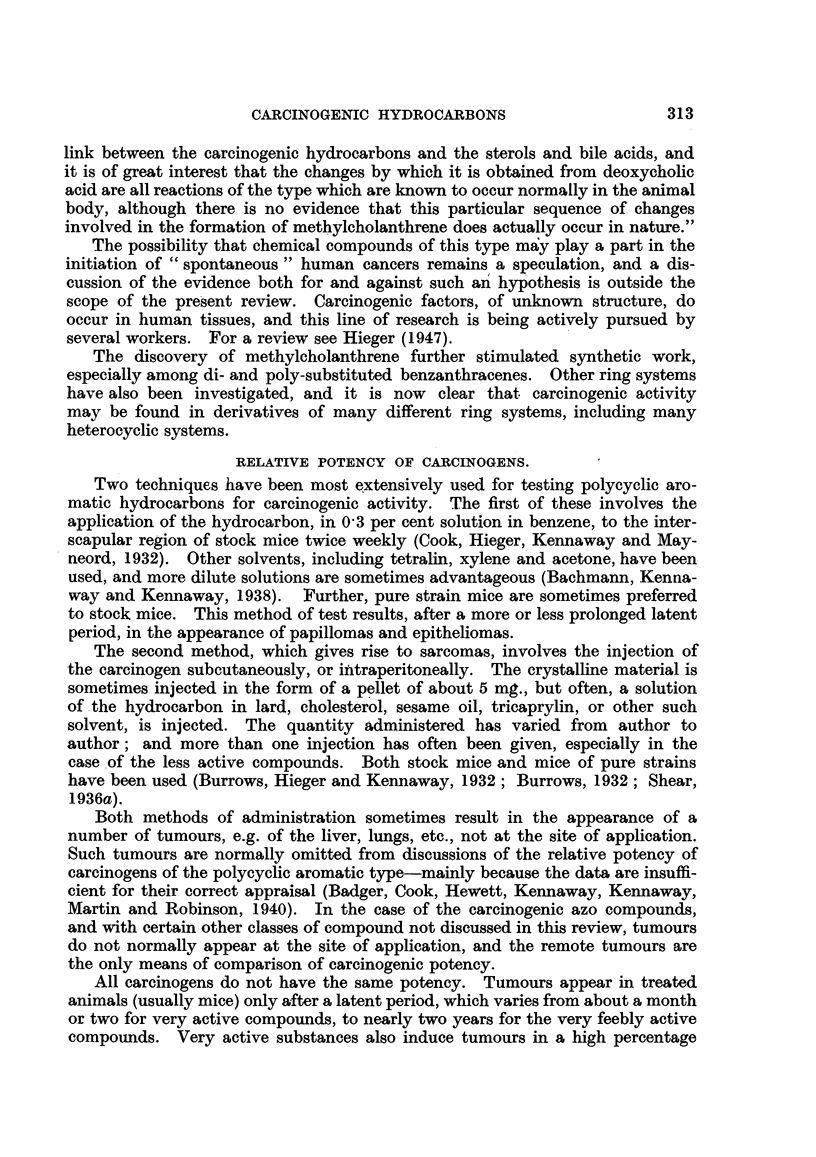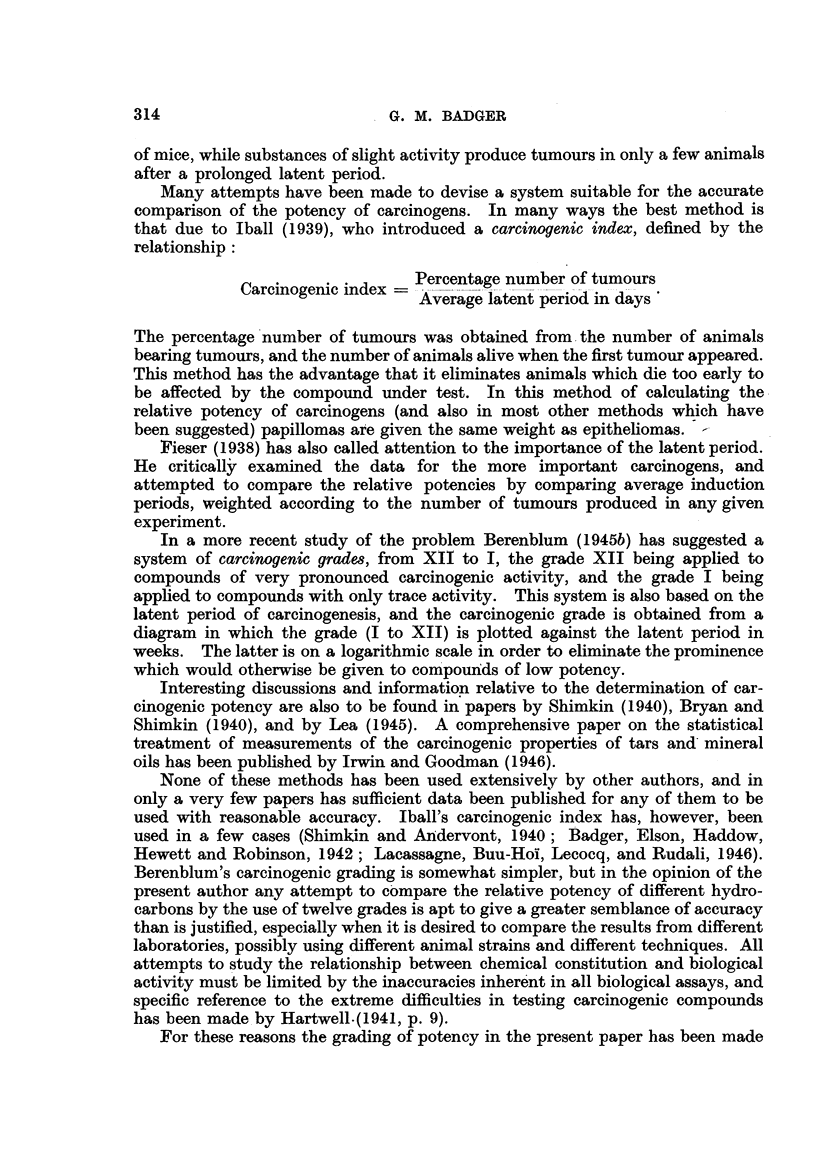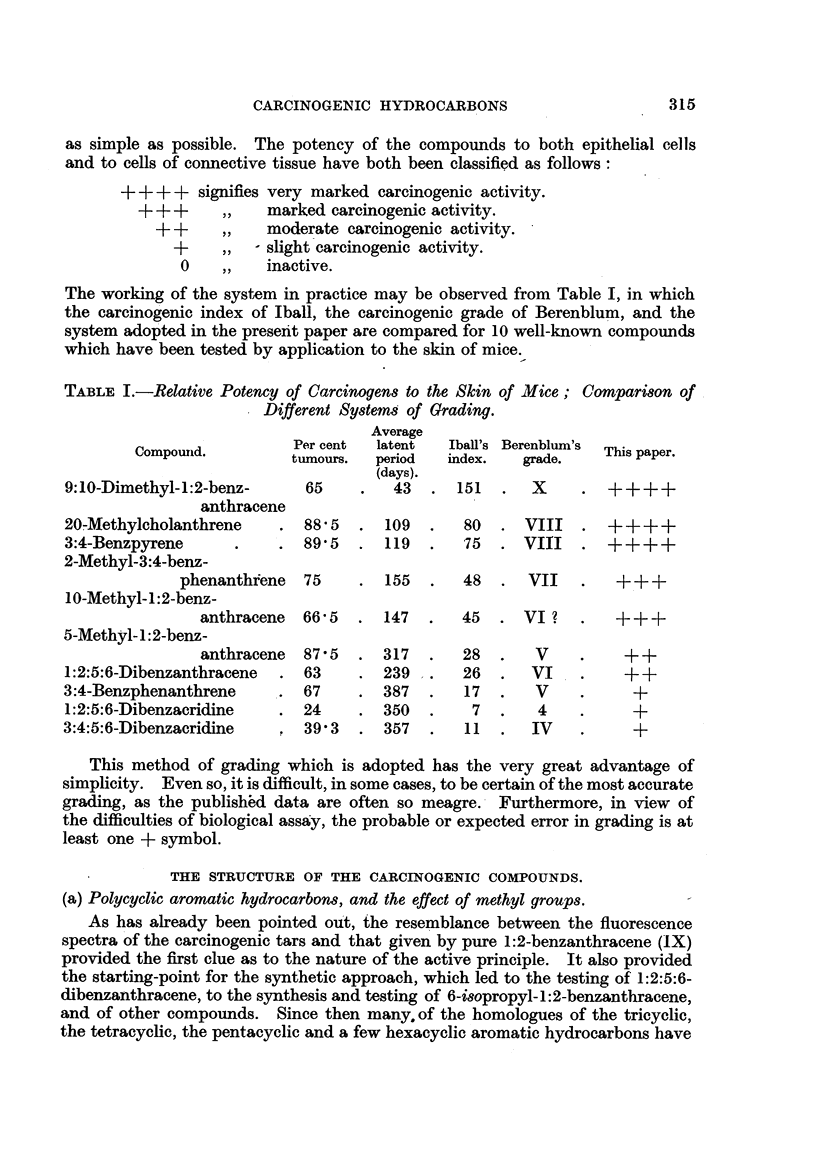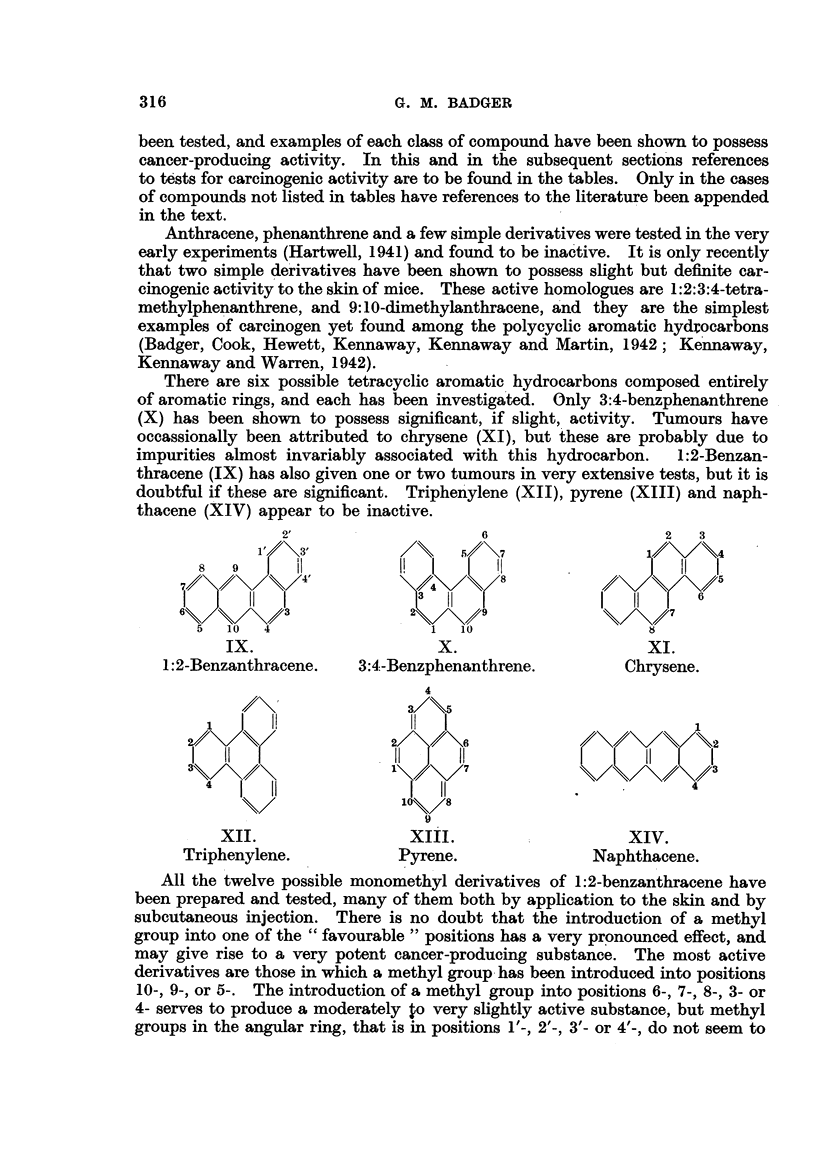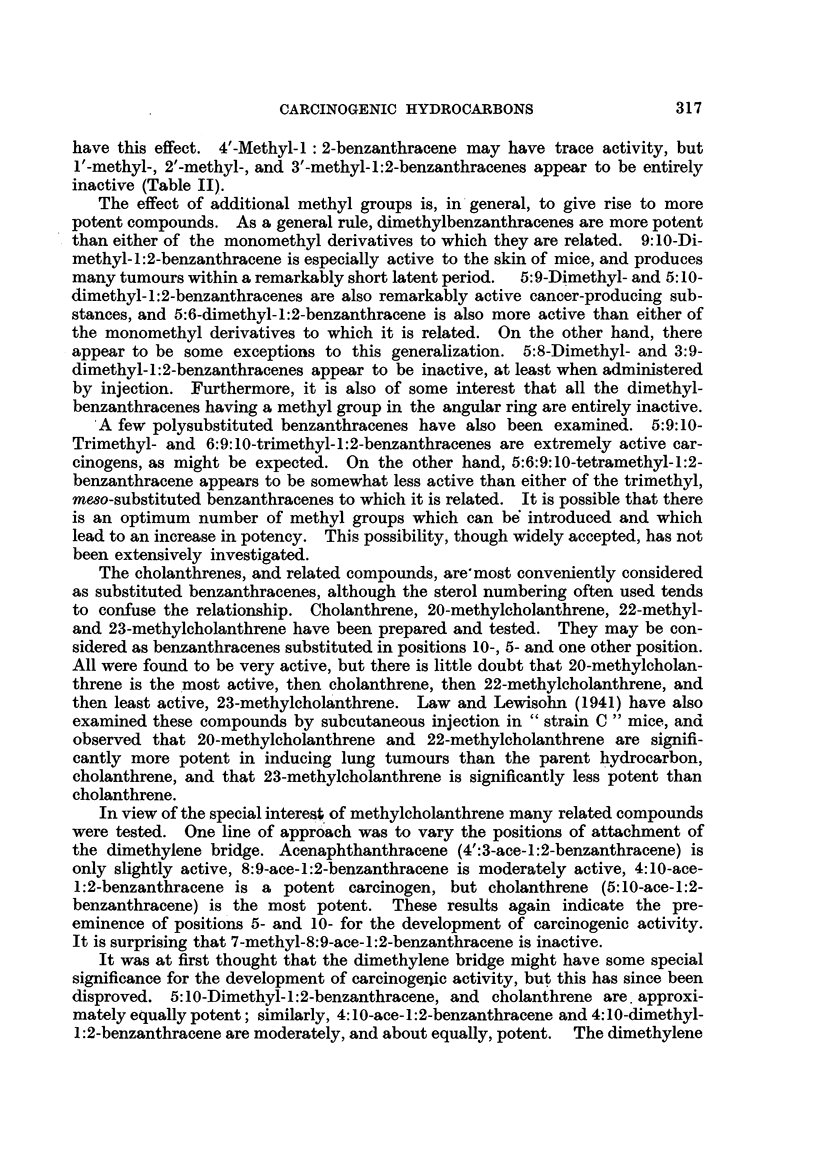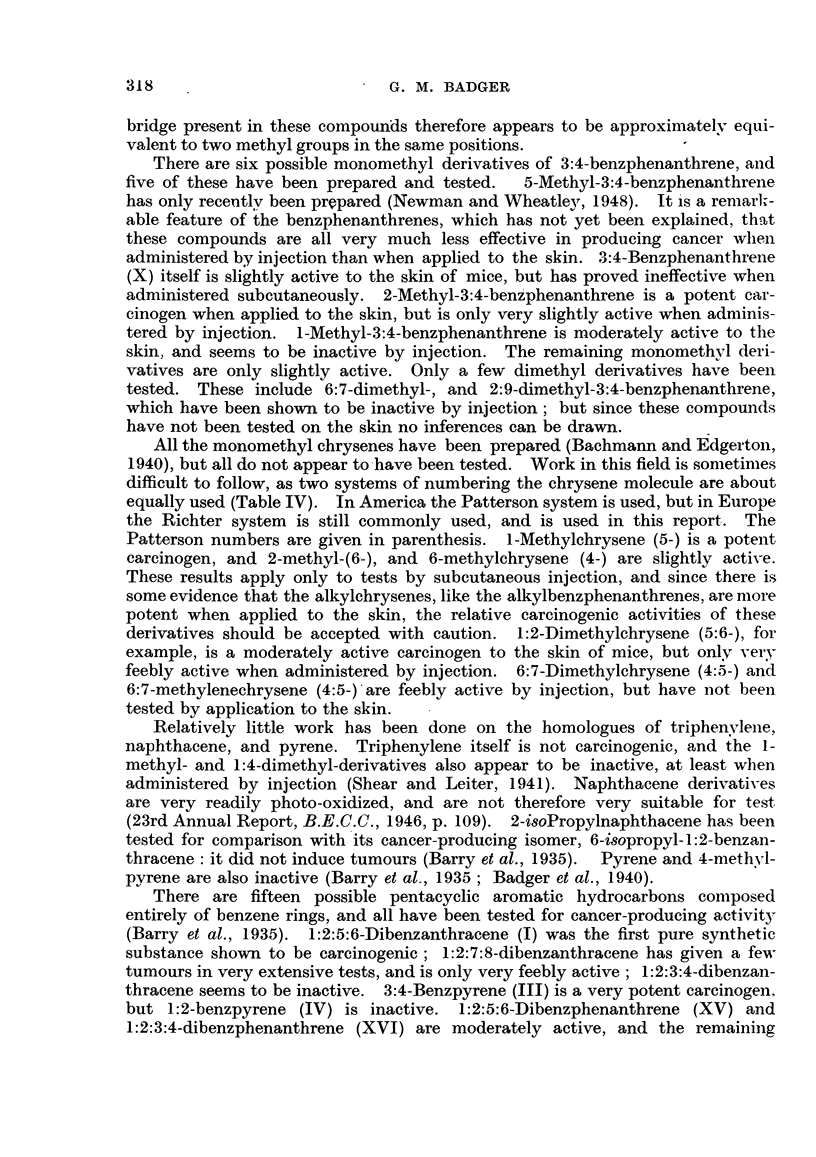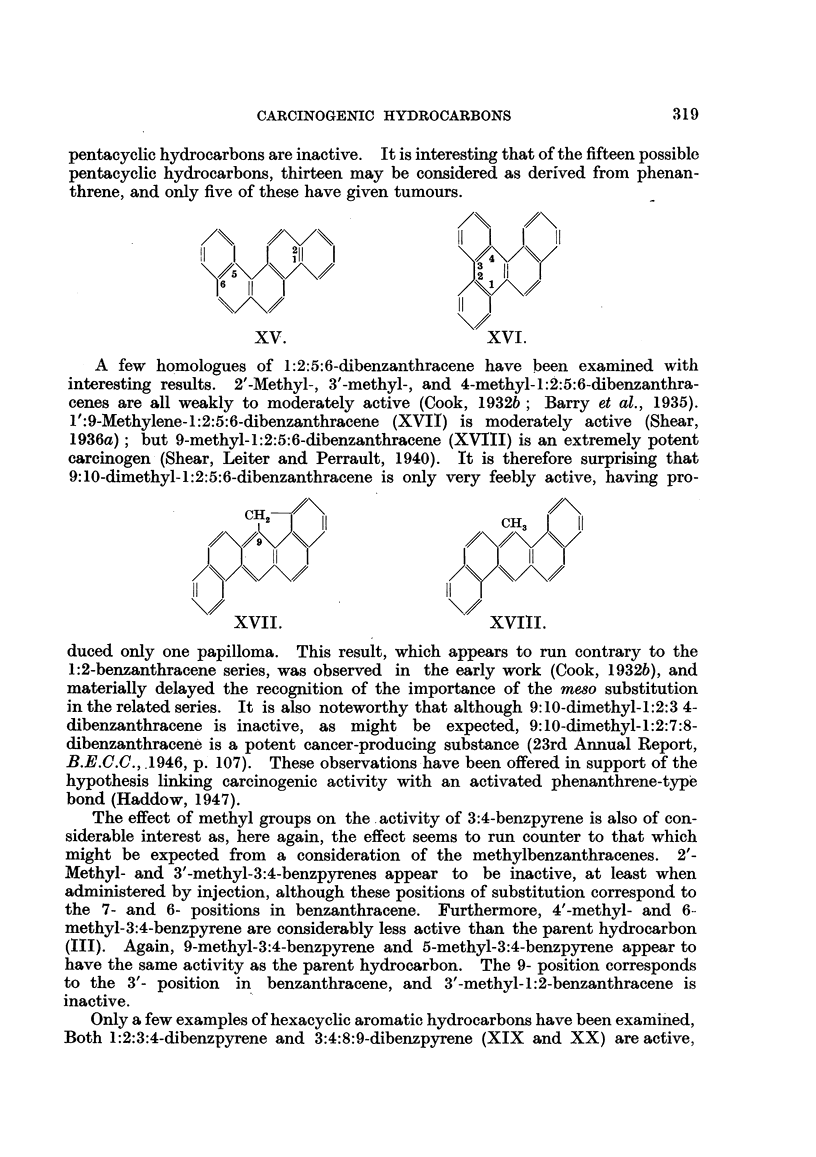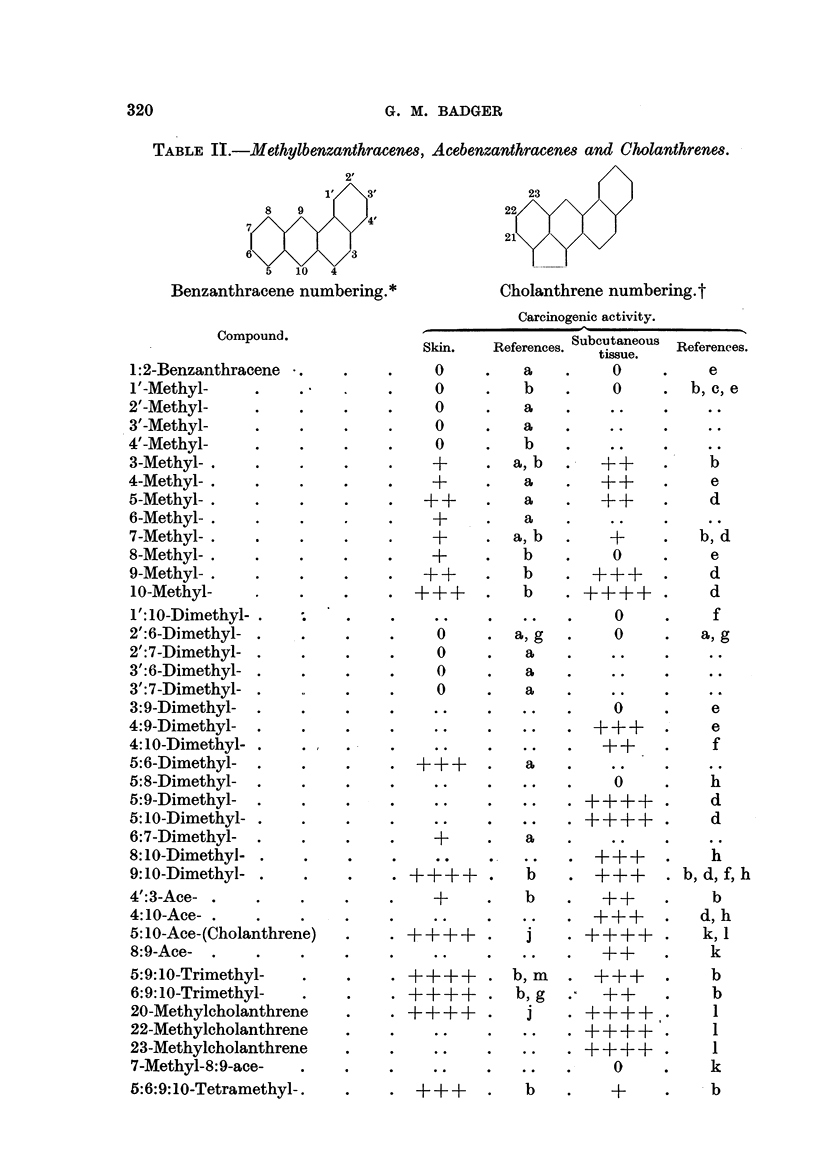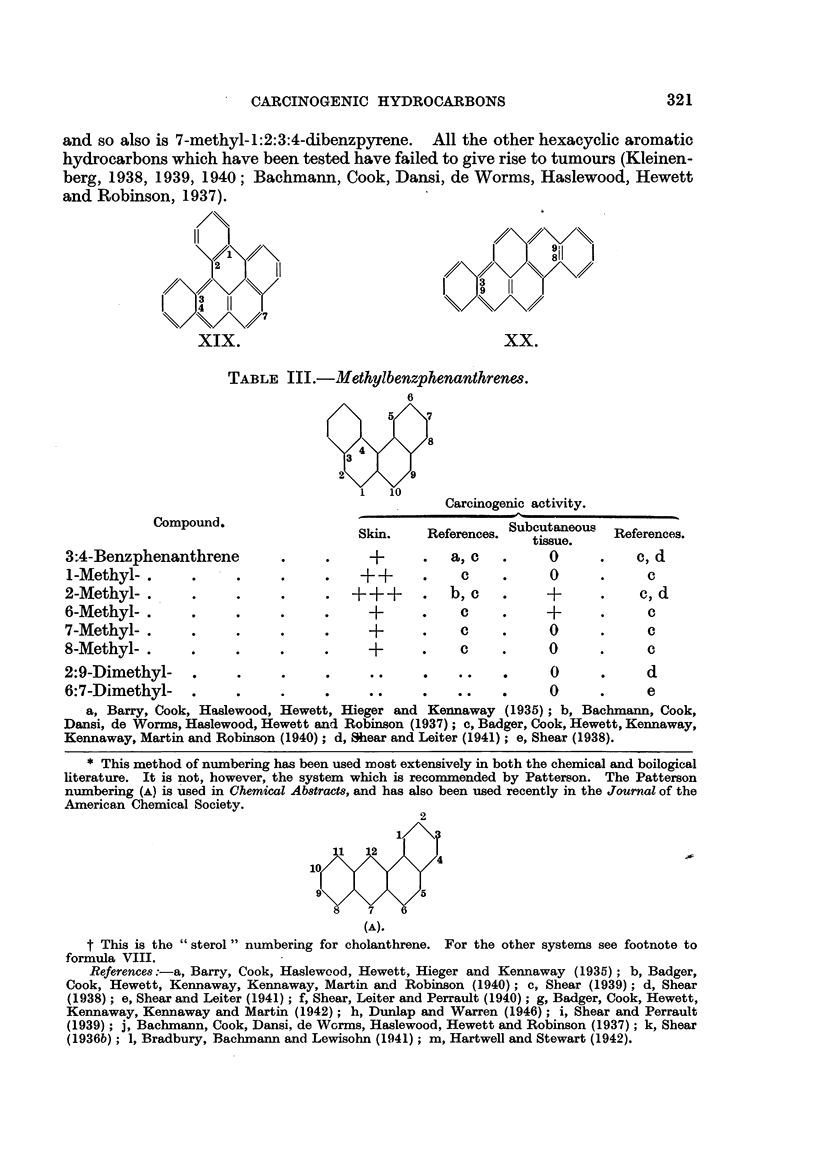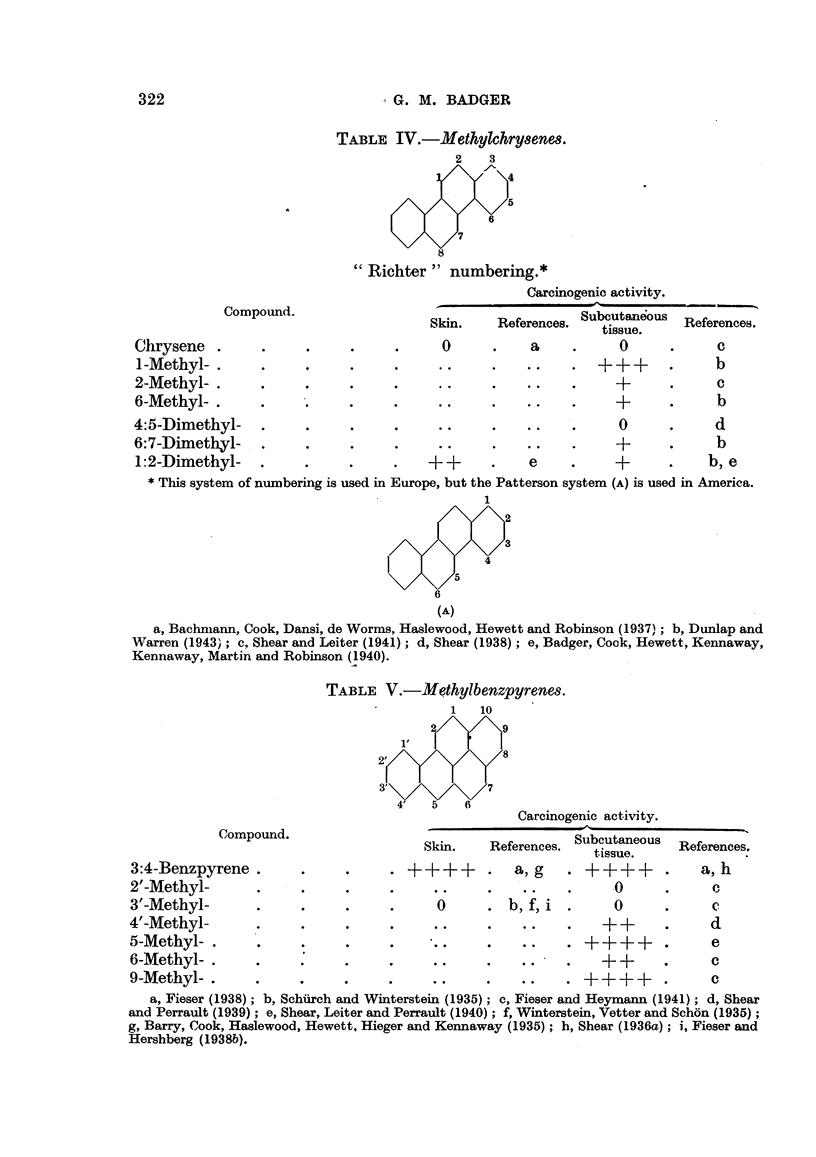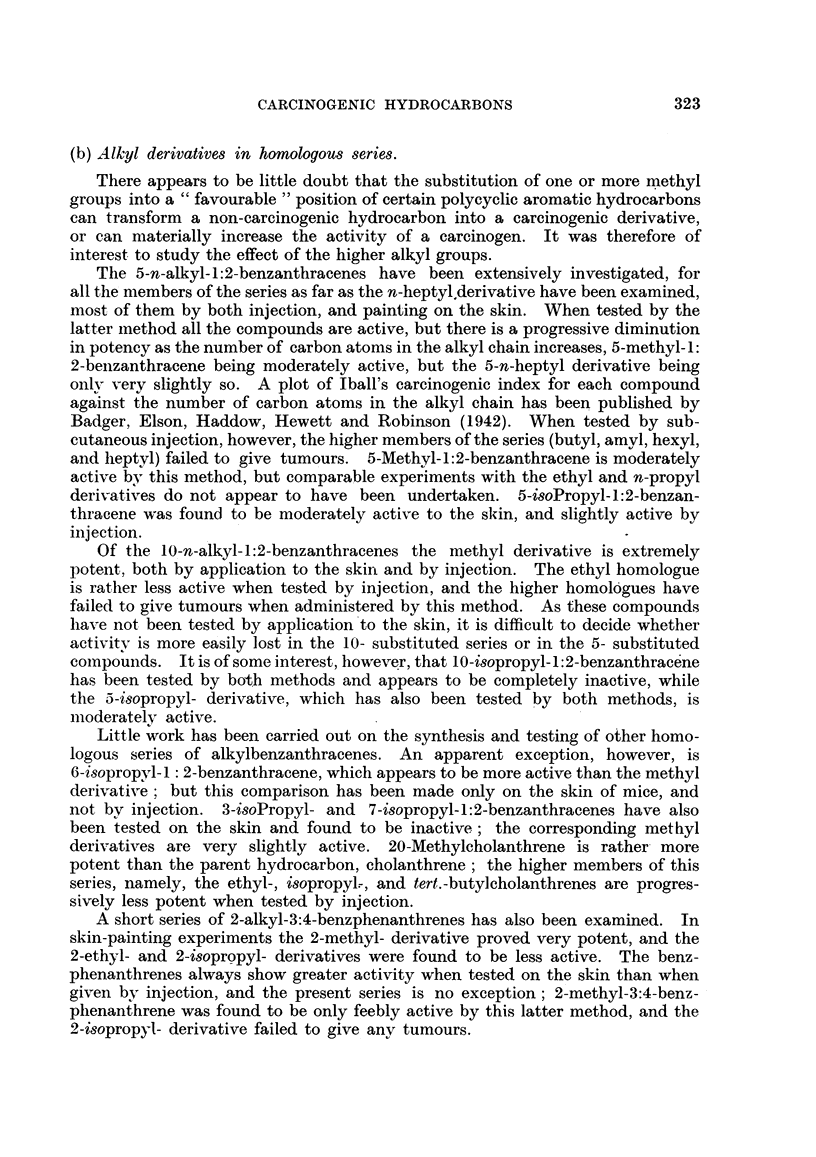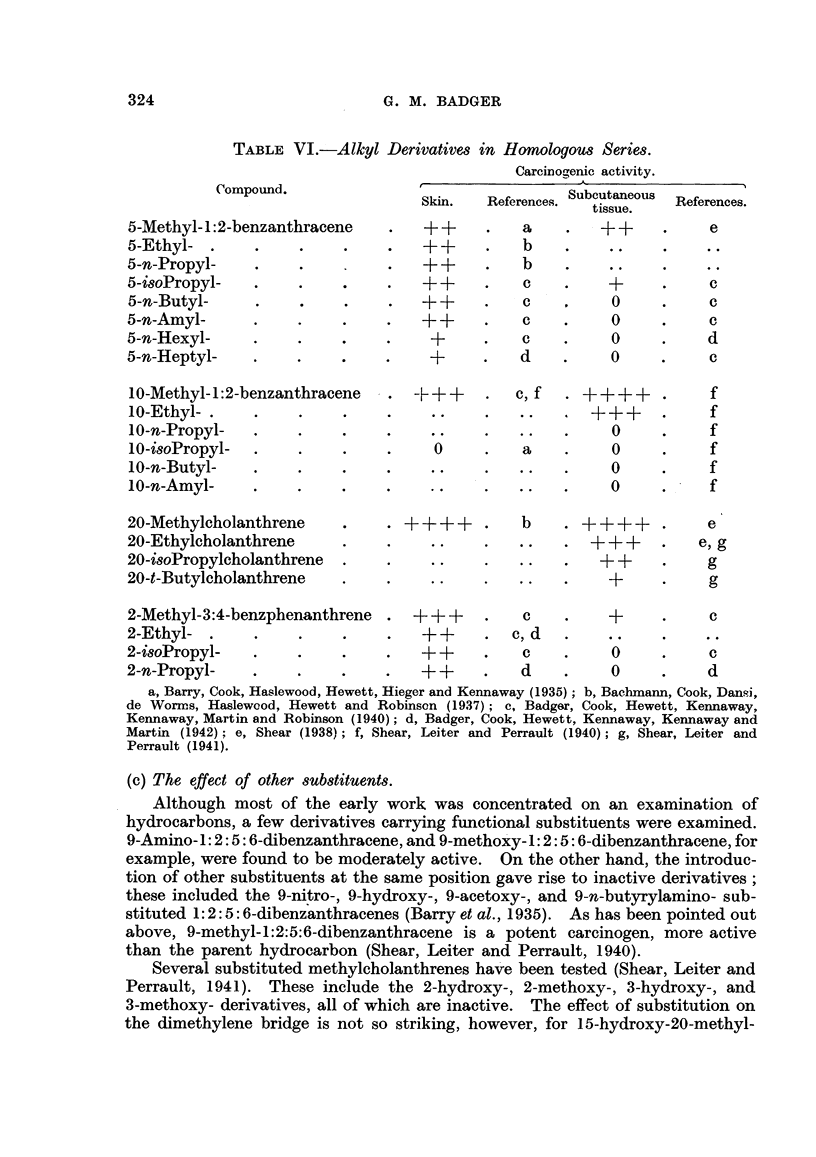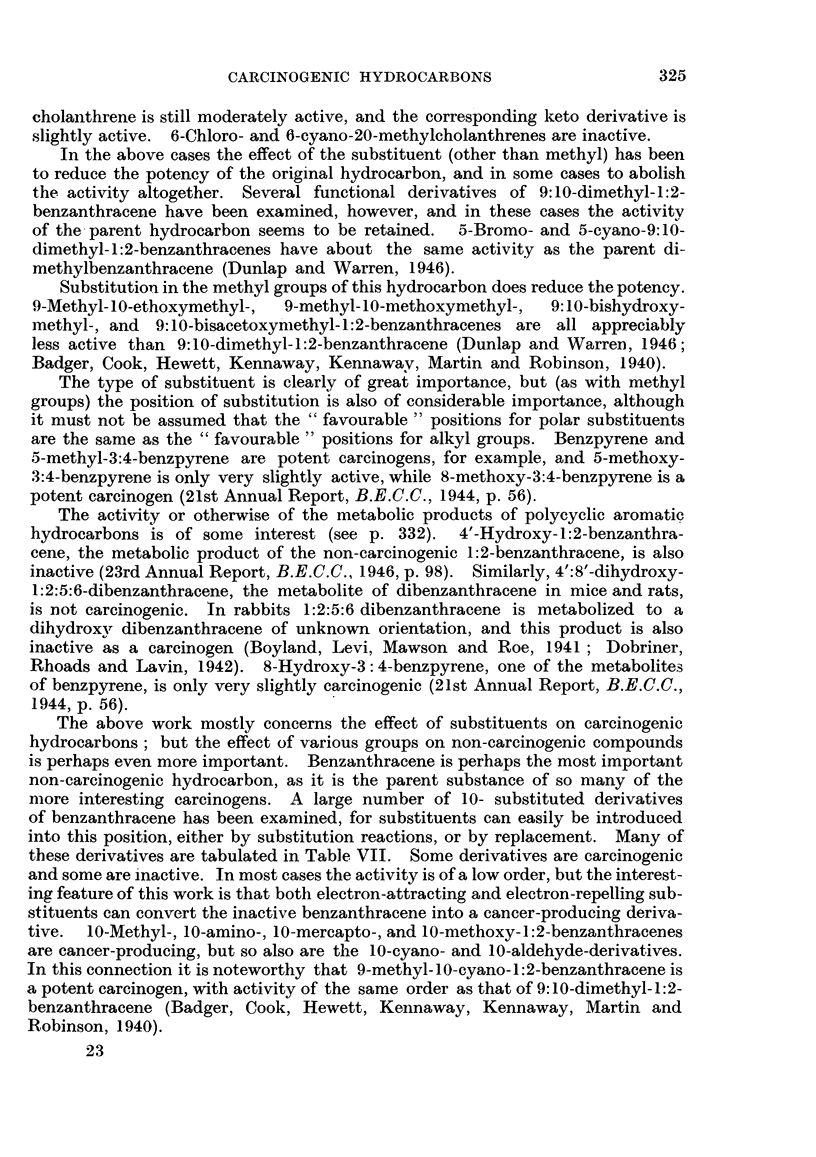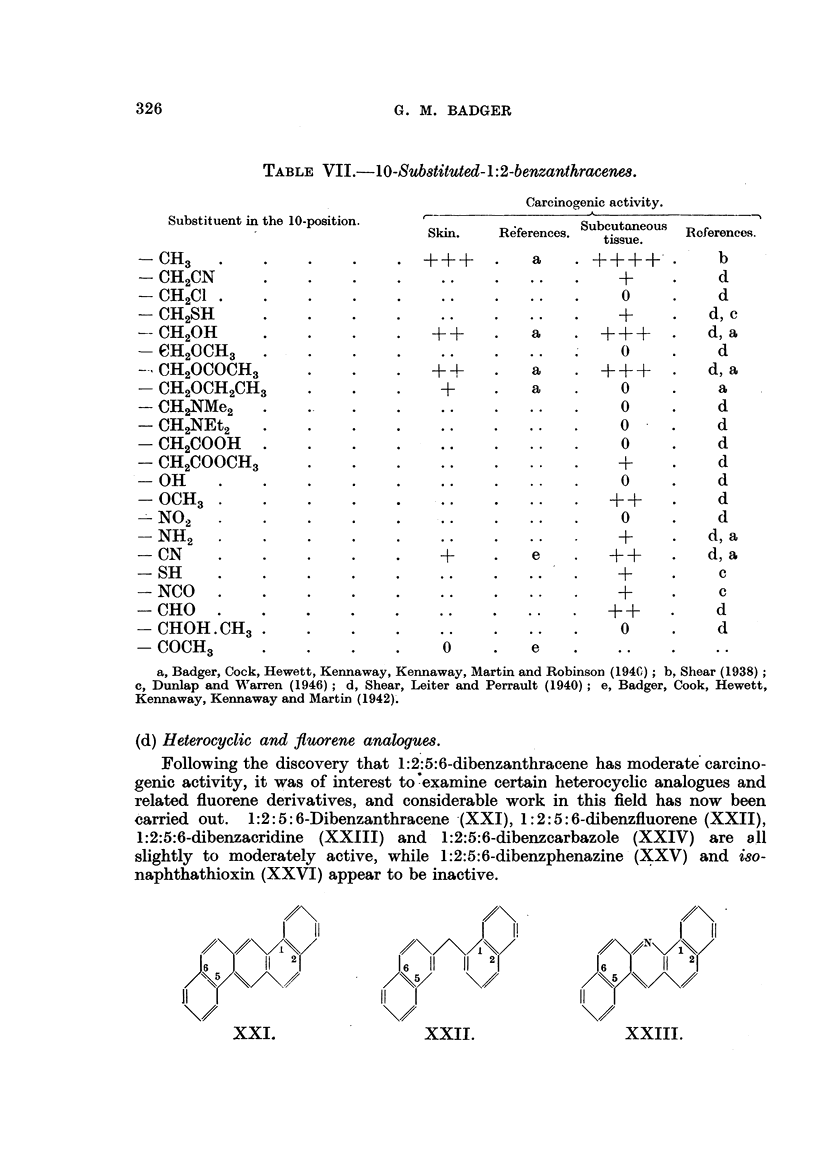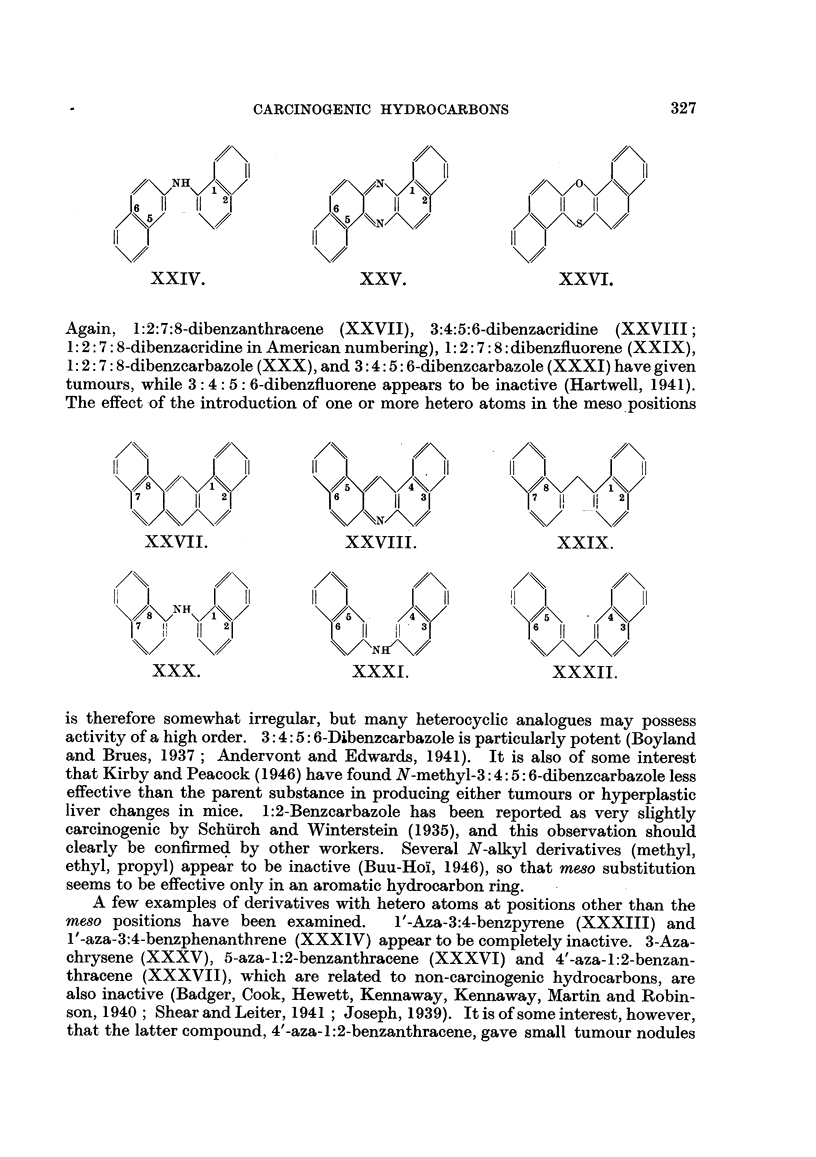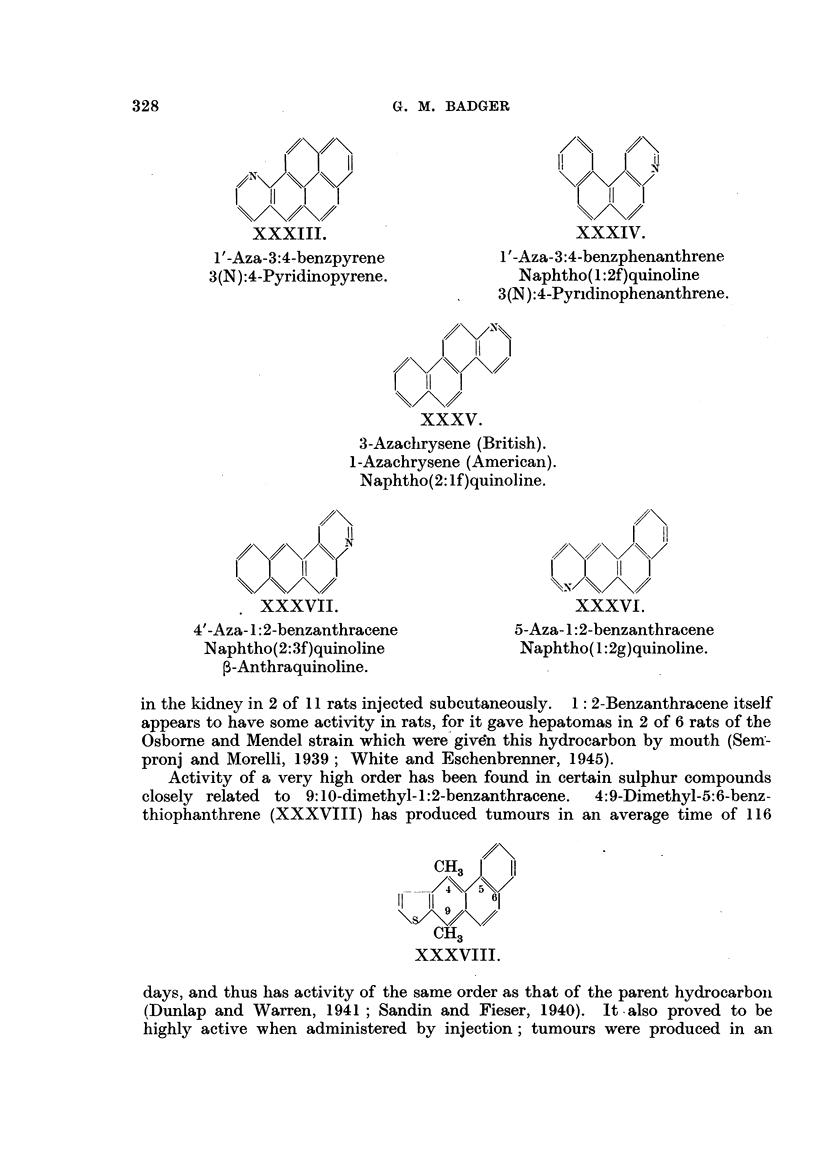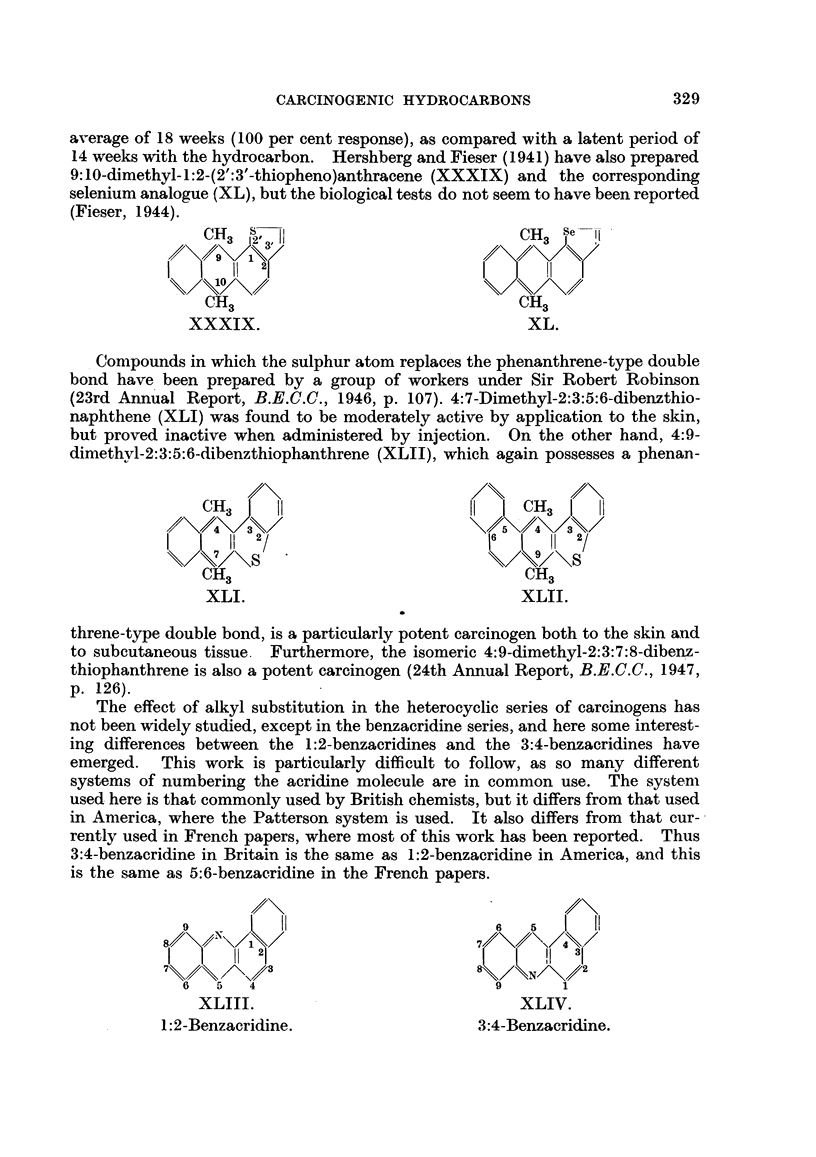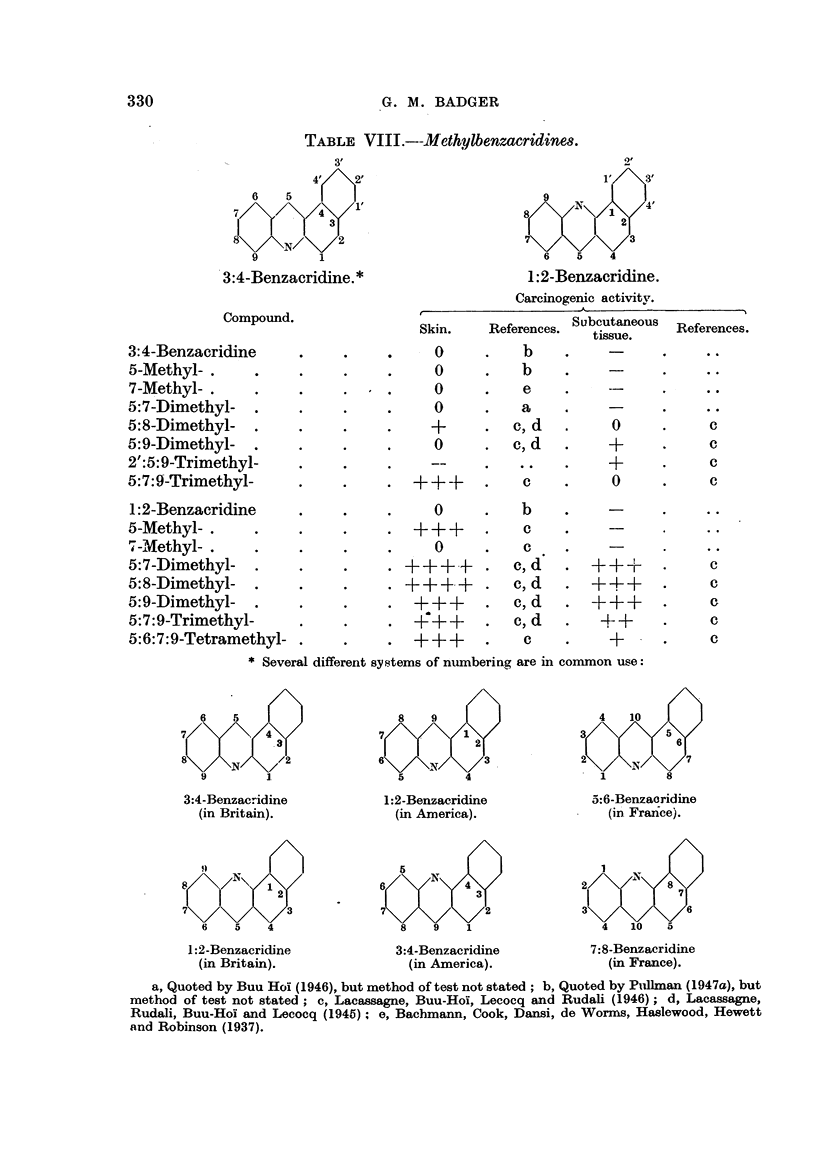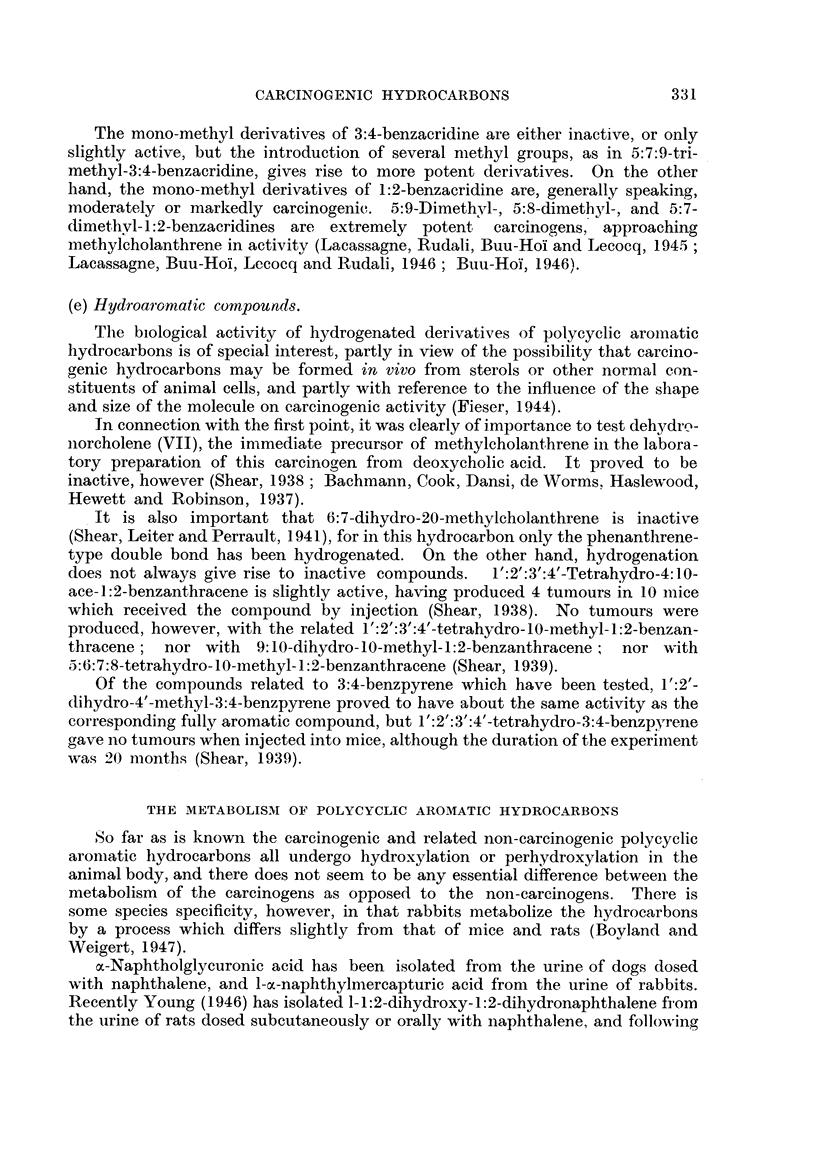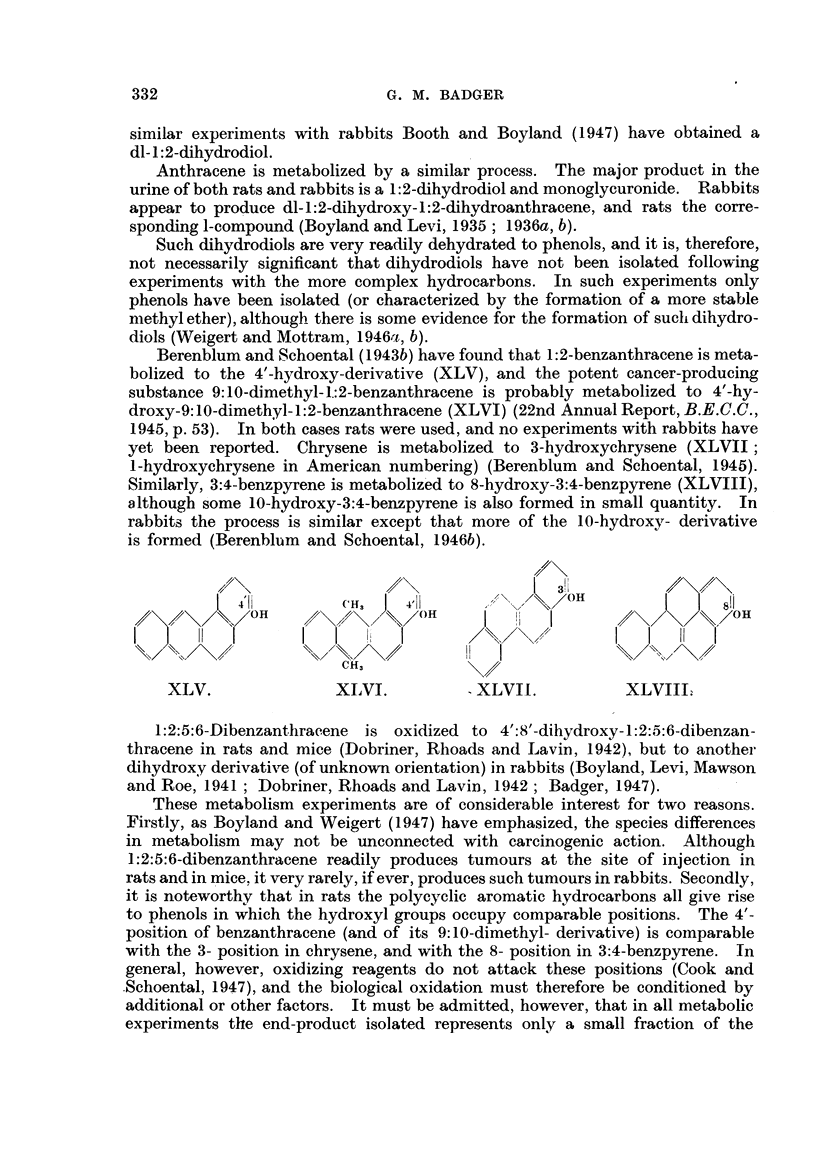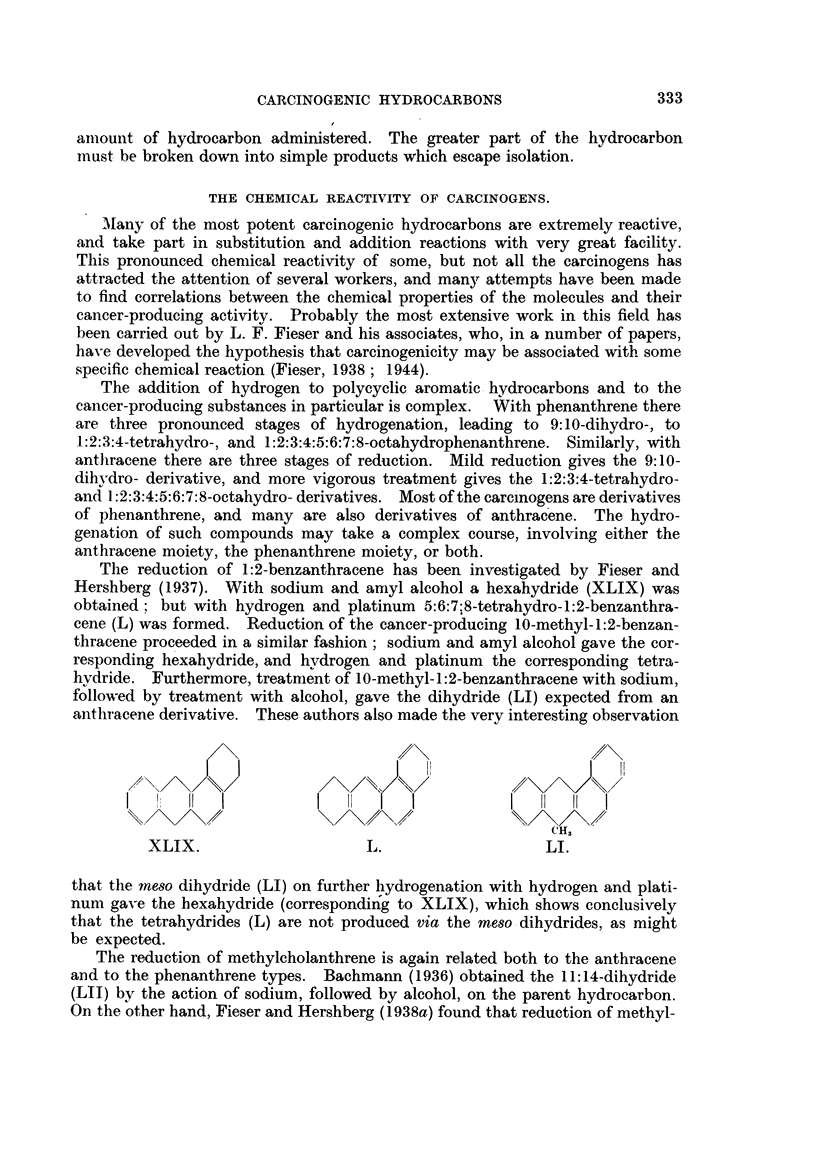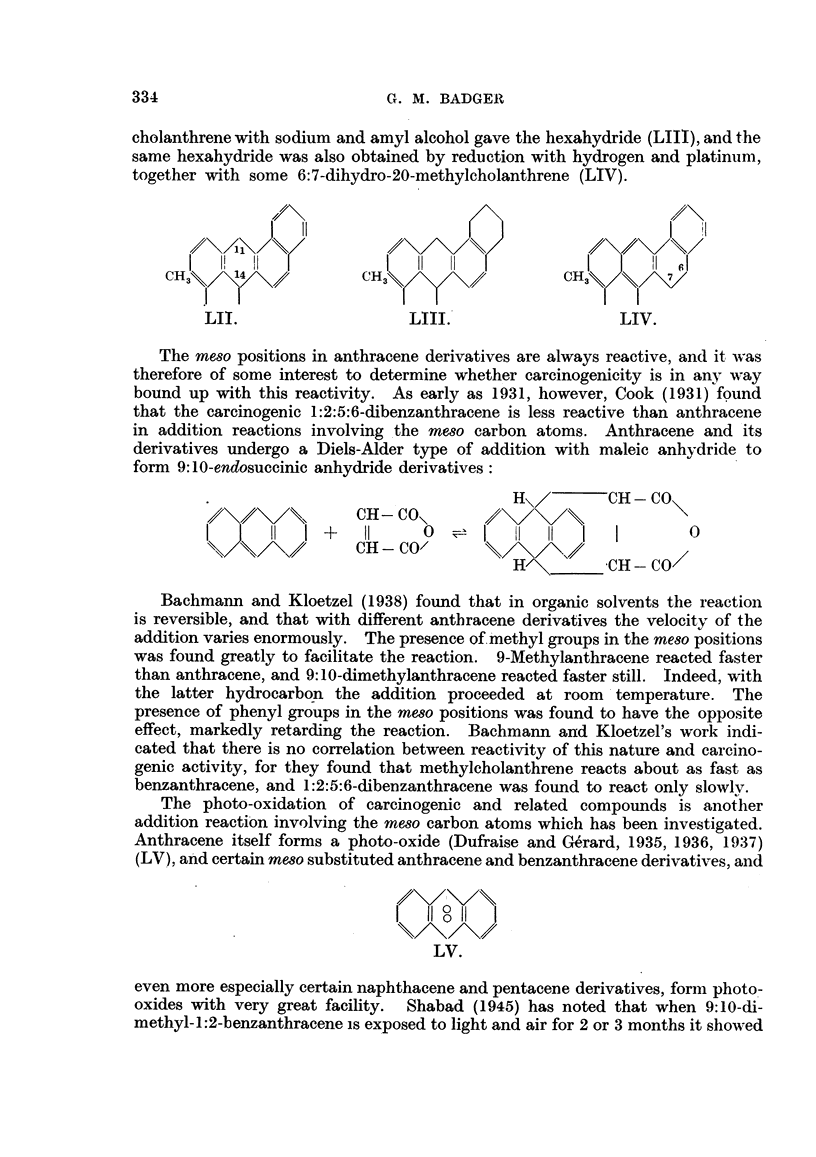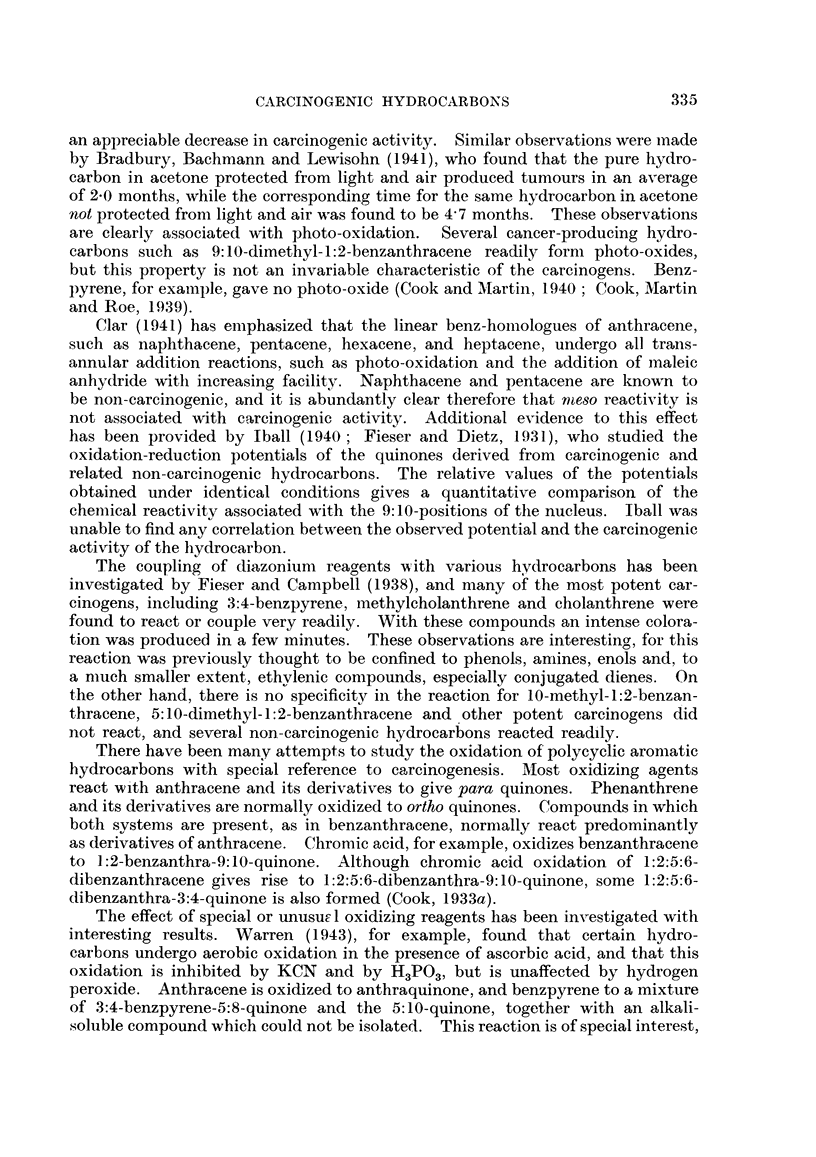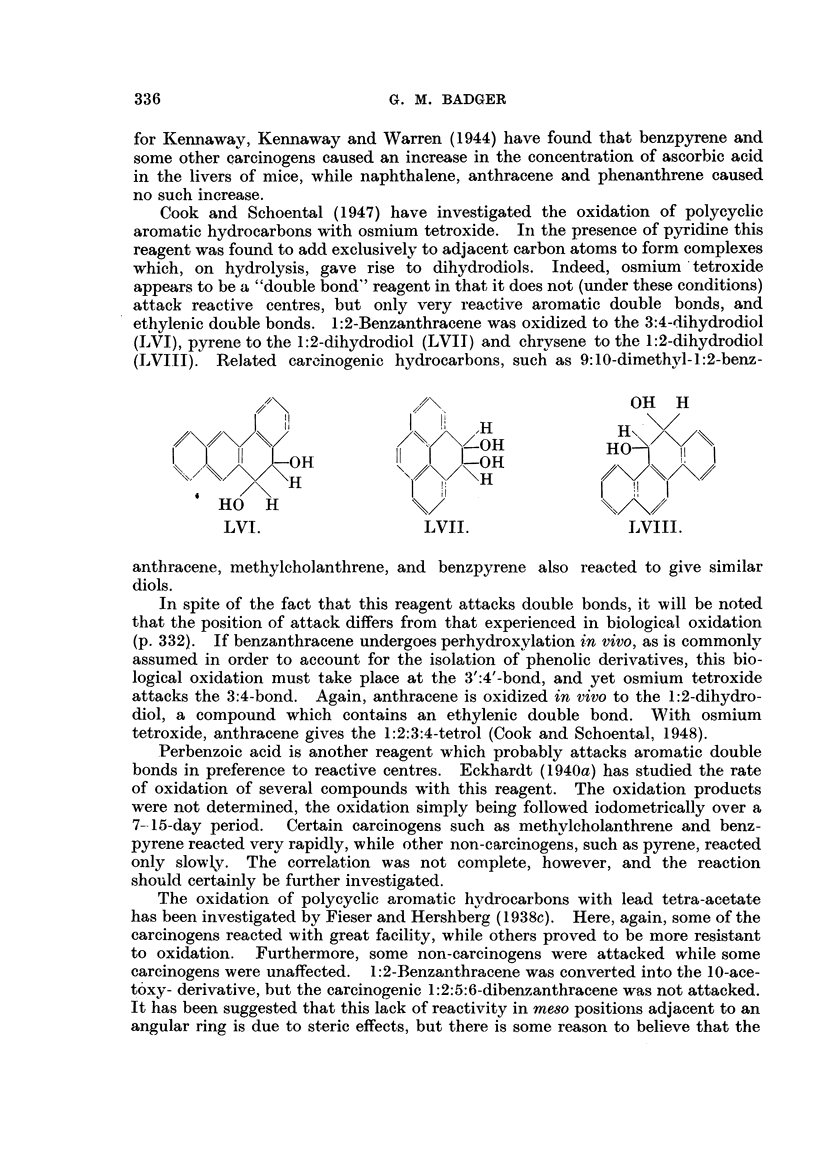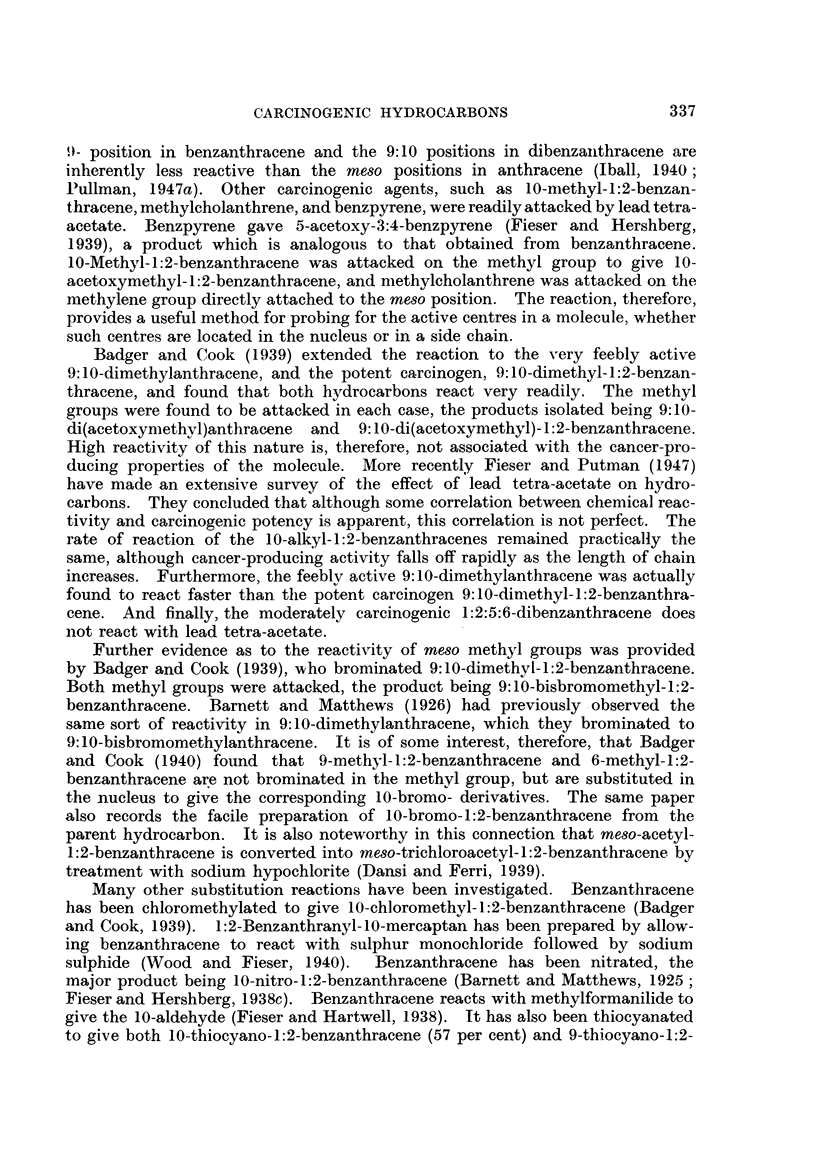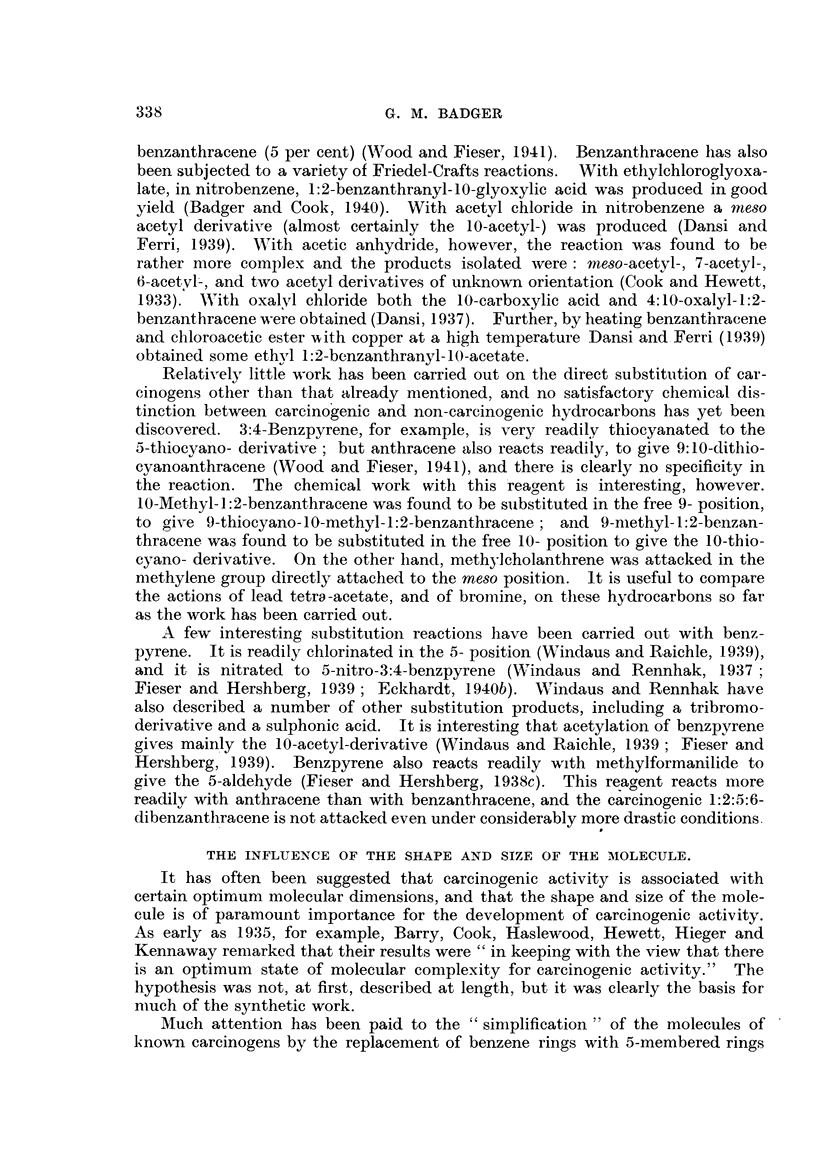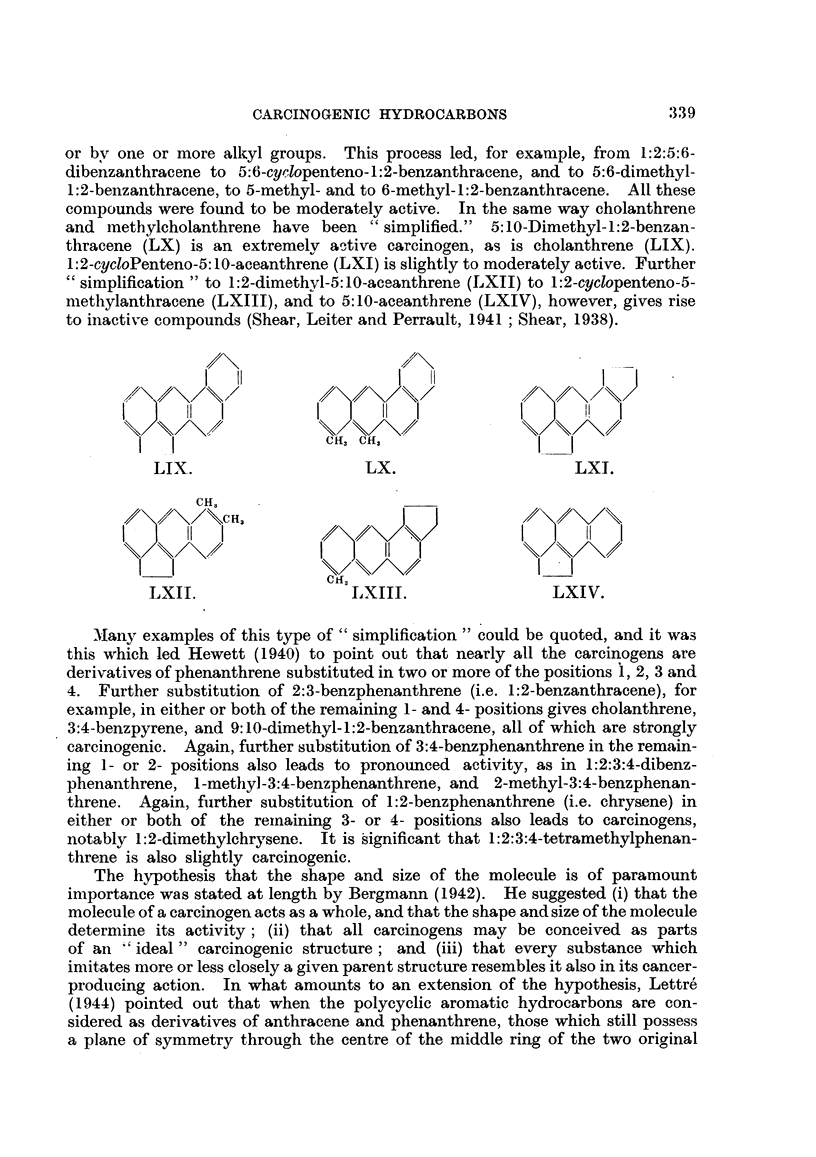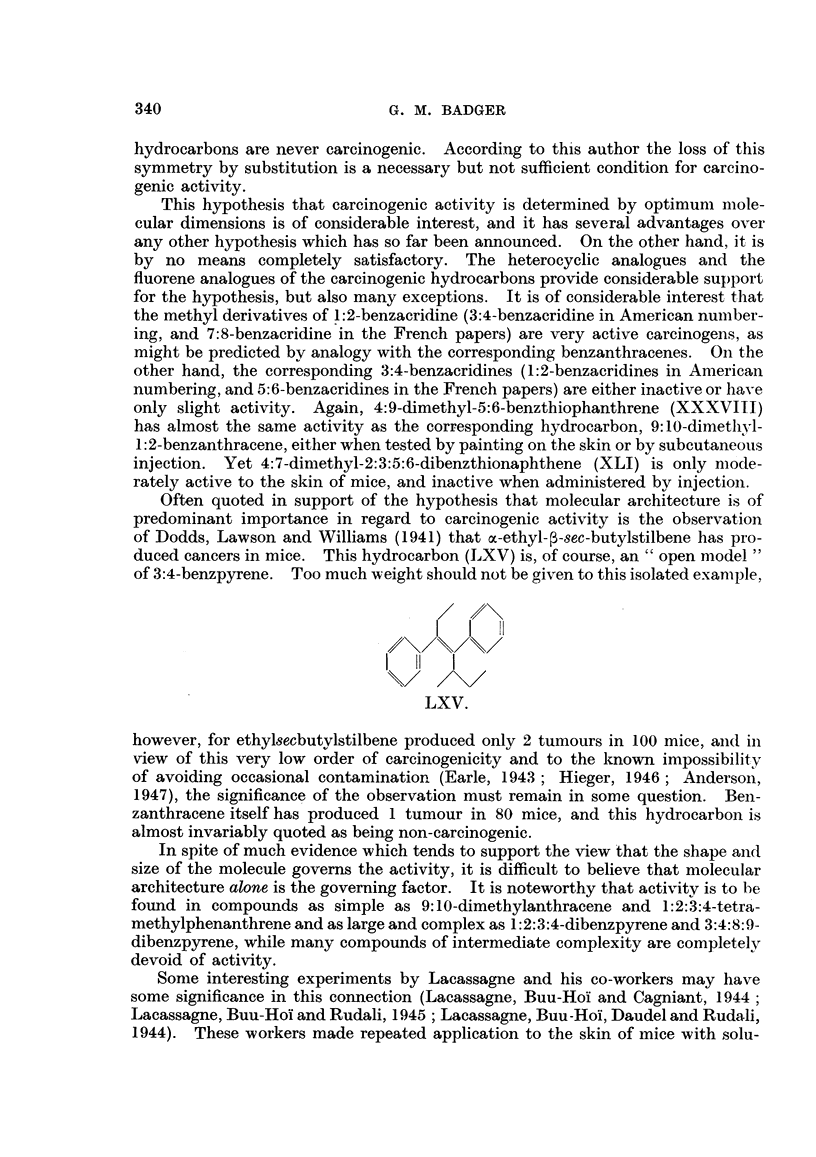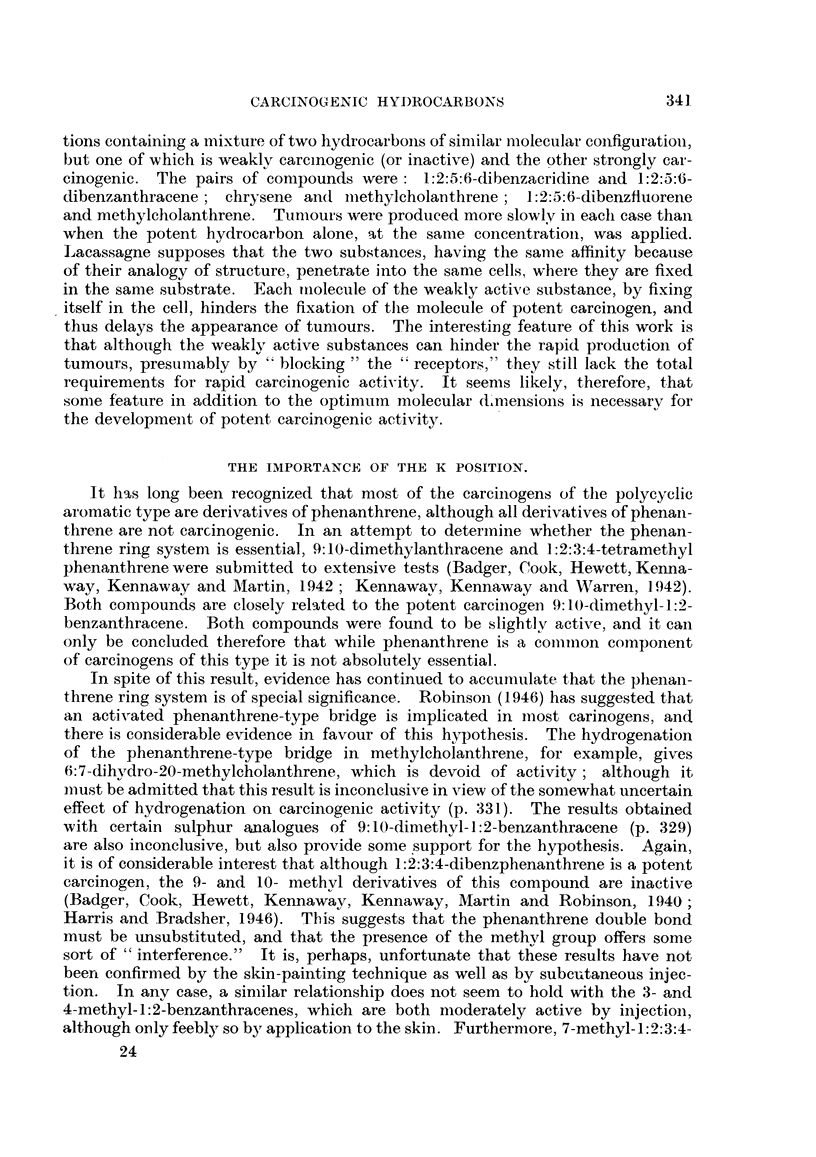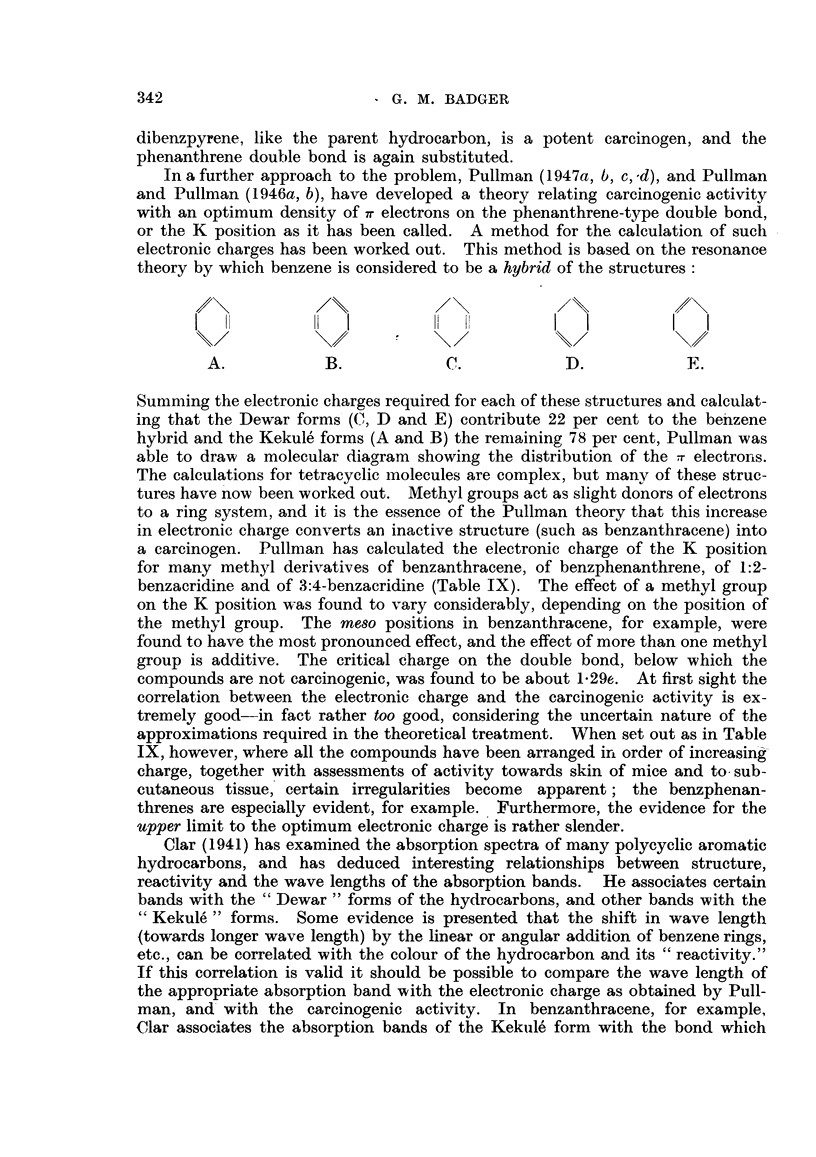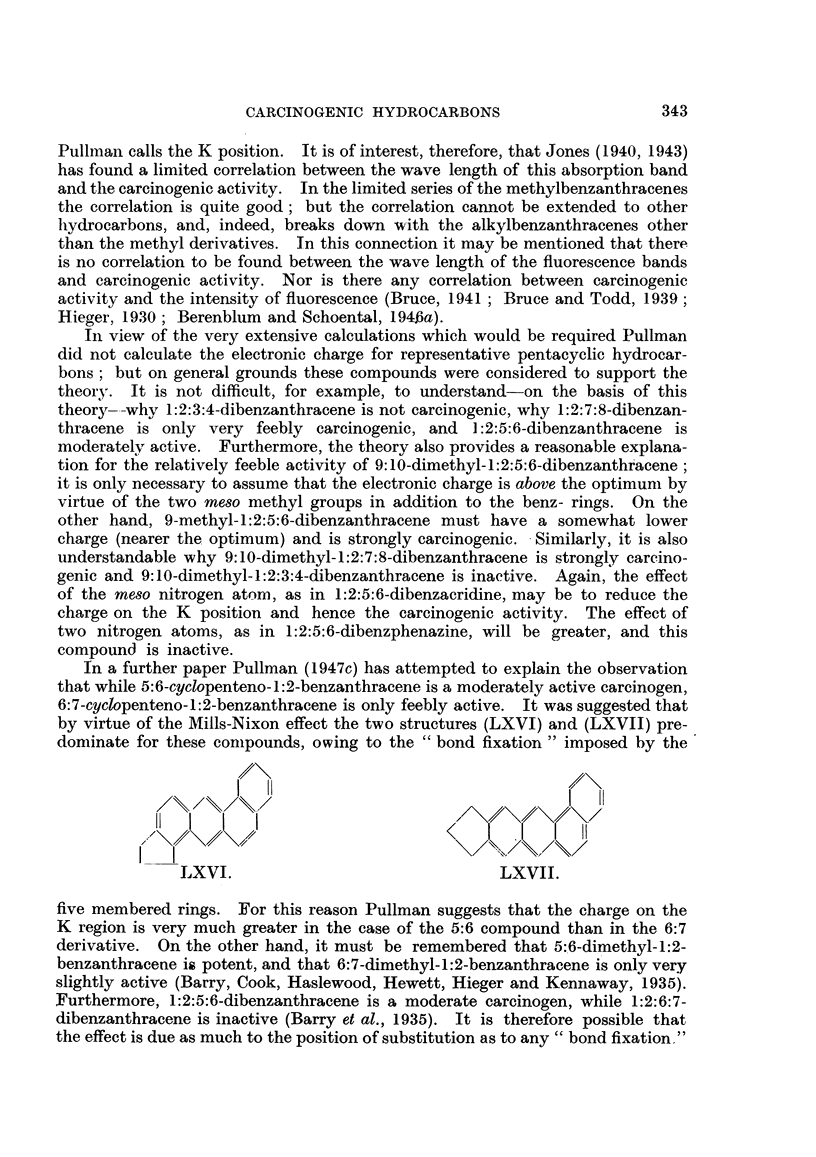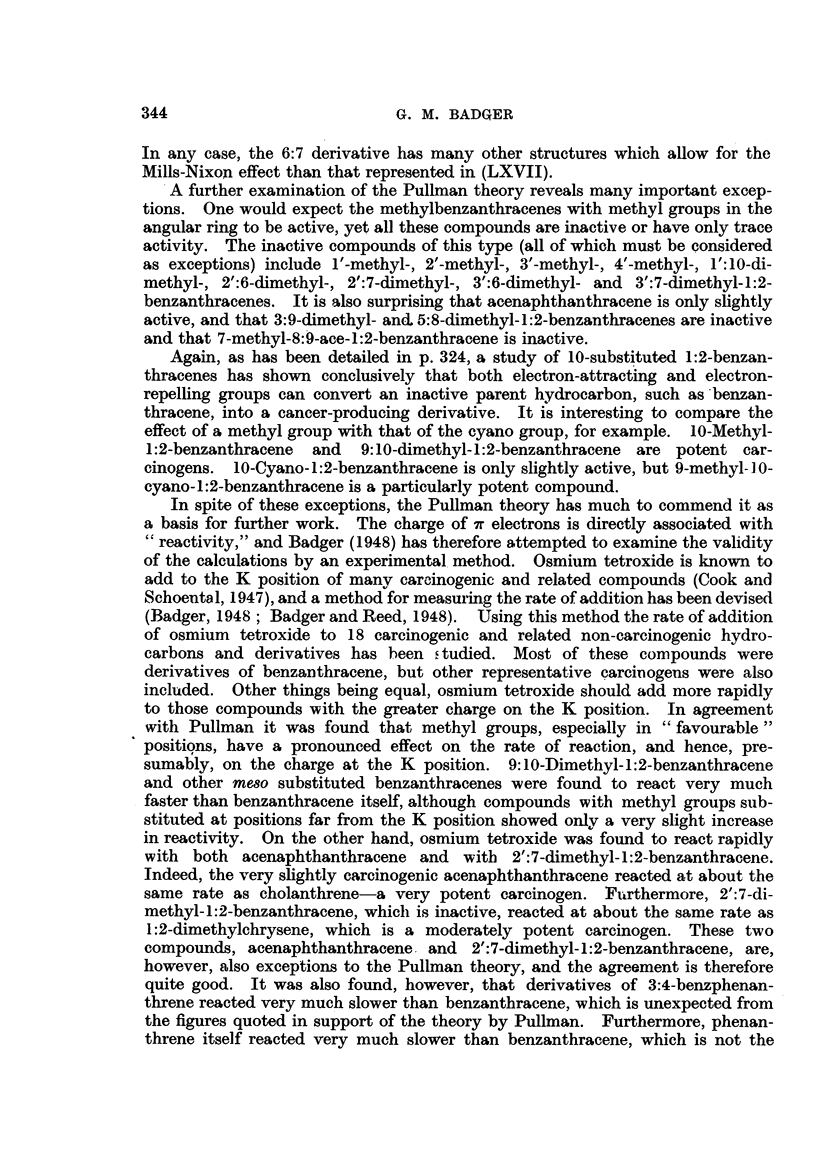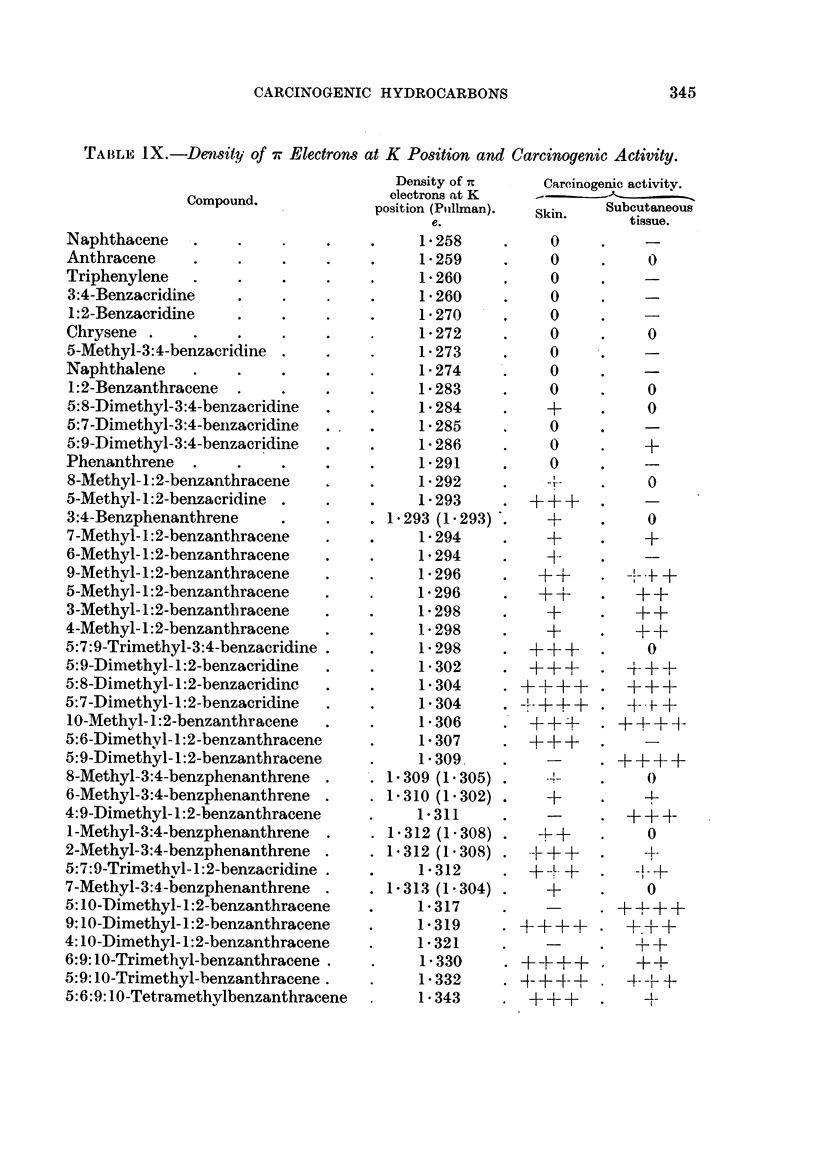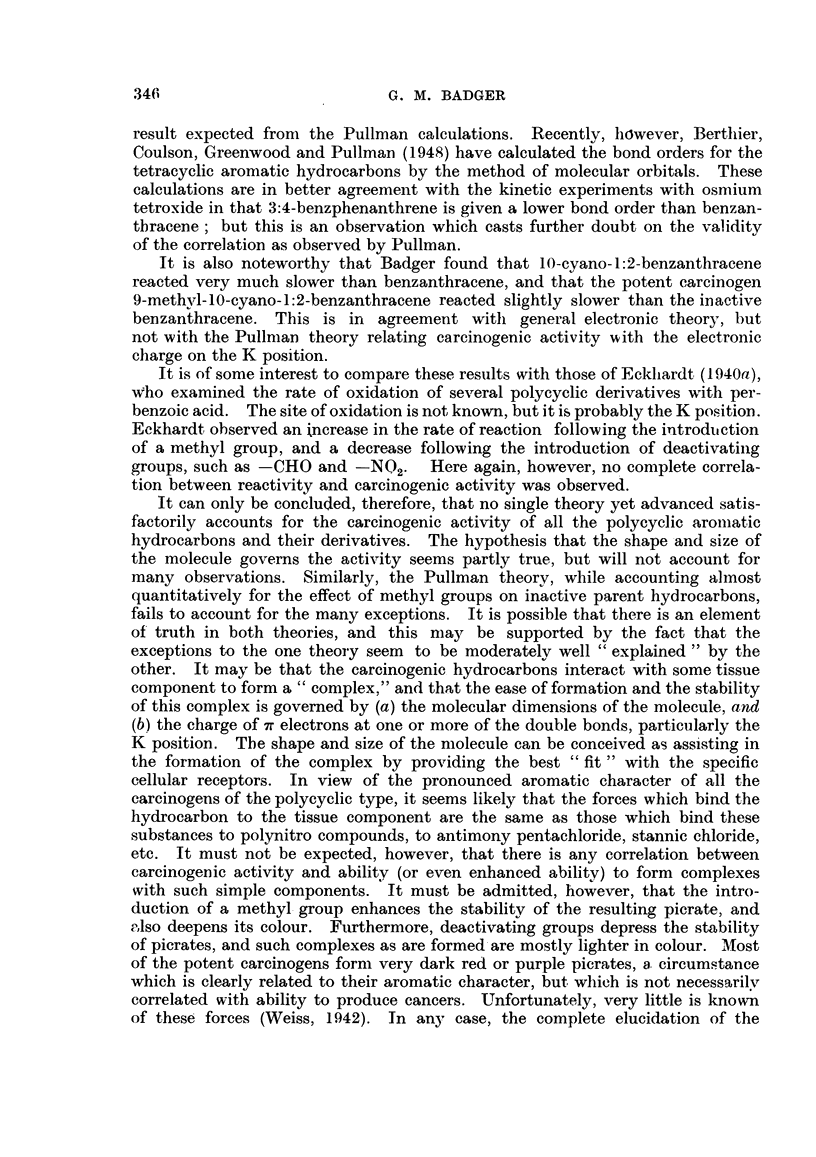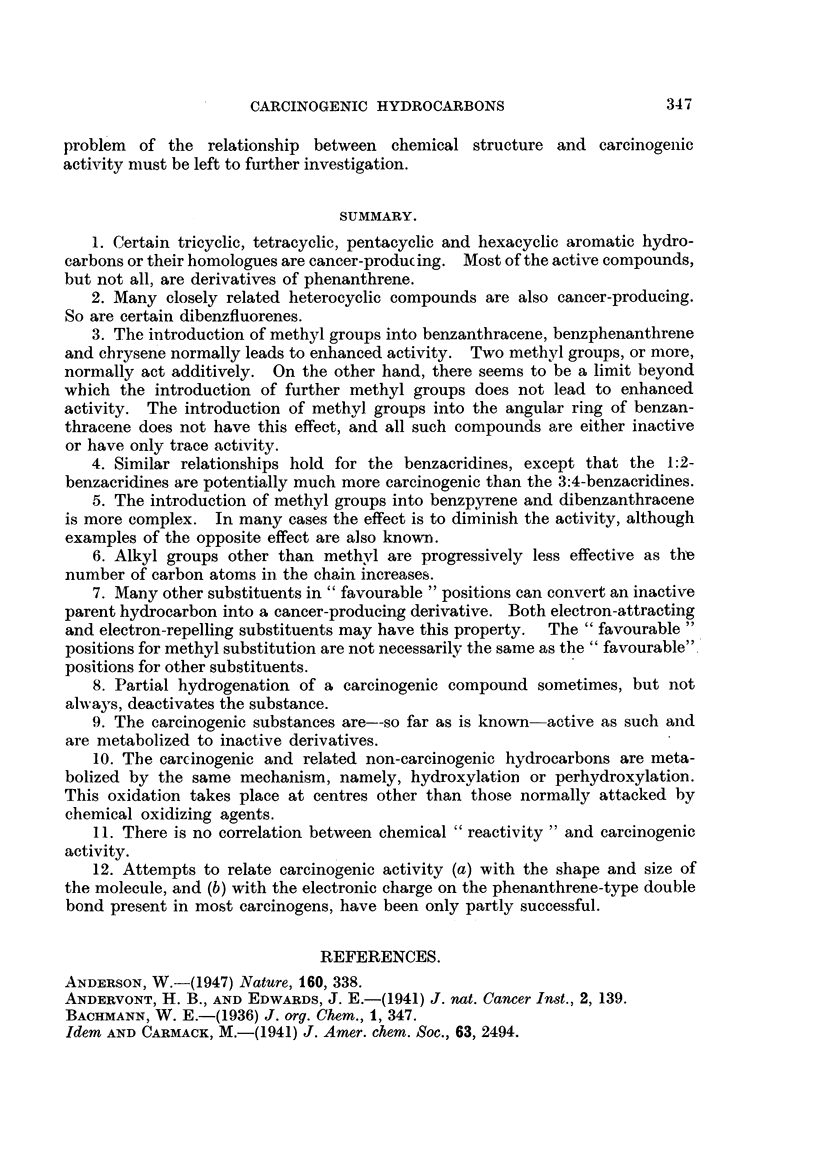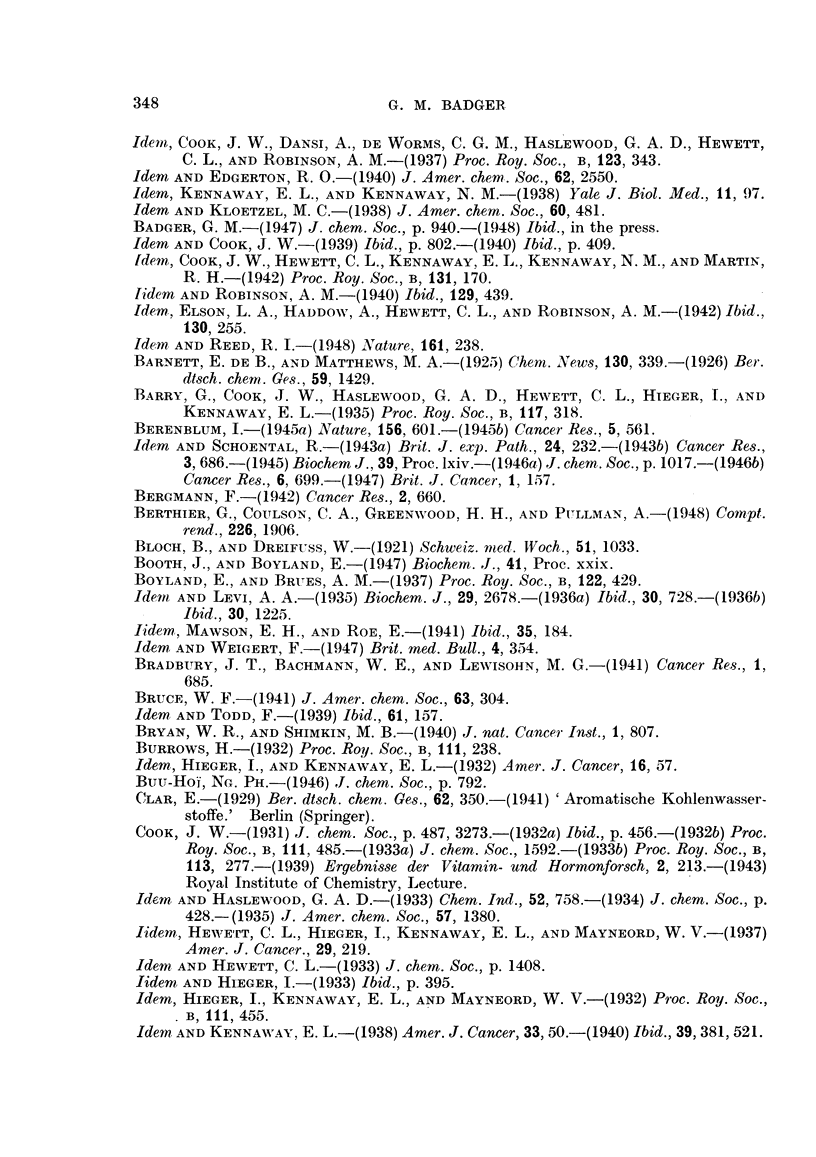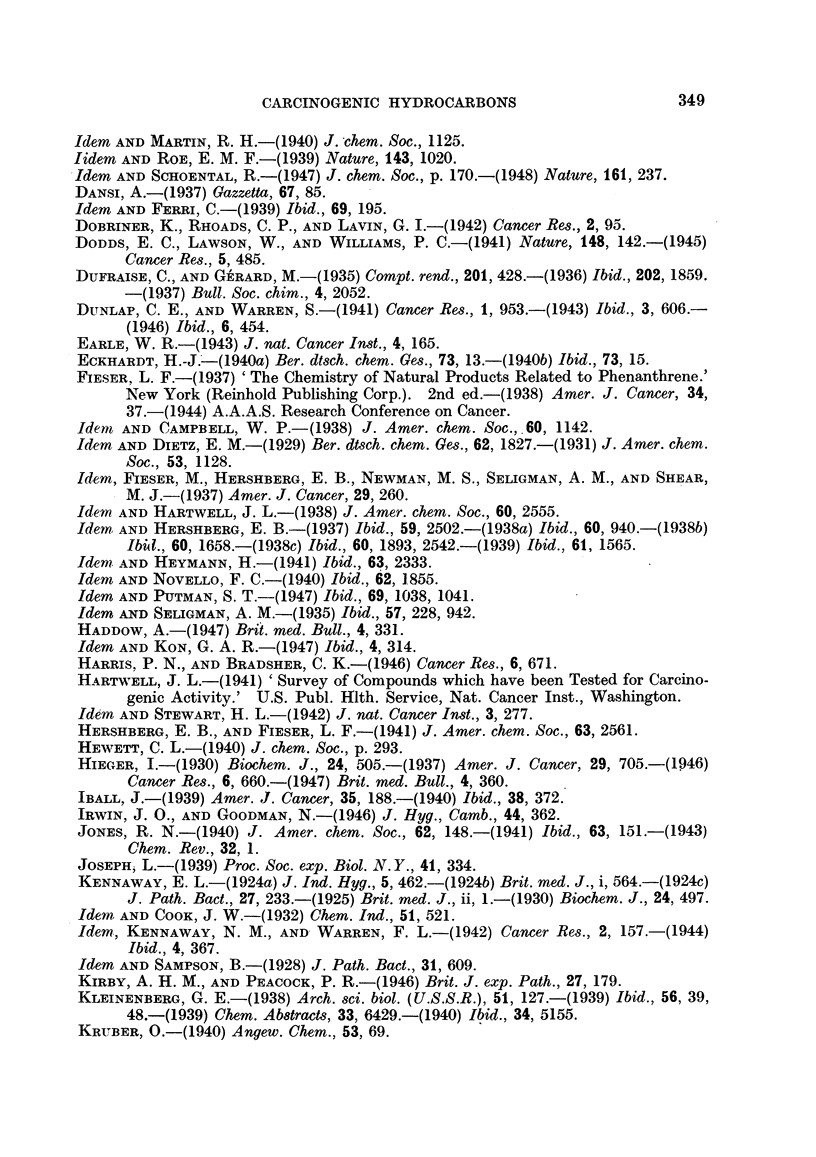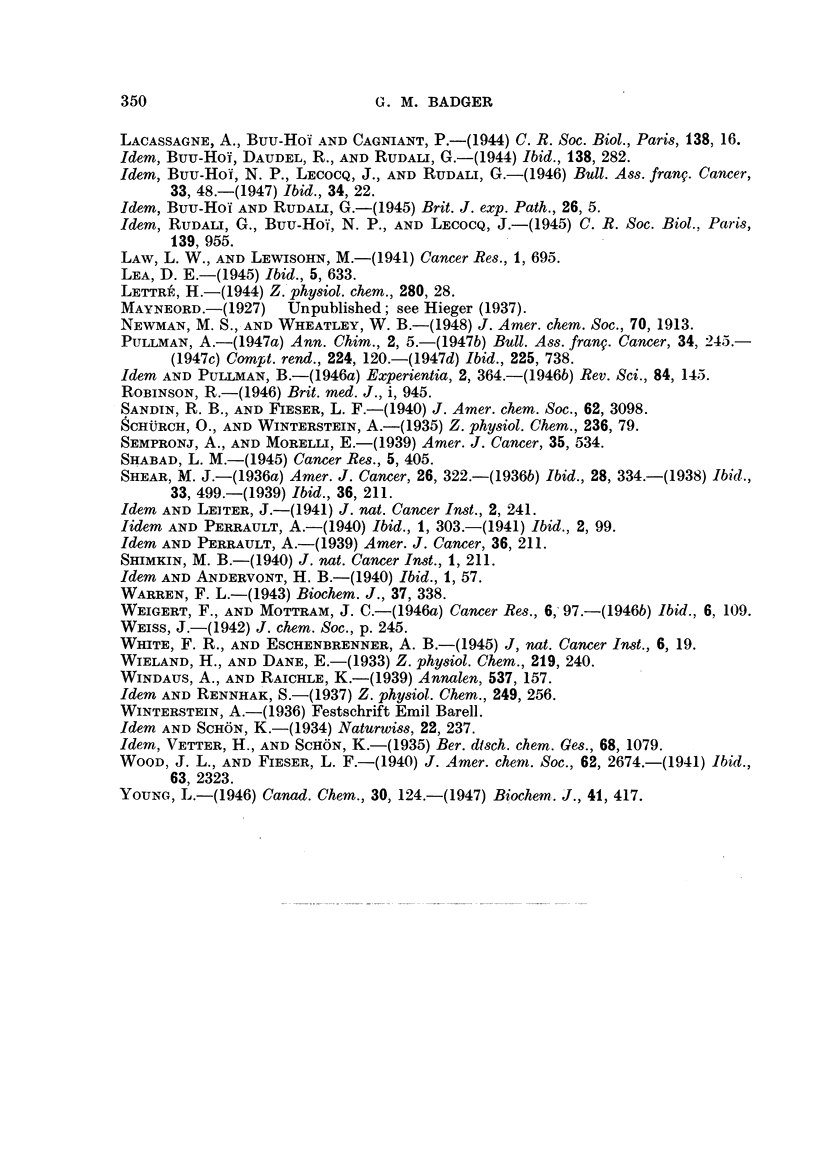# The Carcinogenic Hydrocarbons: Chemical Constitution and Carcinogenic Activity

**DOI:** 10.1038/bjc.1948.37

**Published:** 1948-12

**Authors:** G. M. Badger


### BRITISH JOURNAL OF CANCER

VOL. II DECEMBER, 1948 NO. 4

### THE CARCINOGENIC HYDROCARBONS: CHEMICAL CONSTITUTION AND CARCINOGENIC ACTIVITY.

G. M. BADGER.*

*From the Chemistry Department, University of Glasgow.*

Received for publication August 20, 1948.

IT is less than twenty years since it was first clearly demonstrated that certain pure polycyclic aromatic hydrocarbons have the property of inducing cancerous growths when applied, in solution, to the skin of animals. During the intervening years a very considerable number of synthetic cancer-producing substances has been prepared, and a very potent cancer-producing agent present in coal tar has been isolated in a pure state. For reviews see Cook, Haslewood, Hewett, Hieger, Kennaway, and Mayneord (1937); Cook and Kennaway (1938 and 1940); Fieser (1937 and 1938); Cook (1939 and 1943); Haddow (1947); Haddow and Kon (1947); Fieser, Fieser, Hershberg, Newman, Seligman and Shear (1937).

Apart from the polycyclic aromatic hydrocarbons, several other types of organic and inorganic compounds have the property of initiating cancers. The most important are: various azo compounds; certain amino stilbenes, and compounds related to 2-aminofluorene. Certain types of tumour have also been induced with oestrogenic hormones, with carbon tetrachloride, with ethyl carbamate (" urethane "), and by other substances. Hartwell (1941) has published a " Survey of compounds which have been tested for carcinogenic activity." This comprehensive work covers the literature through 1939, and includes data on 696 different chemical compounds, of which 169 were reported to be carcinogenic.

It is probably true to say that the first stage in the investigation of the carcinogenic substances is now over, and that future work must be directed towards the study of (*a*) the fate of such substances in the animal body, (*b*) the relationship, if any, between the known carcinogens and " spontaneous " cancer in humans, and (*c*) the mode of action of the carcinogens. In connection with the latter, the study of the relationship between chemical constitution and carcinogenic activity is of obvious importance. It was made clear from the early work that although relatively small changes in the structure of a carcinogen frequently converted it into an inactive derivative, major alterations could sometimes be carried out with but little change in activity. For this reason there was, at first, little or no attempt to produce an all-embracing theory. In recent years, however, there have been many attempts to find the relationship between chemical constitution and carcinogenic activity, and it appeared opportune to examine the available data on this subject. The present review is confined to the poly-

* I.C.I. Research Fellow.

cyclic aromatic compounds, their heterocyclic analogues, and other closely related substances.

### HISTORICAL.

The first detailed investigations into the nature of the carcinogenic factor in coal tar were made by Bloch and Dreifuss (1921), who found that it is concentrated in the high boiling fractions, that it is free from nitrogen, arsenic and sulphur, and that it forms a stable picrate with picric acid. These results were extended by Kennaway (1924*a*, *b*, *c* ; 1925) and Kennaway and Sampson (1928), who prepared artificial tars both from complex organic materials, such as skin, hair, yeast and cholesterol, and from hydrocarbons, such as acetylene and isoprene. The latter tars were prepared by passing the hydrocarbon, with hydrogen, through a strongly heated tube. All this work indicated that the carcinogenic agent in tar is a complex aromatic hydrocarbon. Many of the known constituents of coal tar were accordingly tested by application of a solution of the pure hydrocarbon to the skin of mice ; but all the compounds tested were found to be inactive.

The first clue as to the nature of the carcinogenic factor was provided in 1927 by Mayneord, who observed that the same characteristic fluorescence bands were to be found in various carcinogenic tars. He found also that the complex mixture obtained by Schroeter by the action of aluminium chloride on tetrahydronaphthalene, which had no connection with any form of tar, and was carcinogenic (Kennaway, 1930), showed this same spectrum. Details of the fluorescence spectrum of several tar fractions and mineral oils, and of many pure compounds, were given by Hieger (1930). The fluorescence bands of the carcinogenic mixtures were found to be at 4000, 4180 and 4400A, and Hieger observed that pure 1:2-benzanthracene has a very similar spectrum, although the bands are shifted towards shorter wave lengths.

In 1929 Clar described the synthesis, by simple reactions, of a number of complex hydrocarbons related to 1:2-benzanthracene. These compounds were also prepared, in London, and submitted to biological test. Other benzanthracene derivatives were prepared by Cook in an attempt to reproduce exactly the fluorescence spectrum of the carcinogenic tars. As a result of this work 1:2:5:6-dibenzanthracene (I), 3′-methyl-1:2:5:6-dibenzanthracene and 6-*iso*propyl-1:2-benzanthracene (II) were found to be cancer-producing (Cook, Hieger, Kennaway and Mayneord, 1932 ; Clar, 1929 ; Cook, 1931, 1932*a*, 1932*b* ; Fieser and Dietz, 1929).

I.

II.

Several other related compounds were also found to be cancer-producing, but none of these synthetic compounds was identical with the carcinogenic factor present in coal tar. All were found to give fluorescence bands more or less displaced from those observed with the carcinogenic tars. A strongly carcinogenic crystalline fraction was separated from two tons of pitch by a lengthy series of purification processes, guided by a study of the fluorescence spectrum of each fraction (Hieger, 1937). From this fraction a compound was isolated (Cook, Hewett and Hieger, 1933), which was found to be identical with the new compound 3:4-benzpyrene (III) synthesized by Cook and Hewett. (In accordance with the " Patterson " system this compound was designated 1:2-benzpyrene in the original paper, but was later renamed 3:4-benzpyrene to conform to the older " Richter " system of numbering the pyrene molecule. Both systems of numbering pyrene are in common use at the present time. The Patterson system is used by both *Chemical Abstracts* and *British Abstracts*, but the older Richter system still appears to be the method of choice for most original journals.) The synthesis was achieved by condensing pyrene with succinic anhydride, reducing the

III.

IV.

resulting keto acid to γ-1-pyrenylbutyric acid, cyclizing, reducing the resulting ketone, and finally, dehydrogenating. This method did not establish the structure rigidly, so the isomeric 1:2-benzyprene (designated 4:5-benzpyrene in the original paper) was also synthesized by the same series of reactions from *s*-hexahydropyrene. This latter synthesis can only lead to 1:2-benzpyrene, and as there are only two possible benzpyrenes, the structure of both products was confirmed. The identity of the synthetic 3:4-benzypyrene and the material isolated from coal tar was confirmed by mixed melting-point, by comparison of the fluorescence spectra, and it was also shown that both specimens were equally potent carcinogens.

The amount of 3:4-benzpyrene in coal tar has been variously estimated. According to Kruber (1940), 132 kg. of ordinary coal tar pitch contains 1 g. ; but Winterstein (1936) obtained 2·5 g. of almost pure 3:4-benzpyrene from 50 kg. of tar which contained 3 kg. of material boiling above 450°. According to Berenblum and Schoental (1943*a*), who used a spectrographic method of estimation, coal tar may contain as much as 1·5 per cent of 3:4-benzpyrene, and a simple method of extraction, by which Berenblum (1945*a*) isolated 75 mg. of almost pure material from 10 g. of crude coal tar distillate (b.p. 200–240°/0·1 mm.), has recently been devised.

Recent work by Berenblum and Schoental (1947) has shown conclusively that coal tar contains carcinogens in addition to 3:4-benzpyrene. One of these is more potent to the rabbit's skin than to that of the mouse, and this evidently accounts for the fact that tumours in rabbits are more readily produced with coal tar than with 3:4-benzpyrene. These additional carcinogenic factors present in coal tar have not yet been isolated in a pure state, although there is little doubt that they also belong to the class of polycyclic aromatic hydrocarbons. In this connection it should be emphasized that the isolation of 3:4-benzpyrene was very greatly facilitated by the fact that this compound possesses a characteristic fluorescence which is many times more intense than that of the other compounds from which it has to be separated (Berenblum and Schoental, 1946*a*).

Following the discovery of the synthetic carcinogens, and of benzpyrene, attention was concentrated on the possibility that chemical carcinogens may play a part in the initiation of " spontaneous " human cancers, other than those known as " occupational " cancers. Kennaway and Cook (1932) suggested that carcinogenic polycyclic aromatic hydrocarbons may arise from certain sterols by some abnormal mechanism. Indeed, Cook (1933*b*) predicted that 20-methylcholanthrene (VIII), prepared by a series of reactions from deoxycholic acid (V) (Wieland and Dane, 1933 ; Cook and Haslewood, 1933, 1934, 1935) would prove to be carcinogenic before tests had been carried out. This prediction was substantiated, and methylcholanthrene was found to be a very potent cancer-producing substance (Barry, Cook, Haslewood, Hewett, Hieger and Kennaway, 1935). Methylcholanthrene has also been prepared from cholic acid, and from cholesterol. It was prepared synthetically by Fieser and Seligman (1935).

V. (Deoxycholic acid.)　　VI. (12-Ketocholanic acid.)

VII. (Dehydronorcholene.)　　VIII. (20-Methylcholanthrene.)

As it was first prepared from sterols, methylcholanthrene retains the numbering of the sterol ring system. It is clear, however, that it is most conveniently considered as a 1:2-benzanthracene derivative (A) substituted in positions 6, 5, and 10. In *Chemical Abstracts* methylcholanthrene is numbered as (B), and this method of numbering has been used in some original papers.

(A)

(B)

In describing the biological results with methylcholanthrene, Barry *et al.* (1935) concluded : " Methylcholanthrene thus establishes a clear connecting link between the carcinogenic hydrocarbons and the sterols and bile acids, and it is of great interest that the changes by which it is obtained from deoxycholic acid are all reactions of the type which are known to occur normally in the animal body, although there is no evidence that this particular sequence of changes involved in the formation of methylcholanthrene does actually occur in nature."

The possibility that chemical compounds of this type may play a part in the initiation of " spontaneous " human cancers remains a speculation, and a discussion of the evidence both for and against such an hypothesis is outside the scope of the present review. Carcinogenic factors, of unknown structure, do occur in human tissues, and this line of research is being actively pursued by several workers. For a review see Hieger (1947).

The discovery of methylcholanthrene further stimulated synthetic work, especially among di- and poly-substituted benzanthracenes. Other ring systems have also been investigated, and it is now clear that carcinogenic activity may be found in derivatives of many different ring systems, including many heterocyclic systems.

### RELATIVE POTENCY OF CARCINOGENS.

Two techniques have been most extensively used for testing polycyclic aromatic hydrocarbons for carcinogenic activity. The first of these involves the application of the hydrocarbon, in 0·3 per cent solution in benzene, to the interscapular region of stock mice twice weekly (Cook, Hieger, Kennaway and Mayneord, 1932). Other solvents, including tetralin, xylene and acetone, have been used, and more dilute solutions are sometimes advantageous (Bachmann, Kennaway and Kennaway, 1938). Further, pure strain mice are sometimes preferred to stock mice. This method of test results, after a more or less prolonged latent period, in the appearance of papillomas and epitheliomas.

The second method, which gives rise to sarcomas, involves the injection of the carcinogen subcutaneously, or intraperitoneally. The crystalline material is sometimes injected in the form of a pellet of about 5 mg., but often, a solution of the hydrocarbon in lard, cholesterol, sesame oil, tricaprylin, or other such solvent, is injected. The quantity administered has varied from author to author ; and more than one injection has often been given, especially in the case of the less active compounds. Both stock mice and mice of pure strains have been used (Burrows, Hieger and Kennaway, 1932 ; Burrows, 1932 ; Shear, 1936*a*).

Both methods of administration sometimes result in the appearance of a number of tumours, e.g. of the liver, lungs, etc., not at the site of application. Such tumours are normally omitted from discussions of the relative potency of carcinogens of the polycyclic aromatic type—mainly because the data are insufficient for their correct appraisal (Badger, Cook, Hewett, Kennaway, Kennaway, Martin and Robinson, 1940). In the case of the carcinogenic azo compounds, and with certain other classes of compound not discussed in this review, tumours do not normally appear at the site of application, and the remote tumours are the only means of comparison of carcinogenic potency.

All carcinogens do not have the same potency. Tumours appear in treated animals (usually mice) only after a latent period, which varies from about a month or two for very active compounds, to nearly two years for the very feebly active compounds. Very active substances also induce tumours in a high percentage of mice, while substances of slight activity produce tumours in only a few animals after a prolonged latent period.

Many attempts have been made to devise a system suitable for the accurate comparison of the potency of carcinogens. In many ways the best method is that due to Iball (1939), who introduced a *carcinogenic index*, defined by the relationship :

$$\text{Carcinogenic index} = \frac{\text{Percentage number of tumours}}{\text{Average latent period in days}}.$$

The percentage number of tumours was obtained from the number of animals bearing tumours, and the number of animals alive when the first tumour appeared. This method has the advantage that it eliminates animals which die too early to be affected by the compound under test. In this method of calculating the relative potency of carcinogens (and also in most other methods which have been suggested) papillomas are given the same weight as epitheliomas.

Fieser (1938) has also called attention to the importance of the latent period. He critically examined the data for the more important carcinogens, and attempted to compare the relative potencies by comparing average induction periods, weighted according to the number of tumours produced in any given experiment.

In a more recent study of the problem Berenblum (1945*b*) has suggested a system of *carcinogenic grades*, from XII to I, the grade XII being applied to compounds of very pronounced carcinogenic activity, and the grade I being applied to compounds with only trace activity. This system is also based on the latent period of carcinogenesis, and the carcinogenic grade is obtained from a diagram in which the grade (I to XII) is plotted against the latent period in weeks. The latter is on a logarithmic scale in order to eliminate the prominence which would otherwise be given to compounds of low potency.

Interesting discussions and information relative to the determination of carcinogenic potency are also to be found in papers by Shimkin (1940), Bryan and Shimkin (1940), and by Lea (1945). A comprehensive paper on the statistical treatment of measurements of the carcinogenic properties of tars and mineral oils has been published by Irwin and Goodman (1946).

None of these methods has been used extensively by other authors, and in only a very few papers has sufficient data been published for any of them to be used with reasonable accuracy. Iball's carcinogenic index has, however, been used in a few cases (Shimkin and Andervont, 1940 ; Badger, Elson, Haddow, Hewett and Robinson, 1942 ; Lacassagne, Buu-Hoï, Lecocq, and Rudali, 1946). Berenblum's carcinogenic grading is somewhat simpler, but in the opinion of the present author any attempt to compare the relative potency of different hydrocarbons by the use of twelve grades is apt to give a greater semblance of accuracy than is justified, especially when it is desired to compare the results from different laboratories, possibly using different animal strains and different techniques. All attempts to study the relationship between chemical constitution and biological activity must be limited by the inaccuracies inherent in all biological assays, and specific reference to the extreme difficulties in testing carcinogenic compounds has been made by Hartwell (1941, p. 9).

For these reasons the grading of potency in the present paper has been made as simple as possible. The potency of the compounds to both epithelial cells and to cells of connective tissue have both been classified as follows :

| | | |
|---|---|---|
| ++++ | signifies | very marked carcinogenic activity. |
| +++ | ,, | marked carcinogenic activity. |
| ++ | ,, | moderate carcinogenic activity. |
| + | ,, | slight carcinogenic activity. |
| 0 | ,, | inactive. |

The working of the system in practice may be observed from Table I, in which the carcinogenic index of Iball, the carcinogenic grade of Berenblum, and the system adopted in the present paper are compared for 10 well-known compounds which have been tested by application to the skin of mice.

TABLE I.—*Relative Potency of Carcinogens to the Skin of Mice ; Comparison of Different Systems of Grading.*

| Compound. | Per cent tumours. | Average latent period (days). | Iball's index. | Berenblum's grade. | This paper. |
|---|---|---|---|---|---|
| 9:10-Dimethyl-1:2-benzanthracene | 65 | 43 | 151 | X | ++++ |
| 20-Methylcholanthrene | 88·5 | 109 | 80 | VIII | ++++ |
| 3:4-Benzpyrene | 89·5 | 119 | 75 | VIII | ++++ |
| 2-Methyl-3:4-benzphenanthrene | 75 | 155 | 48 | VII | +++ |
| 10-Methyl-1:2-benzanthracene | 66·5 | 147 | 45 | VI ? | +++ |
| 5-Methyl-1:2-benzanthracene | 87·5 | 317 | 28 | V | ++ |
| 1:2:5:6-Dibenzanthracene | 63 | 239 | 26 | VI | ++ |
| 3:4-Benzphenanthrene | 67 | 387 | 17 | V | + |
| 1:2:5:6-Dibenzacridine | 24 | 350 | 7 | 4 | + |
| 3:4:5:6-Dibenzacridine | 39·3 | 357 | 11 | IV | + |

This method of grading which is adopted has the very great advantage of simplicity. Even so, it is difficult, in some cases, to be certain of the most accurate grading, as the published data are often so meagre. Furthermore, in view of the difficulties of biological assay, the probable or expected error in grading is at least one + symbol.

### THE STRUCTURE OF THE CARCINOGENIC COMPOUNDS.

### (a) *Polycyclic aromatic hydrocarbons, and the effect of methyl groups.*

As has already been pointed out, the resemblance between the fluorescence spectra of the carcinogenic tars and that given by pure 1:2-benzanthracene (IX) provided the first clue as to the nature of the active principle. It also provided the starting-point for the synthetic approach, which led to the testing of 1:2:5:6-dibenzanthracene, to the synthesis and testing of 6-*iso*propyl-1:2-benzanthracene, and of other compounds. Since then many of the homologues of the tricyclic, the tetracyclic, the pentacyclic and a few hexacyclic aromatic hydrocarbons have been tested, and examples of each class of compound have been shown to possess cancer-producing activity. In this and in the subsequent sections references to tests for carcinogenic activity are to be found in the tables. Only in the cases of compounds not listed in tables have references to the literature been appended in the text.

Anthracene, phenanthrene and a few simple derivatives were tested in the very early experiments (Hartwell, 1941) and found to be inactive. It is only recently that two simple derivatives have been shown to possess slight but definite carcinogenic activity to the skin of mice. These active homologues are 1:2:3:4-tetramethylphenanthrene, and 9:10-dimethylanthracene, and they are the simplest examples of carcinogen yet found among the polycyclic aromatic hydrocarbons (Badger, Cook, Hewett, Kennaway, Kennaway and Martin, 1942; Kennaway, Kennaway and Warren, 1942).

There are six possible tetracyclic aromatic hydrocarbons composed entirely of aromatic rings, and each has been investigated. Only 3:4-benzphenanthrene (X) has been shown to possess significant, if slight, activity. Tumours have occassionally been attributed to chrysene (XI), but these are probably due to impurities almost invariably associated with this hydrocarbon. 1:2-Benzanthracene (IX) has also given one or two tumours in very extensive tests, but it is doubtful if these are significant. Triphenylene (XII), pyrene (XIII) and naphthacene (XIV) appear to be inactive.

IX.
1:2-Benzanthracene.

X.
3:4-Benzphenanthrene.

XI.

Chrysene.

XII.

Triphenylene.

XIII.

Pyrene.

XIV.

Naphthacene.

All the twelve possible monomethyl derivatives of 1:2-benzanthracene have been prepared and tested, many of them both by application to the skin and by subcutaneous injection. There is no doubt that the introduction of a methyl group into one of the " favourable " positions has a very pronounced effect, and may give rise to a very potent cancer-producing substance. The most active derivatives are those in which a methyl group has been introduced into positions 10-, 9-, or 5-. The introduction of a methyl group into positions 6-, 7-, 8-, 3- or 4- serves to produce a moderately to very slightly active substance, but methyl groups in the angular ring, that is in positions 1′-, 2′-, 3′- or 4′-, do not seem to have this effect. 4′-Methyl-1 : 2-benzanthracene may have trace activity, but 1′-methyl-, 2′-methyl-, and 3′-methyl-1:2-benzanthracenes appear to be entirely inactive (Table II).

The effect of additional methyl groups is, in general, to give rise to more potent compounds. As a general rule, dimethylbenzanthracenes are more potent than either of the monomethyl derivatives to which they are related. 9:10-Dimethyl-1:2-benzanthracene is especially active to the skin of mice, and produces many tumours within a remarkably short latent period. 5:9-Dimethyl- and 5:10-dimethyl-1:2-benzanthracenes are also remarkably active cancer-producing substances, and 5:6-dimethyl-1:2-benzanthracene is also more active than either of the monomethyl derivatives to which it is related. On the other hand, there appear to be some exceptions to this generalization. 5:8-Dimethyl- and 3:9-dimethyl-1:2-benzanthracenes appear to be inactive, at least when administered by injection. Furthermore, it is also of some interest that all the dimethylbenzanthracenes having a methyl group in the angular ring are entirely inactive.

A few polysubstituted benzanthracenes have also been examined. 5:9:10-Trimethyl- and 6:9:10-trimethyl-1:2-benzanthracenes are extremely active carcinogens, as might be expected. On the other hand, 5:6:9:10-tetramethyl-1:2-benzanthracene appears to be somewhat less active than either of the trimethyl, *meso*-substituted benzanthracenes to which it is related. It is possible that there is an optimum number of methyl groups which can be introduced and which lead to an increase in potency. This possibility, though widely accepted, has not been extensively investigated.

The cholanthrenes, and related compounds, are most conveniently considered as substituted benzanthracenes, although the sterol numbering often used tends to confuse the relationship. Cholanthrene, 20-methylcholanthrene, 22-methyl- and 23-methylcholanthrene have been prepared and tested. They may be considered as benzanthracenes substituted in positions 10-, 5- and one other position. All were found to be very active, but there is little doubt that 20-methylcholanthrene is the most active, then cholanthrene, then 22-methylcholanthrene, and then least active, 23-methylcholanthrene. Law and Lewisohn (1941) have also examined these compounds by subcutaneous injection in " strain C " mice, and observed that 20-methylcholanthrene and 22-methylcholanthrene are significantly more potent in inducing lung tumours than the parent hydrocarbon, cholanthrene, and that 23-methylcholanthrene is significantly less potent than cholanthrene.

In view of the special interest of methylcholanthrene many related compounds were tested. One line of approach was to vary the positions of attachment of the dimethylene bridge. Acenaphthanthracene (4′:3-ace-1:2-benzanthracene) is only slightly active, 8:9-ace-1:2-benzanthracene is moderately active, 4:10-ace-1:2-benzanthracene is a potent carcinogen, but cholanthrene (5:10-ace-1:2-benzanthracene) is the most potent. These results again indicate the pre-eminence of positions 5- and 10- for the development of carcinogenic activity. It is surprising that 7-methyl-8:9-ace-1:2-benzanthracene is inactive.

It was at first thought that the dimethylene bridge might have some special significance for the development of carcinogenic activity, but this has since been disproved. 5:10-Dimethyl-1:2-benzanthracene, and cholanthrene are approximately equally potent; similarly, 4:10-ace-1:2-benzanthracene and 4:10-dimethyl-1:2-benzanthracene are moderately, and about equally, potent. The dimethylene bridge present in these compounds therefore appears to be approximately equivalent to two methyl groups in the same positions.

There are six possible monomethyl derivatives of 3:4-benzphenanthrene, and five of these have been prepared and tested. 5-Methyl-3:4-benzphenanthrene has only recently been prepared (Newman and Wheatley, 1948). It is a remarkable feature of the benzphenanthrenes, which has not yet been explained, that these compounds are all very much less effective in producing cancer when administered by injection than when applied to the skin. 3:4-Benzphenanthrene (X) itself is slightly active to the skin of mice, but has proved ineffective when administered subcutaneously. 2-Methyl-3:4-benzphenanthrene is a potent carcinogen when applied to the skin, but is only very slightly active when administered by injection. 1-Methyl-3:4-benzphenanthrene is moderately active to the skin, and seems to be inactive by injection. The remaining monomethyl derivatives are only slightly active. Only a few dimethyl derivatives have been tested. These include 6:7-dimethyl-, and 2:9-dimethyl-3:4-benzphenanthrene, which have been shown to be inactive by injection ; but since these compounds have not been tested on the skin no inferences can be drawn.

All the monomethyl chrysenes have been prepared (Bachmann and Edgerton, 1940), but all do not appear to have been tested. Work in this field is sometimes difficult to follow, as two systems of numbering the chrysene molecule are about equally used (Table IV). In America the Patterson system is used, but in Europe the Richter system is still commonly used, and is used in this report. The Patterson numbers are given in parenthesis. 1-Methylchrysene (5-) is a potent carcinogen, and 2-methyl-(6-), and 6-methylchrysene (4-) are slightly active. These results apply only to tests by subcutaneous injection, and since there is some evidence that the alkylchrysenes, like the alkylbenzphenanthrenes, are more potent when applied to the skin, the relative carcinogenic activities of these derivatives should be accepted with caution. 1:2-Dimethylchrysene (5:6-), for example, is a moderately active carcinogen to the skin of mice, but only very feebly active when administered by injection. 6:7-Dimethylchrysene (4:5-) and 6:7-methylenechrysene (4:5-) are feebly active by injection, but have not been tested by application to the skin.

Relatively little work has been done on the homologues of triphenylene, naphthacene, and pyrene. Triphenylene itself is not carcinogenic, and the 1-methyl- and 1:4-dimethyl-derivatives also appear to be inactive, at least when administered by injection (Shear and Leiter, 1941). Naphthacene derivatives are very readily photo-oxidized, and are not therefore very suitable for test (23rd Annual Report, *B.E.C.C.*, 1946, p. 109). 2-*iso*Propylnaphthacene has been tested for comparison with its cancer-producing isomer, 6-*iso*propyl-1:2-benzanthracene : it did not induce tumours (Barry *et al.*, 1935). Pyrene and 4-methylpyrene are also inactive (Barry *et al.*, 1935 ; Badger *et al.*, 1940).

There are fifteen possible pentacyclic aromatic hydrocarbons composed entirely of benzene rings, and all have been tested for cancer-producing activity (Barry *et al.*, 1935). 1:2:5:6-Dibenzanthracene (I) was the first pure synthetic substance shown to be carcinogenic ; 1:2:7:8-dibenzanthracene has given a few tumours in very extensive tests, and is only very feebly active ; 1:2:3:4-dibenzanthracene seems to be inactive. 3:4-Benzpyrene (III) is a very potent carcinogen, but 1:2-benzpyrene (IV) is inactive. 1:2:5:6-Dibenzphenanthrene (XV) and 1:2:3:4-dibenzphenanthrene (XVI) are moderately active, and the remaining pentacyclic hydrocarbons are inactive. It is interesting that of the fifteen possible pentacyclic hydrocarbons, thirteen may be considered as derived from phenanthrene, and only five of these have given tumours.

XV.

XVI.

A few homologues of 1:2:5:6-dibenzanthracene have been examined with interesting results. 2′-Methyl-, 3′-methyl-, and 4-methyl-1:2:5:6-dibenzanthracenes are all weakly to moderately active (Cook, 1932*b* ; Barry *et al.*, 1935). 1′:9-Methylene-1:2:5:6-dibenzanthracene (XVII) is moderately active (Shear, 1936*a*) ; but 9-methyl-1:2:5:6-dibenzanthracene (XVIII) is an extremely potent carcinogen (Shear, Leiter and Perrault, 1940). It is therefore surprising that 9:10-dimethyl-1:2:5:6-dibenzanthracene is only very feebly active, having pro-

XVII.

XVIII.

duced only one papilloma. This result, which appears to run contrary to the 1:2-benzanthracene series, was observed in the early work (Cook, 1932*b*), and materially delayed the recognition of the importance of the *meso* substitution in the related series. It is also noteworthy that although 9:10-dimethyl-1:2:3 4-dibenzanthracene is inactive, as might be expected, 9:10-dimethyl-1:2:7:8-dibenzanthracene is a potent cancer-producing substance (23rd Annual Report, *B.E.C.C.*, 1946, p. 107). These observations have been offered in support of the hypothesis linking carcinogenic activity with an activated phenanthrene-type bond (Haddow, 1947).

The effect of methyl groups on the activity of 3:4-benzpyrene is also of considerable interest as, here again, the effect seems to run counter to that which might be expected from a consideration of the methylbenzanthracenes. 2′-Methyl- and 3′-methyl-3:4-benzpyrenes appear to be inactive, at least when administered by injection, although these positions of substitution correspond to the 7- and 6- positions in benzanthracene. Furthermore, 4′-methyl- and 6-methyl-3:4-benzpyrene are considerably less active than the parent hydrocarbon (III). Again, 9-methyl-3:4-benzpyrene and 5-methyl-3:4-benzpyrene appear to have the same activity as the parent hydrocarbon. The 9- position corresponds to the 3′- position in benzanthracene, and 3′-methyl-1:2-benzanthracene is inactive.

Only a few examples of hexacyclic aromatic hydrocarbons have been examined, Both 1:2:3:4-dibenzpyrene and 3:4:8:9-dibenzpyrene (XIX and XX) are active,

TABLE II.—*Methylbenzanthracenes, Acebenzanthracenes and Cholanthrenes.*

Benzanthracene numbering.*

Cholanthrene numbering.†

| Compound. | Carcinogenic activity. Skin. | References. | Subcutaneous tissue. | References. |
|---|---|---|---|---|
| 1:2-Benzanthracene | 0 | a | 0 | e |
| 1′-Methyl- | 0 | b | 0 | b, c, e |
| 2′-Methyl- | 0 | a | .. | .. |
| 3′-Methyl- | 0 | a | .. | .. |
| 4′-Methyl- | 0 | b | .. | .. |
| 3-Methyl- | + | a, b | ++ | b |
| 4-Methyl- | + | a | ++ | e |
| 5-Methyl- | ++ | a | ++ | d |
| 6-Methyl- | + | a | .. | .. |
| 7-Methyl- | + | a, b | + | b, d |
| 8-Methyl- | + | b | 0 | e |
| 9-Methyl- | ++ | b | +++ | d |
| 10-Methyl- | +++ | b | ++++ | d |
| 1′:10-Dimethyl- | .. | .. | 0 | f |
| 2′:6-Dimethyl- | 0 | a, g | 0 | a, g |
| 2′:7-Dimethyl- | 0 | a | .. | .. |
| 3′:6-Dimethyl- | 0 | a | .. | .. |
| 3′:7-Dimethyl- | 0 | a | .. | .. |
| 3:9-Dimethyl- | .. | .. | 0 | e |
| 4:9-Dimethyl- | .. | .. | +++ | e |
| 4:10-Dimethyl- | .. | .. | ++ | f |
| 5:6-Dimethyl- | +++ | a | .. | .. |
| 5:8-Dimethyl- | .. | .. | 0 | h |
| 5:9-Dimethyl- | .. | .. | ++++ | d |
| 5:10-Dimethyl- | .. | .. | ++++ | d |
| 6:7-Dimethyl- | + | a | .. | .. |
| 8:10-Dimethyl- | .. | .. | +++ | h |
| 9:10-Dimethyl- | ++++ | b | +++ | b, d, f, h |
| 4′:3-Ace- | + | b | ++ | b |
| 4:10-Ace- | .. | .. | +++ | d, h |
| 5:10-Ace-(Cholanthrene) | ++++ | j | ++++ | k, l |
| 8:9-Ace- | .. | .. | ++ | k |
| 5:9:10-Trimethyl- | ++++ | b, m | +++ | b |
| 6:9:10-Trimethyl- | ++++ | b, g | ++ | b |
| 20-Methylcholanthrene | ++++ | j | ++++ | l |
| 22-Methylcholanthrene | .. | .. | ++++ | l |
| 23-Methylcholanthrene | .. | .. | ++++ | l |
| 7-Methyl-8:9-ace- | .. | .. | 0 | k |
| 5:6:9:10-Tetramethyl- | +++ | b | + | b |

and so also is 7-methyl-1:2:3:4-dibenzpyrene. All the other hexacyclic aromatic hydrocarbons which have been tested have failed to give rise to tumours (Kleinenberg, 1938, 1939, 1940; Bachmann, Cook, Dansi, de Worms, Haslewood, Hewett and Robinson, 1937).

XIX.

XX.

TABLE III.—*Methylbenzphenanthrenes.*

| Compound. | Carcinogenic activity. Skin. | References. | Subcutaneous tissue. | References. |
|---|---|---|---|---|
| 3:4-Benzphenanthrene | + | a, c | 0 | c, d |
| 1-Methyl- | ++ | c | 0 | c |
| 2-Methyl- | +++ | b, c | + | c, d |
| 6-Methyl- | + | c | + | c |
| 7-Methyl- | + | c | 0 | c |
| 8-Methyl- | + | c | 0 | c |
| 2:9-Dimethyl- | .. | .. | 0 | d |
| 6:7-Dimethyl- | .. | .. | 0 | e |

a, Barry, Cook, Haslewood, Hewett, Hieger and Kennaway (1935); b, Bachmann, Cook, Dansi, de Worms, Haslewood, Hewett and Robinson (1937); c, Badger, Cook, Hewett, Kennaway, Kennaway, Martin and Robinson (1940); d, Shear and Leiter (1941); e, Shear (1938).

* This method of numbering has been used most extensively in both the chemical and boilogical literature. It is not, however, the system which is recommended by Patterson. The Patterson numbering (A) is used in *Chemical Abstracts,* and has also been used recently in the *Journal* of the American Chemical Society.

(A).

† This is the "sterol" numbering for cholanthrene. For the other systems see footnote to formula VIII.

*References:*—a, Barry, Cook, Haslewood, Hewett, Hieger and Kennaway (1935); b, Badger, Cook, Hewett, Kennaway, Kennaway, Martin and Robinson (1940); c, Shear (1939); d, Shear (1938); e, Shear and Leiter (1941); f, Shear, Leiter and Perrault (1940); g, Badger, Cook, Hewett, Kennaway, Kennaway and Martin (1942); h, Dunlap and Warren (1946); i, Shear and Perrault (1939); j, Bachmann, Cook, Dansi, de Worms, Haslewood, Hewett and Robinson (1937); k, Shear (1936*b*); l, Bradbury, Bachmann and Lewisohn (1941); m, Hartwell and Stewart (1942).

TABLE IV.—*Methylchrysenes.*

" Richter " numbering.*

| Compound. | Carcinogenic activity. Skin. | References. | Subcutaneous tissue. | References. |
|---|---|---|---|---|
| Chrysene | 0 | a | 0 | c |
| 1-Methyl- | .. | .. | +++ | b |
| 2-Methyl- | .. | .. | + | c |
| 6-Methyl- | .. | .. | + | b |
| 4:5-Dimethyl- | .. | .. | 0 | d |
| 6:7-Dimethyl- | .. | .. | + | b |
| 1:2-Dimethyl- | ++ | e | + | b, e |

* This system of numbering is used in Europe, but the Patterson system (A) is used in America.

(A)

a, Bachmann, Cook, Dansi, de Worms, Haslewood, Hewett and Robinson (1937); b, Dunlap and Warren (1943); c, Shear and Leiter (1941); d, Shear (1938); e, Badger, Cook, Hewett, Kennaway, Kennaway, Martin and Robinson (1940).

TABLE V.—*Methylbenzpyrenes.*

| Compound. | Carcinogenic activity. Skin. | References. | Subcutaneous tissue. | References. |
|---|---|---|---|---|
| 3:4-Benzpyrene | ++++ | a, g | ++++ | a, h |
| 2′-Methyl- | .. | .. | 0 | c |
| 3′-Methyl- | 0 | b, f, i | 0 | c |
| 4′-Methyl- | .. | .. | ++ | d |
| 5-Methyl- | .. | .. | ++++ | e |
| 6-Methyl- | .. | .. | ++ | c |
| 9-Methyl- | .. | .. | ++++ | c |

a, Fieser (1938); b, Schürch and Winterstein (1935); c, Fieser and Heymann (1941); d, Shear and Perrault (1939); e, Shear, Leiter and Perrault (1940); f, Winterstein, Vetter and Schön (1935); g, Barry, Cook, Haslewood, Hewett, Hieger and Kennaway (1935); h, Shear (1936*a*); i, Fieser and Hershberg (1938*b*).

(b) *Alkyl derivatives in homologous series.*

There appears to be little doubt that the substitution of one or more methyl groups into a " favourable " position of certain polycyclic aromatic hydrocarbons can transform a non-carcinogenic hydrocarbon into a carcinogenic derivative, or can materially increase the activity of a carcinogen. It was therefore of interest to study the effect of the higher alkyl groups.

The 5-*n*-alkyl-1:2-benzanthracenes have been extensively investigated, for all the members of the series as far as the *n*-heptyl derivative have been examined, most of them by both injection, and painting on the skin. When tested by the latter method all the compounds are active, but there is a progressive diminution in potency as the number of carbon atoms in the alkyl chain increases, 5-methyl-1: 2-benzanthracene being moderately active, but the 5-*n*-heptyl derivative being only very slightly so. A plot of Iball's carcinogenic index for each compound against the number of carbon atoms in the alkyl chain has been published by Badger, Elson, Haddow, Hewett and Robinson (1942). When tested by subcutaneous injection, however, the higher members of the series (butyl, amyl, hexyl, and heptyl) failed to give tumours. 5-Methyl-1:2-benzanthracene is moderately active by this method, but comparable experiments with the ethyl and *n*-propyl derivatives do not appear to have been undertaken. 5-*iso*Propyl-1:2-benzanthracene was found to be moderately active to the skin, and slightly active by injection.

Of the 10-*n*-alkyl-1:2-benzanthracenes the methyl derivative is extremely potent, both by application to the skin and by injection. The ethyl homologue is rather less active when tested by injection, and the higher homologues have failed to give tumours when administered by this method. As these compounds have not been tested by application to the skin, it is difficult to decide whether activity is more easily lost in the 10- substituted series or in the 5- substituted compounds. It is of some interest, however, that 10-*iso*propyl-1:2-benzanthracene has been tested by both methods and appears to be completely inactive, while the 5-*iso*propyl- derivative, which has also been tested by both methods, is moderately active.

Little work has been carried out on the synthesis and testing of other homologous series of alkylbenzanthracenes. An apparent exception, however, is 6-*iso*propyl-1 : 2-benzanthracene, which appears to be more active than the methyl derivative ; but this comparison has been made only on the skin of mice, and not by injection. 3-*iso*Propyl- and 7-*iso*propyl-1:2-benzanthracenes have also been tested on the skin and found to be inactive ; the corresponding methyl derivatives are very slightly active. 20-Methylcholanthrene is rather more potent than the parent hydrocarbon, cholanthrene ; the higher members of this series, namely, the ethyl-, *iso*propyl-, and *tert.*-butylcholanthrenes are progressively less potent when tested by injection.

A short series of 2-alkyl-3:4-benzphenanthrenes has also been examined. In skin-painting experiments the 2-methyl- derivative proved very potent, and the 2-ethyl- and 2-*iso*propyl- derivatives were found to be less active. The benzphenanthrenes always show greater activity when tested on the skin than when given by injection, and the present series is no exception ; 2-methyl-3:4-benzphenanthrene was found to be only feebly active by this latter method, and the 2-*iso*propyl- derivative failed to give any tumours.

TABLE VI.—*Alkyl Derivatives in Homologous Series.*

| Compound. | Carcinogenic activity. Skin. | References. | Subcutaneous tissue. | References. |
|---|---|---|---|---|
| 5-Methyl-1:2-benzanthracene | ++ | a | ++ | e |
| 5-Ethyl- | ++ | b | .. | .. |
| 5-*n*-Propyl- | ++ | b | .. | .. |
| 5-*iso*Propyl- | ++ | c | + | c |
| 5-*n*-Butyl- | ++ | c | 0 | c |
| 5-*n*-Amyl- | ++ | c | 0 | c |
| 5-*n*-Hexyl- | + | c | 0 | d |
| 5-*n*-Heptyl- | + | d | 0 | c |
| 10-Methyl-1:2-benzanthracene | +++ | c, f | ++++ | f |
| 10-Ethyl- | .. | .. | +++ | f |
| 10-*n*-Propyl- | .. | .. | 0 | f |
| 10-*iso*Propyl- | 0 | a | 0 | f |
| 10-*n*-Butyl- | .. | .. | 0 | f |
| 10-*n*-Amyl- | .. | .. | 0 | f |
| 20-Methylcholanthrene | ++++ | b | ++++ | e |
| 20-Ethylcholanthrene | .. | .. | +++ | e, g |
| 20-*iso*Propylcholanthrene | .. | .. | ++ | g |
| 20-*t*-Butylcholanthrene | .. | .. | + | g |
| 2-Methyl-3:4-benzphenanthrene | +++ | c | + | c |
| 2-Ethyl- | ++ | c, d | .. | .. |
| 2-*iso*Propyl- | ++ | c | 0 | c |
| 2-*n*-Propyl- | ++ | d | 0 | d |

a, Barry, Cook, Haslewood, Hewett, Hieger and Kennaway (1935); b, Bachmann, Cook, Dansi, de Worms, Haslewood, Hewett and Robinson (1937); c, Badger, Cook, Hewett, Kennaway, Kennaway, Martin and Robinson (1940); d, Badger, Cook, Hewett, Kennaway, Kennaway and Martin (1942); e, Shear (1938); f, Shear, Leiter and Perrault (1940); g, Shear, Leiter and Perrault (1941).

(c) *The effect of other substituents.*

Although most of the early work was concentrated on an examination of hydrocarbons, a few derivatives carrying functional substituents were examined. 9-Amino-1:2:5:6-dibenzanthracene, and 9-methoxy-1:2:5:6-dibenzanthracene, for example, were found to be moderately active. On the other hand, the introduction of other substituents at the same position gave rise to inactive derivatives; these included the 9-nitro-, 9-hydroxy-, 9-acetoxy-, and 9-*n*-butyrylamino- substituted 1:2:5:6-dibenzanthracenes (Barry *et al.*, 1935). As has been pointed out above, 9-methyl-1:2:5:6-dibenzanthracene is a potent carcinogen, more active than the parent hydrocarbon (Shear, Leiter and Perrault, 1940).

Several substituted methylcholanthrenes have been tested (Shear, Leiter and Perrault, 1941). These include the 2-hydroxy-, 2-methoxy-, 3-hydroxy-, and 3-methoxy- derivatives, all of which are inactive. The effect of substitution on the dimethylene bridge is not so striking, however, for 15-hydroxy-20-methylcholanthrene is still moderately active, and the corresponding keto derivative is slightly active. 6-Chloro- and 6-cyano-20-methylcholanthrenes are inactive.

In the above cases the effect of the substituent (other than methyl) has been to reduce the potency of the original hydrocarbon, and in some cases to abolish the activity altogether. Several functional derivatives of 9:10-dimethyl-1:2-benzanthracene have been examined, however, and in these cases the activity of the parent hydrocarbon seems to be retained. 5-Bromo- and 5-cyano-9:10-dimethyl-1:2-benzanthracenes have about the same activity as the parent dimethylbenzanthracene (Dunlap and Warren, 1946).

Substitution in the methyl groups of this hydrocarbon does reduce the potency. 9-Methyl-10-ethoxymethyl-, 9-methyl-10-methoxymethyl-, 9:10-bishydroxymethyl-, and 9:10-bisacetoxymethyl-1:2-benzanthracenes are all appreciably less active than 9:10-dimethyl-1:2-benzanthracene (Dunlap and Warren, 1946; Badger, Cook, Hewett, Kennaway, Kennaway, Martin and Robinson, 1940).

The type of substituent is clearly of great importance, but (as with methyl groups) the position of substitution is also of considerable importance, although it must not be assumed that the " favourable " positions for polar substituents are the same as the " favourable " positions for alkyl groups. Benzpyrene and 5-methyl-3:4-benzpyrene are potent carcinogens, for example, and 5-methoxy-3:4-benzpyrene is only very slightly active, while 8-methoxy-3:4-benzpyrene is a potent carcinogen (21st Annual Report, *B.E.C.C.*, 1944, p. 56).

The activity or otherwise of the metabolic products of polycyclic aromatic hydrocarbons is of some interest (see p. 332). 4′-Hydroxy-1:2-benzanthracene, the metabolic product of the non-carcinogenic 1:2-benzanthracene, is also inactive (23rd Annual Report, *B.E.C.C.*, 1946, p. 98). Similarly, 4′:8′-dihydroxy-1:2:5:6-dibenzanthracene, the metabolite of dibenzanthracene in mice and rats, is not carcinogenic. In rabbits 1:2:5:6 dibenzanthracene is metabolized to a dihydroxy dibenzanthracene of unknown orientation, and this product is also inactive as a carcinogen (Boyland, Levi, Mawson and Roe, 1941; Dobriner, Rhoads and Lavin, 1942). 8-Hydroxy-3 : 4-benzpyrene, one of the metabolites of benzpyrene, is only very slightly carcinogenic (21st Annual Report, *B.E.C.C.*, 1944, p. 56).

The above work mostly concerns the effect of substituents on carcinogenic hydrocarbons ; but the effect of various groups on non-carcinogenic compounds is perhaps even more important. Benzanthracene is perhaps the most important non-carcinogenic hydrocarbon, as it is the parent substance of so many of the more interesting carcinogens. A large number of 10- substituted derivatives of benzanthracene has been examined, for substituents can easily be introduced into this position, either by substitution reactions, or by replacement. Many of these derivatives are tabulated in Table VII. Some derivatives are carcinogenic and some are inactive. In most cases the activity is of a low order, but the interesting feature of this work is that both electron-attracting and electron-repelling substituents can convert the inactive benzanthracene into a cancer-producing derivative. 10-Methyl-, 10-amino-, 10-mercapto-, and 10-methoxy-1:2-benzanthracenes are cancer-producing, but so also are the 10-cyano- and 10-aldehyde-derivatives. In this connection it is noteworthy that 9-methyl-10-cyano-1:2-benzanthracene is a potent carcinogen, with activity of the same order as that of 9:10-dimethyl-1:2-benzanthracene (Badger, Cook, Hewett, Kennaway, Kennaway, Martin and Robinson, 1940).

TABLE VII.—10-*Substituted*-1:2-*benzanthracenes*.

| Substituent in the 10-position. | Carcinogenic activity. Skin. | References. | Subcutaneous tissue. | References. |
|---|---|---|---|---|
| $-CH_3$ | +++ | a | ++++ | b |
| $-CH_2CN$ | .. | .. | + | d |
| $-CH_2Cl$ | .. | .. | 0 | d |
| $-CH_2SH$ | .. | .. | + | d, c |
| $-CH_2OH$ | ++ | a | +++ | d, a |
| $-CH_2OCH_3$ | .. | .. | 0 | d |
| $-CH_2OCOCH_3$ | ++ | a | +++ | d, a |
| $-CH_2OCH_2CH_3$ | + | a | 0 | a |
| $-CH_2NMe_2$ | .. | .. | 0 | d |
| $-CH_2NEt_2$ | .. | .. | 0 | d |
| $-CH_2COOH$ | .. | .. | 0 | d |
| $-CH_2COOCH_3$ | .. | .. | + | d |
| $-OH$ | .. | .. | 0 | d |
| $-OCH_3$ | .. | .. | ++ | d |
| $-NO_2$ | .. | .. | 0 | d |
| $-NH_2$ | .. | .. | + | d, a |
| $-CN$ | + | e | ++ | d, a |
| $-SH$ | .. | .. | + | c |
| $-NCO$ | .. | .. | + | c |
| $-CHO$ | .. | .. | ++ | d |
| $-CHOH.CH_3$ | .. | .. | 0 | d |
| $-COCH_3$ | 0 | e | .. | .. |

a, Badger, Cock, Hewett, Kennaway, Kennaway, Martin and Robinson (1940); b, Shear (1938); c, Dunlap and Warren (1946); d, Shear, Leiter and Perrault (1940); e, Badger, Cook, Hewett, Kennaway, Kennaway and Martin (1942).

(d) *Heterocyclic and fluorene analogues.*

Following the discovery that 1:2:5:6-dibenzanthracene has moderate carcinogenic activity, it was of interest to examine certain heterocyclic analogues and related fluorene derivatives, and considerable work in this field has now been carried out. 1:2:5:6-Dibenzanthracene (XXI), 1:2:5:6-dibenzfluorene (XXII), 1:2:5:6-dibenzacridine (XXIII) and 1:2:5:6-dibenzcarbazole (XXIV) are all slightly to moderately active, while 1:2:5:6-dibenzphenazine (XXV) and *iso*-naphthathioxin (XXVI) appear to be inactive.

XXI.

XXII.

XXIII.

XXIV.

XXV.

XXVI.

Again, 1:2:7:8-dibenzanthracene (XXVII), 3:4:5:6-dibenzacridine (XXVIII; 1:2:7:8-dibenzacridine in American numbering), 1:2:7:8:dibenzfluorene (XXIX), 1:2:7:8-dibenzcarbazole (XXX), and 3:4:5:6-dibenzcarbazole (XXXI) have given tumours, while 3 : 4 : 5 : 6-dibenzfluorene appears to be inactive (Hartwell, 1941). The effect of the introduction of one or more hetero atoms in the meso positions

XXVII.

XXVIII.

XXIX.

XXX.

XXXI.

XXXII.

is therefore somewhat irregular, but many heterocyclic analogues may possess activity of a high order. 3:4:5:6-Dibenzcarbazole is particularly potent (Boyland and Brues, 1937; Andervont and Edwards, 1941). It is also of some interest that Kirby and Peacock (1946) have found *N*-methyl-3:4:5:6-dibenzcarbazole less effective than the parent substance in producing either tumours or hyperplastic liver changes in mice. 1:2-Benzcarbazole has been reported as very slightly carcinogenic by Schürch and Winterstein (1935), and this observation should clearly be confirmed by other workers. Several *N*-alkyl derivatives (methyl, ethyl, propyl) appear to be inactive (Buu-Hoï, 1946), so that *meso* substitution seems to be effective only in an aromatic hydrocarbon ring.

A few examples of derivatives with hetero atoms at positions other than the *meso* positions have been examined. 1′-Aza-3:4-benzpyrene (XXXIII) and 1′-aza-3:4-benzphenanthrene (XXXIV) appear to be completely inactive. 3-Aza-chrysene (XXXV), 5-aza-1:2-benzanthracene (XXXVI) and 4′-aza-1:2-benzanthracene (XXXVII), which are related to non-carcinogenic hydrocarbons, are also inactive (Badger, Cook, Hewett, Kennaway, Kennaway, Martin and Robinson, 1940; Shear and Leiter, 1941; Joseph, 1939). It is of some interest, however, that the latter compound, 4′-aza-1:2-benzanthracene, gave small tumour nodules

XXXIII.

1'-Aza-3:4-benzpyrene
3(N):4-Pyridinopyrene.

XXXIV.

1'-Aza-3:4-benzphenanthrene
Naphtho(1:2f)quinoline
3(N):4-Pyridinophenanthrene.

XXXV.

3-Azachrysene (British).
1-Azachrysene (American).
Naphtho(2:1f)quinoline.

XXXVII.

4'-Aza-1:2-benzanthracene
Naphtho(2:3f)quinoline
β-Anthraquinoline.

XXXVI.

5-Aza-1:2-benzanthracene
Naphtho(1:2g)quinoline.

in the kidney in 2 of 11 rats injected subcutaneously. 1:2-Benzanthracene itself appears to have some activity in rats, for it gave hepatomas in 2 of 6 rats of the Osborne and Mendel strain which were given this hydrocarbon by mouth (Sempronj and Morelli, 1939; White and Eschenbrenner, 1945).

Activity of a very high order has been found in certain sulphur compounds closely related to 9:10-dimethyl-1:2-benzanthracene. 4:9-Dimethyl-5:6-benzthiophanthrene (XXXVIII) has produced tumours in an average time of 116

XXXVIII.

days, and thus has activity of the same order as that of the parent hydrocarbon (Dunlap and Warren, 1941; Sandin and Fieser, 1940). It also proved to be highly active when administered by injection; tumours were produced in an average of 18 weeks (100 per cent response), as compared with a latent period of 14 weeks with the hydrocarbon. Hershberg and Fieser (1941) have also prepared 9:10-dimethyl-1:2-(2′:3′-thiopheno)anthracene (XXXIX) and the corresponding selenium analogue (XL), but the biological tests do not seem to have been reported (Fieser, 1944).

XXXIX.

XL.

Compounds in which the sulphur atom replaces the phenanthrene-type double bond have been prepared by a group of workers under Sir Robert Robinson (23rd Annual Report, *B.E.C.C.*, 1946, p. 107). 4:7-Dimethyl-2:3:5:6-dibenzthionaphthene (XLI) was found to be moderately active by application to the skin, but proved inactive when administered by injection. On the other hand, 4:9-dimethyl-2:3:5:6-dibenzthiophanthrene (XLII), which again possesses a phenan-

XLI.

XLII.

threne-type double bond, is a particularly potent carcinogen both to the skin and to subcutaneous tissue. Furthermore, the isomeric 4:9-dimethyl-2:3:7:8-dibenzthiophanthrene is also a potent carcinogen (24th Annual Report, *B.E.C.C.*, 1947, p. 126).

The effect of alkyl substitution in the heterocyclic series of carcinogens has not been widely studied, except in the benzacridine series, and here some interesting differences between the 1:2-benzacridines and the 3:4-benzacridines have emerged. This work is particularly difficult to follow, as so many different systems of numbering the acridine molecule are in common use. The system used here is that commonly used by British chemists, but it differs from that used in America, where the Patterson system is used. It also differs from that currently used in French papers, where most of this work has been reported. Thus 3:4-benzacridine in Britain is the same as 1:2-benzacridine in America, and this is the same as 5:6-benzacridine in the French papers.

XLIII.

1:2-Benzacridine.

XLIV.

3:4-Benzacridine.

TABLE VIII.—*Methylbenzacridines.*

3:4-Benzacridine.*

1:2-Benzacridine.

| Compound. | Carcinogenic activity. | | | |
|---|---|---|---|---|
| | Skin. | References. | Subcutaneous tissue. | References. |
| 3:4-Benzacridine | 0 | b | — | .. |
| 5-Methyl- | 0 | b | — | .. |
| 7-Methyl- | 0 | e | — | .. |
| 5:7-Dimethyl- | 0 | a | — | .. |
| 5:8-Dimethyl- | + | c, d | 0 | c |
| 5:9-Dimethyl- | 0 | c, d | + | c |
| 2′:5:9-Trimethyl- | — | .. | + | c |
| 5:7:9-Trimethyl- | +++ | c | 0 | c |
| 1:2-Benzacridine | 0 | b | — | .. |
| 5-Methyl- | +++ | c | — | .. |
| 7-Methyl- | 0 | c | — | .. |
| 5:7-Dimethyl- | ++++ | c, d | +++ | c |
| 5:8-Dimethyl- | ++++ | c, d | +++ | c |
| 5:9-Dimethyl- | +++ | c, d | +++ | c |
| 5:7:9-Trimethyl- | +++ | c, d | ++ | c |
| 5:6:7:9-Tetramethyl- | +++ | c | + | c |

* Several different systems of numbering are in common use:

3:4-Benzacridine (in Britain).

1:2-Benzacridine (in America).

5:6-Benzacridine (in France).

1:2-Benzacridine (in Britain).

3:4-Benzacridine (in America).

7:8-Benzacridine (in France).

a, Quoted by Buu Hoï (1946), but method of test not stated ; b, Quoted by Pullman (1947*a*), but method of test not stated ; c, Lacassagne, Buu-Hoï, Lecocq and Rudali (1946) ; d, Lacassagne, Rudali, Buu-Hoï and Lecocq (1945) ; e, Bachmann, Cook, Dansi, de Worms, Haslewood, Hewett and Robinson (1937).

The mono-methyl derivatives of 3:4-benzacridine are either inactive, or only slightly active, but the introduction of several methyl groups, as in 5:7:9-trimethyl-3:4-benzacridine, gives rise to more potent derivatives. On the other hand, the mono-methyl derivatives of 1:2-benzacridine are, generally speaking, moderately or markedly carcinogenic. 5:9-Dimethyl-, 5:8-dimethyl-, and 5:7-dimethyl-1:2-benzacridines are extremely potent carcinogens, approaching methylcholanthrene in activity (Lacassagne, Rudali, Buu-Hoï and Lecocq, 1945 ; Lacassagne, Buu-Hoï, Lecocq and Rudali, 1946 ; Buu-Hoï, 1946).

(e) *Hydroaromatic compounds.*

The biological activity of hydrogenated derivatives of polycyclic aromatic hydrocarbons is of special interest, partly in view of the possibility that carcinogenic hydrocarbons may be formed *in vivo* from sterols or other normal constituents of animal cells, and partly with reference to the influence of the shape and size of the molecule on carcinogenic activity (Fieser, 1944).

In connection with the first point, it was clearly of importance to test dehydronorcholene (VII), the immediate precursor of methylcholanthrene in the laboratory preparation of this carcinogen from deoxycholic acid. It proved to be inactive, however (Shear, 1938 ; Bachmann, Cook, Dansi, de Worms, Haslewood, Hewett and Robinson, 1937).

It is also important that 6:7-dihydro-20-methylcholanthrene is inactive (Shear, Leiter and Perrault, 1941), for in this hydrocarbon only the phenanthrene-type double bond has been hydrogenated. On the other hand, hydrogenation does not always give rise to inactive compounds. 1′:2′:3′:4′-Tetrahydro-4:10-ace-1:2-benzanthracene is slightly active, having produced 4 tumours in 10 mice which received the compound by injection (Shear, 1938). No tumours were produced, however, with the related 1′:2′:3′:4′-tetrahydro-10-methyl-1:2-benzanthracene ; nor with 9:10-dihydro-10-methyl-1:2-benzanthracene ; nor with 5:6:7:8-tetrahydro-10-methyl-1:2-benzanthracene (Shear, 1939).

Of the compounds related to 3:4-benzpyrene which have been tested, 1′:2′-dihydro-4′-methyl-3:4-benzpyrene proved to have about the same activity as the corresponding fully aromatic compound, but 1′:2′:3′:4′-tetrahydro-3:4-benzpyrene gave no tumours when injected into mice, although the duration of the experiment was 20 months (Shear, 1939).

### THE METABOLISM OF POLYCYCLIC AROMATIC HYDROCARBONS

So far as is known the carcinogenic and related non-carcinogenic polycyclic aromatic hydrocarbons all undergo hydroxylation or perhydroxylation in the animal body, and there does not seem to be any essential difference between the metabolism of the carcinogens as opposed to the non-carcinogens. There is some species specificity, however, in that rabbits metabolize the hydrocarbons by a process which differs slightly from that of mice and rats (Boyland and Weigert, 1947).

α-Naphtholglycuronic acid has been isolated from the urine of dogs dosed with naphthalene, and l-α-naphthylmercapturic acid from the urine of rabbits. Recently Young (1946) has isolated l-1:2-dihydroxy-1:2-dihydronaphthalene from the urine of rats dosed subcutaneously or orally with naphthalene, and following similar experiments with rabbits Booth and Boyland (1947) have obtained a dl-1:2-dihydrodiol.

Anthracene is metabolized by a similar process. The major product in the urine of both rats and rabbits is a 1:2-dihydrodiol and monoglycuronide. Rabbits appear to produce dl-1:2-dihydroxy-1:2-dihydroanthracene, and rats the corresponding l-compound (Boyland and Levi, 1935 ; 1936*a*, *b*).

Such dihydrodiols are very readily dehydrated to phenols, and it is, therefore, not necessarily significant that dihydrodiols have not been isolated following experiments with the more complex hydrocarbons. In such experiments only phenols have been isolated (or characterized by the formation of a more stable methyl ether), although there is some evidence for the formation of such dihydrodiols (Weigert and Mottram, 1946*a*, *b*).

Berenblum and Schoental (1943*b*) have found that 1:2-benzanthracene is metabolized to the 4′-hydroxy-derivative (XLV), and the potent cancer-producing substance 9:10-dimethyl-1:2-benzanthracene is probably metabolized to 4′-hydroxy-9:10-dimethyl-1:2-benzanthracene (XLVI) (22nd Annual Report, *B.E.C.C.*, 1945, p. 53). In both cases rats were used, and no experiments with rabbits have yet been reported. Chrysene is metabolized to 3-hydroxychrysene (XLVII ; 1-hydroxychrysene in American numbering) (Berenblum and Schoental, 1945). Similarly, 3:4-benzpyrene is metabolized to 8-hydroxy-3:4-benzpyrene (XLVIII), although some 10-hydroxy-3:4-benzpyrene is also formed in small quantity. In rabbits the process is similar except that more of the 10-hydroxy- derivative is formed (Berenblum and Schoental, 1946*b*).

XLV.

XLVI.

XLVII.

XLVIII.

1:2:5:6-Dibenzanthracene is oxidized to 4′:8′-dihydroxy-1:2:5:6-dibenzanthracene in rats and mice (Dobriner, Rhoads and Lavin, 1942), but to another dihydroxy derivative (of unknown orientation) in rabbits (Boyland, Levi, Mawson and Roe, 1941 ; Dobriner, Rhoads and Lavin, 1942 ; Badger, 1947).

These metabolism experiments are of considerable interest for two reasons. Firstly, as Boyland and Weigert (1947) have emphasized, the species differences in metabolism may not be unconnected with carcinogenic action. Although 1:2:5:6-dibenzanthracene readily produces tumours at the site of injection in rats and in mice, it very rarely, if ever, produces such tumours in rabbits. Secondly, it is noteworthy that in rats the polycyclic aromatic hydrocarbons all give rise to phenols in which the hydroxyl groups occupy comparable positions. The 4′-position of benzanthracene (and of its 9:10-dimethyl- derivative) is comparable with the 3- position in chrysene, and with the 8- position in 3:4-benzpyrene. In general, however, oxidizing reagents do not attack these positions (Cook and Schoental, 1947), and the biological oxidation must therefore be conditioned by additional or other factors. It must be admitted, however, that in all metabolic experiments the end-product isolated represents only a small fraction of the amount of hydrocarbon administered. The greater part of the hydrocarbon must be broken down into simple products which escape isolation.

### THE CHEMICAL REACTIVITY OF CARCINOGENS.

Many of the most potent carcinogenic hydrocarbons are extremely reactive, and take part in substitution and addition reactions with very great facility. This pronounced chemical reactivity of some, but not all the carcinogens has attracted the attention of several workers, and many attempts have been made to find correlations between the chemical properties of the molecules and their cancer-producing activity. Probably the most extensive work in this field has been carried out by L. F. Fieser and his associates, who, in a number of papers, have developed the hypothesis that carcinogenicity may be associated with some specific chemical reaction (Fieser, 1938 ; 1944).

The addition of hydrogen to polycyclic aromatic hydrocarbons and to the cancer-producing substances in particular is complex. With phenanthrene there are three pronounced stages of hydrogenation, leading to 9:10-dihydro-, to 1:2:3:4-tetrahydro-, and 1:2:3:4:5:6:7:8-octahydrophenanthrene. Similarly, with anthracene there are three stages of reduction. Mild reduction gives the 9:10-dihydro- derivative, and more vigorous treatment gives the 1:2:3:4-tetrahydro- and 1:2:3:4:5:6:7:8-octahydro- derivatives. Most of the carcinogens are derivatives of phenanthrene, and many are also derivatives of anthracene. The hydrogenation of such compounds may take a complex course, involving either the anthracene moiety, the phenanthrene moiety, or both.

The reduction of 1:2-benzanthracene has been investigated by Fieser and Hershberg (1937). With sodium and amyl alcohol a hexahydride (XLIX) was obtained ; but with hydrogen and platinum 5:6:7:8-tetrahydro-1:2-benzanthracene (L) was formed. Reduction of the cancer-producing 10-methyl-1:2-benzanthracene proceeded in a similar fashion ; sodium and amyl alcohol gave the corresponding hexahydride, and hydrogen and platinum the corresponding tetrahydride. Furthermore, treatment of 10-methyl-1:2-benzanthracene with sodium, followed by treatment with alcohol, gave the dihydride (LI) expected from an anthracene derivative. These authors also made the very interesting observation

XLIX.

L.

LI.

that the *meso* dihydride (LI) on further hydrogenation with hydrogen and platinum gave the hexahydride (corresponding to XLIX), which shows conclusively that the tetrahydrides (L) are not produced *via* the *meso* dihydrides, as might be expected.

The reduction of methylcholanthrene is again related both to the anthracene and to the phenanthrene types. Bachmann (1936) obtained the 11:14-dihydride (LII) by the action of sodium, followed by alcohol, on the parent hydrocarbon. On the other hand, Fieser and Hershberg (1938*a*) found that reduction of methylcholanthrene with sodium and amyl alcohol gave the hexahydride (LIII), and the same hexahydride was also obtained by reduction with hydrogen and platinum, together with some 6:7-dihydro-20-methylcholanthrene (LIV).

LII.

LIII.

LIV.

The *meso* positions in anthracene derivatives are always reactive, and it was therefore of some interest to determine whether carcinogenicity is in any way bound up with this reactivity. As early as 1931, however, Cook (1931) found that the carcinogenic 1:2:5:6-dibenzanthracene is less reactive than anthracene in addition reactions involving the *meso* carbon atoms. Anthracene and its derivatives undergo a Diels-Alder type of addition with maleic anhydride to form 9:10-*endo*succinic anhydride derivatives :

Bachmann and Kloetzel (1938) found that in organic solvents the reaction is reversible, and that with different anthracene derivatives the velocity of the addition varies enormously. The presence of methyl groups in the *meso* positions was found greatly to facilitate the reaction. 9-Methylanthracene reacted faster than anthracene, and 9:10-dimethylanthracene reacted faster still. Indeed, with the latter hydrocarbon the addition proceeded at room temperature. The presence of phenyl groups in the *meso* positions was found to have the opposite effect, markedly retarding the reaction. Bachmann and Kloetzel's work indicated that there is no correlation between reactivity of this nature and carcinogenic activity, for they found that methylcholanthrene reacts about as fast as benzanthracene, and 1:2:5:6-dibenzanthracene was found to react only slowly.

The photo-oxidation of carcinogenic and related compounds is another addition reaction involving the *meso* carbon atoms which has been investigated. Anthracene itself forms a photo-oxide (Dufraise and Gérard, 1935, 1936, 1937) (LV), and certain *meso* substituted anthracene and benzanthracene derivatives, and

LV.

even more especially certain naphthacene and pentacene derivatives, form photo-oxides with very great facility. Shabad (1945) has noted that when 9:10-dimethyl-1:2-benzanthracene is exposed to light and air for 2 or 3 months it showed an appreciable decrease in carcinogenic activity. Similar observations were made by Bradbury, Bachmann and Lewisohn (1941), who found that the pure hydrocarbon in acetone protected from light and air produced tumours in an average of 2·0 months, while the corresponding time for the same hydrocarbon in acetone *not* protected from light and air was found to be 4·7 months. These observations are clearly associated with photo-oxidation. Several cancer-producing hydrocarbons such as 9:10-dimethyl-1:2-benzanthracene readily form photo-oxides, but this property is not an invariable characteristic of the carcinogens. Benzpyrene, for example, gave no photo-oxide (Cook and Martin, 1940 ; Cook, Martin and Roe, 1939).

Clar (1941) has emphasized that the linear benz-homologues of anthracene, such as naphthacene, pentacene, hexacene, and heptacene, undergo all trans-annular addition reactions, such as photo-oxidation and the addition of maleic anhydride with increasing facility. Naphthacene and pentacene are known to be non-carcinogenic, and it is abundantly clear therefore that *meso* reactivity is not associated with carcinogenic activity. Additional evidence to this effect has been provided by Iball (1940 ; Fieser and Dietz, 1931), who studied the oxidation-reduction potentials of the quinones derived from carcinogenic and related non-carcinogenic hydrocarbons. The relative values of the potentials obtained under identical conditions gives a quantitative comparison of the chemical reactivity associated with the 9:10-positions of the nucleus. Iball was unable to find any correlation between the observed potential and the carcinogenic activity of the hydrocarbon.

The coupling of diazonium reagents with various hydrocarbons has been investigated by Fieser and Campbell (1938), and many of the most potent carcinogens, including 3:4-benzpyrene, methylcholanthrene and cholanthrene were found to react or couple very readily. With these compounds an intense coloration was produced in a few minutes. These observations are interesting, for this reaction was previously thought to be confined to phenols, amines, enols and, to a much smaller extent, ethylenic compounds, especially conjugated dienes. On the other hand, there is no specificity in the reaction for 10-methyl-1:2-benzanthracene, 5:10-dimethyl-1:2-benzanthracene and other potent carcinogens did not react, and several non-carcinogenic hydrocarbons reacted readily.

There have been many attempts to study the oxidation of polycyclic aromatic hydrocarbons with special reference to carcinogenesis. Most oxidizing agents react with anthracene and its derivatives to give *para* quinones. Phenanthrene and its derivatives are normally oxidized to *ortho* quinones. Compounds in which both systems are present, as in benzanthracene, normally react predominantly as derivatives of anthracene. Chromic acid, for example, oxidizes benzanthracene to 1:2-benzanthra-9:10-quinone. Although chromic acid oxidation of 1:2:5:6-dibenzanthracene gives rise to 1:2:5:6-dibenzanthra-9:10-quinone, some 1:2:5:6-dibenzanthra-3:4-quinone is also formed (Cook, 1933*a*).

The effect of special or unusual oxidizing reagents has been investigated with interesting results. Warren (1943), for example, found that certain hydrocarbons undergo aerobic oxidation in the presence of ascorbic acid, and that this oxidation is inhibited by KCN and by $H_3PO_3$, but is unaffected by hydrogen peroxide. Anthracene is oxidized to anthraquinone, and benzpyrene to a mixture of 3:4-benzpyrene-5:8-quinone and the 5:10-quinone, together with an alkali-soluble compound which could not be isolated. This reaction is of special interest, for Kennaway, Kennaway and Warren (1944) have found that benzpyrene and some other carcinogens caused an increase in the concentration of ascorbic acid in the livers of mice, while naphthalene, anthracene and phenanthrene caused no such increase.

Cook and Schoental (1947) have investigated the oxidation of polycyclic aromatic hydrocarbons with osmium tetroxide. In the presence of pyridine this reagent was found to add exclusively to adjacent carbon atoms to form complexes which, on hydrolysis, gave rise to dihydrodiols. Indeed, osmium tetroxide appears to be a "double bond" reagent in that it does not (under these conditions) attack reactive centres, but only very reactive aromatic double bonds, and ethylenic double bonds. 1:2-Benzanthracene was oxidized to the 3:4-dihydrodiol (LVI), pyrene to the 1:2-dihydrodiol (LVII) and chrysene to the 1:2-dihydrodiol (LVIII). Related carcinogenic hydrocarbons, such as 9:10-dimethyl-1:2-benz-

LVI.

LVII.

LVIII.

anthracene, methylcholanthrene, and benzpyrene also reacted to give similar diols.

In spite of the fact that this reagent attacks double bonds, it will be noted that the position of attack differs from that experienced in biological oxidation (p. 332). If benzanthracene undergoes perhydroxylation *in vivo*, as is commonly assumed in order to account for the isolation of phenolic derivatives, this biological oxidation must take place at the 3′:4′-bond, and yet osmium tetroxide attacks the 3:4-bond. Again, anthracene is oxidized *in vivo* to the 1:2-dihydrodiol, a compound which contains an ethylenic double bond. With osmium tetroxide, anthracene gives the 1:2:3:4-tetrol (Cook and Schoental, 1948).

Perbenzoic acid is another reagent which probably attacks aromatic double bonds in preference to reactive centres. Eckhardt (1940*a*) has studied the rate of oxidation of several compounds with this reagent. The oxidation products were not determined, the oxidation simply being followed iodometrically over a 7–15-day period. Certain carcinogens such as methylcholanthrene and benzpyrene reacted very rapidly, while other non-carcinogens, such as pyrene, reacted only slowly. The correlation was not complete, however, and the reaction should certainly be further investigated.

The oxidation of polycyclic aromatic hydrocarbons with lead tetra-acetate has been investigated by Fieser and Hershberg (1938*c*). Here, again, some of the carcinogens reacted with great facility, while others proved to be more resistant to oxidation. Furthermore, some non-carcinogens were attacked while some carcinogens were unaffected. 1:2-Benzanthracene was converted into the 10-acetoxy- derivative, but the carcinogenic 1:2:5:6-dibenzanthracene was not attacked. It has been suggested that this lack of reactivity in *meso* positions adjacent to an angular ring is due to steric effects, but there is some reason to believe that the

9- position in benzanthracene and the 9:10 positions in dibenzanthracene are inherently less reactive than the *meso* positions in anthracene (Iball, 1940; Pullman, 1947*a*). Other carcinogenic agents, such as 10-methyl-1:2-benzanthracene, methylcholanthrene, and benzpyrene, were readily attacked by lead tetra-acetate. Benzpyrene gave 5-acetoxy-3:4-benzpyrene (Fieser and Hershberg, 1939), a product which is analogous to that obtained from benzanthracene. 10-Methyl-1:2-benzanthracene was attacked on the methyl group to give 10-acetoxymethyl-1:2-benzanthracene, and methylcholanthrene was attacked on the methylene group directly attached to the *meso* position. The reaction, therefore, provides a useful method for probing for the active centres in a molecule, whether such centres are located in the nucleus or in a side chain.

Badger and Cook (1939) extended the reaction to the very feebly active 9:10-dimethylanthracene, and the potent carcinogen, 9:10-dimethyl-1:2-benzanthracene, and found that both hydrocarbons react very readily. The methyl groups were found to be attacked in each case, the products isolated being 9:10-di(acetoxymethyl)anthracene and 9:10-di(acetoxymethyl)-1:2-benzanthracene. High reactivity of this nature is, therefore, not associated with the cancer-producing properties of the molecule. More recently Fieser and Putman (1947) have made an extensive survey of the effect of lead tetra-acetate on hydrocarbons. They concluded that although some correlation between chemical reactivity and carcinogenic potency is apparent, this correlation is not perfect. The rate of reaction of the 10-alkyl-1:2-benzanthracenes remained practically the same, although cancer-producing activity falls off rapidly as the length of chain increases. Furthermore, the feebly active 9:10-dimethylanthracene was actually found to react faster than the potent carcinogen 9:10-dimethyl-1:2-benzanthracene. And finally, the moderately carcinogenic 1:2:5:6-dibenzanthracene does not react with lead tetra-acetate.

Further evidence as to the reactivity of *meso* methyl groups was provided by Badger and Cook (1939), who brominated 9:10-dimethyl-1:2-benzanthracene. Both methyl groups were attacked, the product being 9:10-bisbromomethyl-1:2-benzanthracene. Barnett and Matthews (1926) had previously observed the same sort of reactivity in 9:10-dimethylanthracene, which they brominated to 9:10-bisbromomethylanthracene. It is of some interest, therefore, that Badger and Cook (1940) found that 9-methyl-1:2-benzanthracene and 6-methyl-1:2-benzanthracene are not brominated in the methyl group, but are substituted in the nucleus to give the corresponding 10-bromo- derivatives. The same paper also records the facile preparation of 10-bromo-1:2-benzanthracene from the parent hydrocarbon. It is also noteworthy in this connection that *meso*-acetyl-1:2-benzanthracene is converted into *meso*-trichloroacetyl-1:2-benzanthracene by treatment with sodium hypochlorite (Dansi and Ferri, 1939).

Many other substitution reactions have been investigated. Benzanthracene has been chloromethylated to give 10-chloromethyl-1:2-benzanthracene (Badger and Cook, 1939). 1:2-Benzanthranyl-10-mercaptan has been prepared by allowing benzanthracene to react with sulphur monochloride followed by sodium sulphide (Wood and Fieser, 1940). Benzanthracene has been nitrated, the major product being 10-nitro-1:2-benzanthracene (Barnett and Matthews, 1925; Fieser and Hershberg, 1938*c*). Benzanthracene reacts with methylformanilide to give the 10-aldehyde (Fieser and Hartwell, 1938). It has also been thiocyanated to give both 10-thiocyano-1:2-benzanthracene (57 per cent) and 9-thiocyano-1:2-

benzanthracene (5 per cent) (Wood and Fieser, 1941). Benzanthracene has also been subjected to a variety of Friedel-Crafts reactions. With ethylchloroglyoxalate, in nitrobenzene, 1:2-benzanthranyl-10-glyoxylic acid was produced in good yield (Badger and Cook, 1940). With acetyl chloride in nitrobenzene a *meso* acetyl derivative (almost certainly the 10-acetyl-) was produced (Dansi and Ferri, 1939). With acetic anhydride, however, the reaction was found to be rather more complex and the products isolated were: *meso*-acetyl-, 7-acetyl-, 6-acetyl-, and two acetyl derivatives of unknown orientation (Cook and Hewett, 1933). With oxalyl chloride both the 10-carboxylic acid and 4:10-oxalyl-1:2-benzanthracene were obtained (Dansi, 1937). Further, by heating benzanthracene and chloroacetic ester with copper at a high temperature Dansi and Ferri (1939) obtained some ethyl 1:2-benzanthranyl-10-acetate.

Relatively little work has been carried out on the direct substitution of carcinogens other than that already mentioned, and no satisfactory chemical distinction between carcinogenic and non-carcinogenic hydrocarbons has yet been discovered. 3:4-Benzpyrene, for example, is very readily thiocyanated to the 5-thiocyano- derivative; but anthracene also reacts readily, to give 9:10-dithiocyanoanthracene (Wood and Fieser, 1941), and there is clearly no specificity in the reaction. The chemical work with this reagent is interesting, however. 10-Methyl-1:2-benzanthracene was found to be substituted in the free 9- position, to give 9-thiocyano-10-methyl-1:2-benzanthracene; and 9-methyl-1:2-benzanthracene was found to be substituted in the free 10- position to give the 10-thiocyano- derivative. On the other hand, methylcholanthrene was attacked in the methylene group directly attached to the *meso* position. It is useful to compare the actions of lead tetra-acetate, and of bromine, on these hydrocarbons so far as the work has been carried out.

A few interesting substitution reactions have been carried out with benzpyrene. It is readily chlorinated in the 5- position (Windaus and Raichle, 1939), and it is nitrated to 5-nitro-3:4-benzpyrene (Windaus and Rennhak, 1937; Fieser and Hershberg, 1939; Eckhardt, 1940*b*). Windaus and Rennhak have also described a number of other substitution products, including a tribromo-derivative and a sulphonic acid. It is interesting that acetylation of benzpyrene gives mainly the 10-acetyl-derivative (Windaus and Raichle, 1939; Fieser and Hershberg, 1939). Benzpyrene also reacts readily with methylformanilide to give the 5-aldehyde (Fieser and Hershberg, 1938*c*). This reagent reacts more readily with anthracene than with benzanthracene, and the carcinogenic 1:2:5:6-dibenzanthracene is not attacked even under considerably more drastic conditions.

### THE INFLUENCE OF THE SHAPE AND SIZE OF THE MOLECULE.

It has often been suggested that carcinogenic activity is associated with certain optimum molecular dimensions, and that the shape and size of the molecule is of paramount importance for the development of carcinogenic activity. As early as 1935, for example, Barry, Cook, Haslewood, Hewett, Hieger and Kennaway remarked that their results were "in keeping with the view that there is an optimum state of molecular complexity for carcinogenic activity." The hypothesis was not, at first, described at length, but it was clearly the basis for much of the synthetic work.

Much attention has been paid to the "simplification" of the molecules of known carcinogens by the replacement of benzene rings with 5-membered rings or by one or more alkyl groups. This process led, for example, from 1:2:5:6-dibenzanthracene to 5:6-*cyclo*penteno-1:2-benzanthracene, and to 5:6-dimethyl-1:2-benzanthracene, to 5-methyl- and to 6-methyl-1:2-benzanthracene. All these compounds were found to be moderately active. In the same way cholanthrene and methylcholanthrene have been " simplified." 5:10-Dimethyl-1:2-benzanthracene (LX) is an extremely active carcinogen, as is cholanthrene (LIX). 1:2-*cyclo*Penteno-5:10-aceanthrene (LXI) is slightly to moderately active. Further " simplification " to 1:2-dimethyl-5:10-aceanthrene (LXII) to 1:2-*cyclo*penteno-5-methylanthracene (LXIII), and to 5:10-aceanthrene (LXIV), however, gives rise to inactive compounds (Shear, Leiter and Perrault, 1941 ; Shear, 1938).

LIX.

LX.

LXI.

LXII.

LXIII.

LXIV.

Many examples of this type of " simplification " could be quoted, and it was this which led Hewett (1940) to point out that nearly all the carcinogens are derivatives of phenanthrene substituted in two or more of the positions 1, 2, 3 and 4. Further substitution of 2:3-benzphenanthrene (i.e. 1:2-benzanthracene), for example, in either or both of the remaining 1- and 4- positions gives cholanthrene, 3:4-benzpyrene, and 9:10-dimethyl-1:2-benzanthracene, all of which are strongly carcinogenic. Again, further substitution of 3:4-benzphenanthrene in the remaining 1- or 2- positions also leads to pronounced activity, as in 1:2:3:4-dibenzphenanthrene, 1-methyl-3:4-benzphenanthrene, and 2-methyl-3:4-benzphenanthrene. Again, further substitution of 1:2-benzphenanthrene (i.e. chrysene) in either or both of the remaining 3- or 4- positions also leads to carcinogens, notably 1:2-dimethylchrysene. It is significant that 1:2:3:4-tetramethylphenanthrene is also slightly carcinogenic.

The hypothesis that the shape and size of the molecule is of paramount importance was stated at length by Bergmann (1942). He suggested (i) that the molecule of a carcinogen acts as a whole, and that the shape and size of the molecule determine its activity ; (ii) that all carcinogens may be conceived as parts of an " ideal " carcinogenic structure ; and (iii) that every substance which imitates more or less closely a given parent structure resembles it also in its cancer-producing action. In what amounts to an extension of the hypothesis, Lettré (1944) pointed out that when the polycyclic aromatic hydrocarbons are considered as derivatives of anthracene and phenanthrene, those which still possess a plane of symmetry through the centre of the middle ring of the two original hydrocarbons are never carcinogenic. According to this author the loss of this symmetry by substitution is a necessary but not sufficient condition for carcinogenic activity.

This hypothesis that carcinogenic activity is determined by optimum molecular dimensions is of considerable interest, and it has several advantages over any other hypothesis which has so far been announced. On the other hand, it is by no means completely satisfactory. The heterocyclic analogues and the fluorene analogues of the carcinogenic hydrocarbons provide considerable support for the hypothesis, but also many exceptions. It is of considerable interest that the methyl derivatives of 1:2-benzacridine (3:4-benzacridine in American numbering, and 7:8-benzacridine in the French papers) are very active carcinogens, as might be predicted by analogy with the corresponding benzanthracenes. On the other hand, the corresponding 3:4-benzacridines (1:2-benzacridines in American numbering, and 5:6-benzacridines in the French papers) are either inactive or have only slight activity. Again, 4:9-dimethyl-5:6-benzthiophanthrene (XXXVIII) has almost the same activity as the corresponding hydrocarbon, 9:10-dimethyl-1:2-benzanthracene, either when tested by painting on the skin or by subcutaneous injection. Yet 4:7-dimethyl-2:3:5:6-dibenzthionaphthene (XLI) is only moderately active to the skin of mice, and inactive when administered by injection.

Often quoted in support of the hypothesis that molecular architecture is of predominant importance in regard to carcinogenic activity is the observation of Dodds, Lawson and Williams (1941) that $\alpha$-ethyl-$\beta$-*sec*-butylstilbene has produced cancers in mice. This hydrocarbon (LXV) is, of course, an " open model " of 3:4-benzpyrene. Too much weight should not be given to this isolated example,

LXV.

however, for ethyl*sec*butylstilbene produced only 2 tumours in 100 mice, and in view of this very low order of carcinogenicity and to the known impossibility of avoiding occasional contamination (Earle, 1943 ; Hieger, 1946 ; Anderson, 1947), the significance of the observation must remain in some question. Benzanthracene itself has produced 1 tumour in 80 mice, and this hydrocarbon is almost invariably quoted as being non-carcinogenic.

In spite of much evidence which tends to support the view that the shape and size of the molecule governs the activity, it is difficult to believe that molecular architecture *alone* is the governing factor. It is noteworthy that activity is to be found in compounds as simple as 9:10-dimethylanthracene and 1:2:3:4-tetramethylphenanthrene and as large and complex as 1:2:3:4-dibenzpyrene and 3:4:8:9-dibenzpyrene, while many compounds of intermediate complexity are completely devoid of activity.

Some interesting experiments by Lacassagne and his co-workers may have some significance in this connection (Lacassagne, Buu-Hoï and Cagniant, 1944 ; Lacassagne, Buu-Hoï and Rudali, 1945 ; Lacassagne, Buu-Hoï, Daudel and Rudali, 1944). These workers made repeated application to the skin of mice with solutions containing a mixture of two hydrocarbons of similar molecular configuration, but one of which is weakly carcinogenic (or inactive) and the other strongly carcinogenic. The pairs of compounds were: 1:2:5:6-dibenzacridine and 1:2:5:6-dibenzanthracene; chrysene and methylcholanthrene; 1:2:5:6-dibenzfluorene and methylcholanthrene. Tumours were produced more slowly in each case than when the potent hydrocarbon alone, at the same concentration, was applied. Lacassagne supposes that the two substances, having the same affinity because of their analogy of structure, penetrate into the same cells, where they are fixed in the same substrate. Each molecule of the weakly active substance, by fixing itself in the cell, hinders the fixation of the molecule of potent carcinogen, and thus delays the appearance of tumours. The interesting feature of this work is that although the weakly active substances can hinder the rapid production of tumours, presumably by "blocking" the "receptors," they still lack the total requirements for rapid carcinogenic activity. It seems likely, therefore, that some feature in addition to the optimum molecular dimensions is necessary for the development of potent carcinogenic activity.

### THE IMPORTANCE OF THE K POSITION.

It has long been recognized that most of the carcinogens of the polycyclic aromatic type are derivatives of phenanthrene, although all derivatives of phenanthrene are not carcinogenic. In an attempt to determine whether the phenanthrene ring system is essential, 9:10-dimethylanthracene and 1:2:3:4-tetramethyl phenanthrene were submitted to extensive tests (Badger, Cook, Hewett, Kennaway, Kennaway and Martin, 1942; Kennaway, Kennaway and Warren, 1942). Both compounds are closely related to the potent carcinogen 9:10-dimethyl-1:2-benzanthracene. Both compounds were found to be slightly active, and it can only be concluded therefore that while phenanthrene is a common component of carcinogens of this type it is not absolutely essential.

In spite of this result, evidence has continued to accumulate that the phenanthrene ring system is of special significance. Robinson (1946) has suggested that an activated phenanthrene-type bridge is implicated in most carinogens, and there is considerable evidence in favour of this hypothesis. The hydrogenation of the phenanthrene-type bridge in methylcholanthrene, for example, gives 6:7-dihydro-20-methylcholanthrene, which is devoid of activity; although it must be admitted that this result is inconclusive in view of the somewhat uncertain effect of hydrogenation on carcinogenic activity (p. 331). The results obtained with certain sulphur analogues of 9:10-dimethyl-1:2-benzanthracene (p. 329) are also inconclusive, but also provide some support for the hypothesis. Again, it is of considerable interest that although 1:2:3:4-dibenzphenanthrene is a potent carcinogen, the 9- and 10- methyl derivatives of this compound are inactive (Badger, Cook, Hewett, Kennaway, Kennaway, Martin and Robinson, 1940; Harris and Bradsher, 1946). This suggests that the phenanthrene double bond must be unsubstituted, and that the presence of the methyl group offers some sort of "interference." It is, perhaps, unfortunate that these results have not been confirmed by the skin-painting technique as well as by subcutaneous injection. In any case, a similar relationship does not seem to hold with the 3- and 4-methyl-1:2-benzanthracenes, which are both moderately active by injection, although only feebly so by application to the skin. Furthermore, 7-methyl-1:2:3:4-

dibenzpyrene, like the parent hydrocarbon, is a potent carcinogen, and the phenanthrene double bond is again substituted.

In a further approach to the problem, Pullman (1947*a*, *b*, *c*, *d*), and Pullman and Pullman (1946*a*, *b*), have developed a theory relating carcinogenic activity with an optimum density of $\pi$ electrons on the phenanthrene-type double bond, or the K position as it has been called. A method for the calculation of such electronic charges has been worked out. This method is based on the resonance theory by which benzene is considered to be a *hybrid* of the structures :

A. B. C. D. E.

Summing the electronic charges required for each of these structures and calculating that the Dewar forms (C, D and E) contribute 22 per cent to the benzene hybrid and the Kekulé forms (A and B) the remaining 78 per cent, Pullman was able to draw a molecular diagram showing the distribution of the $\pi$ electrons. The calculations for tetracyclic molecules are complex, but many of these structures have now been worked out. Methyl groups act as slight donors of electrons to a ring system, and it is the essence of the Pullman theory that this increase in electronic charge converts an inactive structure (such as benzanthracene) into a carcinogen. Pullman has calculated the electronic charge of the K position for many methyl derivatives of benzanthracene, of benzphenanthrene, of 1:2-benzacridine and of 3:4-benzacridine (Table IX). The effect of a methyl group on the K position was found to vary considerably, depending on the position of the methyl group. The *meso* positions in benzanthracene, for example, were found to have the most pronounced effect, and the effect of more than one methyl group is additive. The critical charge on the double bond, below which the compounds are not carcinogenic, was found to be about 1·29*e*. At first sight the correlation between the electronic charge and the carcinogenic activity is extremely good—in fact rather *too* good, considering the uncertain nature of the approximations required in the theoretical treatment. When set out as in Table IX, however, where all the compounds have been arranged in order of increasing charge, together with assessments of activity towards skin of mice and to subcutaneous tissue, certain irregularities become apparent ; the benzphenanthrenes are especially evident, for example. Furthermore, the evidence for the *upper* limit to the optimum electronic charge is rather slender.

Clar (1941) has examined the absorption spectra of many polycyclic aromatic hydrocarbons, and has deduced interesting relationships between structure, reactivity and the wave lengths of the absorption bands. He associates certain bands with the " Dewar " forms of the hydrocarbons, and other bands with the " Kekulé " forms. Some evidence is presented that the shift in wave length (towards longer wave length) by the linear or angular addition of benzene rings, etc., can be correlated with the colour of the hydrocarbon and its " reactivity." If this correlation is valid it should be possible to compare the wave length of the appropriate absorption band with the electronic charge as obtained by Pullman, and with the carcinogenic activity. In benzanthracene, for example, Clar associates the absorption bands of the Kekulé form with the bond which

Pullman calls the K position. It is of interest, therefore, that Jones (1940, 1943) has found a limited correlation between the wave length of this absorption band and the carcinogenic activity. In the limited series of the methylbenzanthracenes the correlation is quite good ; but the correlation cannot be extended to other hydrocarbons, and, indeed, breaks down with the alkylbenzanthracenes other than the methyl derivatives. In this connection it may be mentioned that there is no correlation to be found between the wave length of the fluorescence bands and carcinogenic activity. Nor is there any correlation between carcinogenic activity and the intensity of fluorescence (Bruce, 1941 ; Bruce and Todd, 1939 ; Hieger, 1930 ; Berenblum and Schoental, 1946a).

In view of the very extensive calculations which would be required Pullman did not calculate the electronic charge for representative pentacyclic hydrocarbons ; but on general grounds these compounds were considered to support the theory. It is not difficult, for example, to understand—on the basis of this theory—why 1:2:3:4-dibenzanthracene is not carcinogenic, why 1:2:7:8-dibenzanthracene is only very feebly carcinogenic, and 1:2:5:6-dibenzanthracene is moderately active. Furthermore, the theory also provides a reasonable explanation for the relatively feeble activity of 9:10-dimethyl-1:2:5:6-dibenzanthracene ; it is only necessary to assume that the electronic charge is *above* the optimum by virtue of the two *meso* methyl groups in addition to the benz- rings. On the other hand, 9-methyl-1:2:5:6-dibenzanthracene must have a somewhat lower charge (nearer the optimum) and is strongly carcinogenic. Similarly, it is also understandable why 9:10-dimethyl-1:2:7:8-dibenzanthracene is strongly carcinogenic and 9:10-dimethyl-1:2:3:4-dibenzanthracene is inactive. Again, the effect of the *meso* nitrogen atom, as in 1:2:5:6-dibenzacridine, may be to reduce the charge on the K position and hence the carcinogenic activity. The effect of two nitrogen atoms, as in 1:2:5:6-dibenzphenazine, will be greater, and this compound is inactive.

In a further paper Pullman (1947*c*) has attempted to explain the observation that while 5:6-*cyclo*penteno-1:2-benzanthracene is a moderately active carcinogen, 6:7-*cyclo*penteno-1:2-benzanthracene is only feebly active. It was suggested that by virtue of the Mills-Nixon effect the two structures (LXVI) and (LXVII) predominate for these compounds, owing to the " bond fixation " imposed by the

LXVI.

LXVII.

five membered rings. For this reason Pullman suggests that the charge on the K region is very much greater in the case of the 5:6 compound than in the 6:7 derivative. On the other hand, it must be remembered that 5:6-dimethyl-1:2-benzanthracene is potent, and that 6:7-dimethyl-1:2-benzanthracene is only very slightly active (Barry, Cook, Haslewood, Hewett, Hieger and Kennaway, 1935). Furthermore, 1:2:5:6-dibenzanthracene is a moderate carcinogen, while 1:2:6:7-dibenzanthracene is inactive (Barry *et al.*, 1935). It is therefore possible that the effect is due as much to the position of substitution as to any " bond fixation."

In any case, the 6:7 derivative has many other structures which allow for the Mills-Nixon effect than that represented in (LXVII).

A further examination of the Pullman theory reveals many important exceptions. One would expect the methylbenzanthracenes with methyl groups in the angular ring to be active, yet all these compounds are inactive or have only trace activity. The inactive compounds of this type (all of which must be considered as exceptions) include 1′-methyl-, 2′-methyl-, 3′-methyl-, 4′-methyl-, 1′:10-dimethyl-, 2′:6-dimethyl-, 2′:7-dimethyl-, 3′:6-dimethyl- and 3′:7-dimethyl-1:2-benzanthracenes. It is also surprising that acenaphthanthracene is only slightly active, and that 3:9-dimethyl- and 5:8-dimethyl-1:2-benzanthracenes are inactive and that 7-methyl-8:9-ace-1:2-benzanthracene is inactive.

Again, as has been detailed in p. 324, a study of 10-substituted 1:2-benzanthracenes has shown conclusively that both electron-attracting and electron-repelling groups can convert an inactive parent hydrocarbon, such as benzanthracene, into a cancer-producing derivative. It is interesting to compare the effect of a methyl group with that of the cyano group, for example. 10-Methyl-1:2-benzanthracene and 9:10-dimethyl-1:2-benzanthracene are potent carcinogens. 10-Cyano-1:2-benzanthracene is only slightly active, but 9-methyl-10-cyano-1:2-benzanthracene is a particularly potent compound.

In spite of these exceptions, the Pullman theory has much to commend it as a basis for further work. The charge of $\pi$ electrons is directly associated with "reactivity," and Badger (1948) has therefore attempted to examine the validity of the calculations by an experimental method. Osmium tetroxide is known to add to the K position of many carcinogenic and related compounds (Cook and Schoental, 1947), and a method for measuring the rate of addition has been devised (Badger, 1948 ; Badger and Reed, 1948). Using this method the rate of addition of osmium tetroxide to 18 carcinogenic and related non-carcinogenic hydrocarbons and derivatives has been studied. Most of these compounds were derivatives of benzanthracene, but other representative carcinogens were also included. Other things being equal, osmium tetroxide should add more rapidly to those compounds with the greater charge on the K position. In agreement with Pullman it was found that methyl groups, especially in "favourable" positions, have a pronounced effect on the rate of reaction, and hence, presumably, on the charge at the K position. 9:10-Dimethyl-1:2-benzanthracene and other *meso* substituted benzanthracenes were found to react very much faster than benzanthracene itself, although compounds with methyl groups substituted at positions far from the K position showed only a very slight increase in reactivity. On the other hand, osmium tetroxide was found to react rapidly with both acenaphthanthracene and with 2′:7-dimethyl-1:2-benzanthracene. Indeed, the very slightly carcinogenic acenaphthanthracene reacted at about the same rate as cholanthrene—a very potent carcinogen. Furthermore, 2′:7-dimethyl-1:2-benzanthracene, which is inactive, reacted at about the same rate as 1:2-dimethylchrysene, which is a moderately potent carcinogen. These two compounds, acenaphthanthracene and 2′:7-dimethyl-1:2-benzanthracene, are, however, also exceptions to the Pullman theory, and the agreement is therefore quite good. It was also found, however, that derivatives of 3:4-benzphenanthrene reacted very much slower than benzanthracene, which is unexpected from the figures quoted in support of the theory by Pullman. Furthermore, phenanthrene itself reacted very much slower than benzanthracene, which is not the

TABLE IX.—*Density of π Electrons at K Position and Carcinogenic Activity.*

| Compound. | Density of π electrons at K position (Pullman). e. | Carcinogenic activity. | |
|---|---|---|---|
| | | Skin. | Subcutaneous tissue. |
| Naphthacene | 1·258 | 0 | — |
| Anthracene | 1·259 | 0 | 0 |
| Triphenylene | 1·260 | 0 | — |
| 3:4-Benzacridine | 1·260 | 0 | — |
| 1:2-Benzacridine | 1·270 | 0 | — |
| Chrysene | 1·272 | 0 | 0 |
| 5-Methyl-3:4-benzacridine | 1·273 | 0 | — |
| Naphthalene | 1·274 | 0 | — |
| 1:2-Benzanthracene | 1·283 | 0 | 0 |
| 5:8-Dimethyl-3:4-benzacridine | 1·284 | + | 0 |
| 5:7-Dimethyl-3:4-benzacridine | 1·285 | 0 | — |
| 5:9-Dimethyl-3:4-benzacridine | 1·286 | 0 | + |
| Phenanthrene | 1·291 | 0 | — |
| 8-Methyl-1:2-benzanthracene | 1·292 | + | 0 |
| 5-Methyl-1:2-benzacridine | 1·293 | +++ | — |
| 3:4-Benzphenanthrene | 1·293 (1·293) | + | 0 |
| 7-Methyl-1:2-benzanthracene | 1·294 | + | + |
| 6-Methyl-1:2-benzanthracene | 1·294 | + | — |
| 9-Methyl-1:2-benzanthracene | 1·296 | ++ | +++ |
| 5-Methyl-1:2-benzanthracene | 1·296 | ++ | ++ |
| 3-Methyl-1:2-benzanthracene | 1·298 | + | ++ |
| 4-Methyl-1:2-benzanthracene | 1·298 | + | ++ |
| 5:7:9-Trimethyl-3:4-benzacridine | 1·298 | +++ | 0 |
| 5:9-Dimethyl-1:2-benzacridine | 1·302 | +++ | +++ |
| 5:8-Dimethyl-1:2-benzacridine | 1·304 | ++++ | +++ |
| 5:7-Dimethyl-1:2-benzacridine | 1·304 | ++++ | +++ |
| 10-Methyl-1:2-benzanthracene | 1·306 | +++ | ++++ |
| 5:6-Dimethyl-1:2-benzanthracene | 1·307 | +++ | — |
| 5:9-Dimethyl-1:2-benzanthracene | 1·309 | — | ++++ |
| 8-Methyl-3:4-benzphenanthrene | 1·309 (1·305) | + | 0 |
| 6-Methyl-3:4-benzphenanthrene | 1·310 (1·302) | + | + |
| 4:9-Dimethyl-1:2-benzanthracene | 1·311 | — | +++ |
| 1-Methyl-3:4-benzphenanthrene | 1·312 (1·308) | ++ | 0 |
| 2-Methyl-3:4-benzphenanthrene | 1·312 (1·308) | +++ | + |
| 5:7:9-Trimethyl-1:2-benzacridine | 1·312 | +++ | ++ |
| 7-Methyl-3:4-benzphenanthrene | 1·313 (1·304) | + | 0 |
| 5:10-Dimethyl-1:2-benzanthracene | 1·317 | — | ++++ |
| 9:10-Dimethyl-1:2-benzanthracene | 1·319 | ++++ | +++ |
| 4:10-Dimethyl-1:2-benzanthracene | 1·321 | — | ++ |
| 6:9:10-Trimethyl-benzanthracene | 1·330 | ++++ | ++ |
| 5:9:10-Trimethyl-benzanthracene | 1·332 | ++++ | +++ |
| 5:6:9:10-Tetramethylbenzanthracene | 1·343 | +++ | + |

result expected from the Pullman calculations. Recently, however, Berthier, Coulson, Greenwood and Pullman (1948) have calculated the bond orders for the tetracyclic aromatic hydrocarbons by the method of molecular orbitals. These calculations are in better agreement with the kinetic experiments with osmium tetroxide in that 3:4-benzphenanthrene is given a lower bond order than benzanthracene ; but this is an observation which casts further doubt on the validity of the correlation as observed by Pullman.

It is also noteworthy that Badger found that 10-cyano-1:2-benzanthracene reacted very much slower than benzanthracene, and that the potent carcinogen 9-methyl-10-cyano-1:2-benzanthracene reacted slightly slower than the inactive benzanthracene. This is in agreement with general electronic theory, but not with the Pullman theory relating carcinogenic activity with the electronic charge on the K position.

It is of some interest to compare these results with those of Eckhardt (1940*a*), who examined the rate of oxidation of several polycyclic derivatives with perbenzoic acid. The site of oxidation is not known, but it is probably the K position. Eckhardt observed an increase in the rate of reaction following the introduction of a methyl group, and a decrease following the introduction of deactivating groups, such as —CHO and —$NO_2$. Here again, however, no complete correlation between reactivity and carcinogenic activity was observed.

It can only be concluded, therefore, that no single theory yet advanced satisfactorily accounts for the carcinogenic activity of all the polycyclic aromatic hydrocarbons and their derivatives. The hypothesis that the shape and size of the molecule governs the activity seems partly true, but will not account for many observations. Similarly, the Pullman theory, while accounting almost quantitatively for the effect of methyl groups on inactive parent hydrocarbons, fails to account for the many exceptions. It is possible that there is an element of truth in both theories, and this may be supported by the fact that the exceptions to the one theory seem to be moderately well " explained " by the other. It may be that the carcinogenic hydrocarbons interact with some tissue component to form a " complex," and that the ease of formation and the stability of this complex is governed by (*a*) the molecular dimensions of the molecule, *and* (*b*) the charge of $\pi$ electrons at one or more of the double bonds, particularly the K position. The shape and size of the molecule can be conceived as assisting in the formation of the complex by providing the best " fit " with the specific cellular receptors. In view of the pronounced aromatic character of all the carcinogens of the polycyclic type, it seems likely that the forces which bind the hydrocarbon to the tissue component are the same as those which bind these substances to polynitro compounds, to antimony pentachloride, stannic chloride, etc. It must not be expected, however, that there is any correlation between carcinogenic activity and ability (or even enhanced ability) to form complexes with such simple components. It must be admitted, however, that the introduction of a methyl group enhances the stability of the resulting picrate, and also deepens its colour. Furthermore, deactivating groups depress the stability of picrates, and such complexes as are formed are mostly lighter in colour. Most of the potent carcinogens form very dark red or purple picrates, a circumstance which is clearly related to their aromatic character, but which is not necessarily correlated with ability to produce cancers. Unfortunately, very little is known of these forces (Weiss, 1942). In any case, the complete elucidation of the problem of the relationship between chemical structure and carcinogenic activity must be left to further investigation.

SUMMARY.

1. Certain tricyclic, tetracyclic, pentacyclic and hexacyclic aromatic hydrocarbons or their homologues are cancer-producing. Most of the active compounds, but not all, are derivatives of phenanthrene.

2. Many closely related heterocyclic compounds are also cancer-producing. So are certain dibenzfluorenes.

3. The introduction of methyl groups into benzanthracene, benzphenanthrene and chrysene normally leads to enhanced activity. Two methyl groups, or more, normally act additively. On the other hand, there seems to be a limit beyond which the introduction of further methyl groups does not lead to enhanced activity. The introduction of methyl groups into the angular ring of benzanthracene does not have this effect, and all such compounds are either inactive or have only trace activity.

4. Similar relationships hold for the benzacridines, except that the 1:2-benzacridines are potentially much more carcinogenic than the 3:4-benzacridines.

5. The introduction of methyl groups into benzpyrene and dibenzanthracene is more complex. In many cases the effect is to diminish the activity, although examples of the opposite effect are also known.

6. Alkyl groups other than methyl are progressively less effective as the number of carbon atoms in the chain increases.

7. Many other substituents in " favourable " positions can convert an inactive parent hydrocarbon into a cancer-producing derivative. Both electron-attracting and electron-repelling substituents may have this property. The " favourable " positions for methyl substitution are not necessarily the same as the " favourable" positions for other substituents.

8. Partial hydrogenation of a carcinogenic compound sometimes, but not always, deactivates the substance.

9. The carcinogenic substances are—so far as is known—active as such and are metabolized to inactive derivatives.

10. The carcinogenic and related non-carcinogenic hydrocarbons are metabolized by the same mechanism, namely, hydroxylation or perhydroxylation. This oxidation takes place at centres other than those normally attacked by chemical oxidizing agents.

11. There is no correlation between chemical " reactivity " and carcinogenic activity.

12. Attempts to relate carcinogenic activity (*a*) with the shape and size of the molecule, and (*b*) with the electronic charge on the phenanthrene-type double bond present in most carcinogens, have been only partly successful.

REFERENCES.

ANDERSON, W.—(1947) *Nature*, **160**, 338.
ANDERVONT, H. B., AND EDWARDS, J. E.—(1941) *J. nat. Cancer Inst.*, **2**, 139.
BACHMANN, W. E.—(1936) *J. org. Chem.*, **1**, 347.
*Idem* AND CARMACK, M.—(1941) *J. Amer. chem. Soc.*, **63**, 2494.

*Idem*, Cook, J. W., Dansi, A., de Worms, C. G. M., Haslewood, G. A. D., Hewett, C. L., and Robinson, A. M.—(1937) *Proc. Roy. Soc.*, B, **123**, 343.
*Idem* and Edgerton, R. O.—(1940) *J. Amer. chem. Soc.*, **62**, 2550.
*Idem*, Kennaway, E. L., and Kennaway, N. M.—(1938) *Yale J. Biol. Med.*, **11**, 97.
*Idem* and Kloetzel, M. C.—(1938) *J. Amer. chem. Soc.*, **60**, 481.
Badger, G. M.—(1947) *J. chem. Soc.*, p. 940.—(1948) *Ibid.*, in the press.
*Idem* and Cook, J. W.—(1939) *Ibid.*, p. 802.—(1940) *Ibid.*, p. 409.
*Idem*, Cook, J. W., Hewett, C. L., Kennaway, E. L., Kennaway, N. M., and Martin, R. H.—(1942) *Proc. Roy. Soc.*, B, **131**, 170.
*Iidem* and Robinson, A. M.—(1940) *Ibid.*, **129**, 439.
*Idem*, Elson, L. A., Haddow, A., Hewett, C. L., and Robinson, A. M.—(1942) *Ibid.*, **130**, 255.
*Idem* and Reed, R. I.—(1948) *Nature*, **161**, 238.
Barnett, E. de B., and Matthews, M. A.—(1925) *Chem. News*, **130**, 339.—(1926) *Ber. dtsch. chem. Ges.*, **59**, 1429.
Barry, G., Cook, J. W., Haslewood, G. A. D., Hewett, C. L., Hieger, I., and Kennaway, E. L.—(1935) *Proc. Roy. Soc.*, B, **117**, 318.
Berenblum, I.—(1945*a*) *Nature*, **156**, 601.—(1945*b*) *Cancer Res.*, **5**, 561.
*Idem* and Schoental, R.—(1943*a*) *Brit. J. exp. Path.*, **24**, 232.—(1943*b*) *Cancer Res.*, **3**, 686.—(1945) *Biochem J.*, **39**, Proc. lxiv.—(1946*a*) *J. chem. Soc.*, p. 1017.—(1946*b*) *Cancer Res.*, **6**, 699.—(1947) *Brit. J. Cancer*, **1**, 157.
Bergmann, F.—(1942) *Cancer Res.*, **2**, 660.
Berthier, G., Coulson, C. A., Greenwood, H. H., and Pullman, A.—(1948) *Compt. rend.*, **226**, 1906.
Bloch, B., and Dreifuss, W.—(1921) *Schweiz. med. Woch.*, **51**, 1033.
Booth, J., and Boyland, E.—(1947) *Biochem. J.*, **41**, Proc. xxix.
Boyland, E., and Brues, A. M.—(1937) *Proc. Roy. Soc.*, B, **122**, 429.
*Idem* and Levi, A. A.—(1935) *Biochem. J.*, **29**, 2678.—(1936*a*) *Ibid.*, **30**, 728.—(1936*b*) *Ibid.*, **30**, 1225.
*Iidem*, Mawson, E. H., and Roe, E.—(1941) *Ibid.*, **35**, 184.
*Idem* and Weigert, F.—(1947) *Brit. med. Bull.*, **4**, 354.
Bradbury, J. T., Bachmann, W. E., and Lewisohn, M. G.—(1941) *Cancer Res.*, **1**, 685.
Bruce, W. F.—(1941) *J. Amer. chem. Soc.*, **63**, 304.
*Idem* and Todd, F.—(1939) *Ibid.*, **61**, 157.
Bryan, W. R., and Shimkin, M. B.—(1940) *J. nat. Cancer Inst.*, **1**, 807.
Burrows, H.—(1932) *Proc. Roy. Soc.*, B, **111**, 238.
*Idem*, Hieger, I., and Kennaway, E. L.—(1932) *Amer. J. Cancer*, **16**, 57.
Buu-Hoï, Ng. Ph.—(1946) *J. chem. Soc.*, p. 792.
Clar, E.—(1929) *Ber. dtsch. chem. Ges.*, **62**, 350.—(1941) ‘Aromatische Kohlenwasserstoffe.’ Berlin (Springer).
Cook, J. W.—(1931) *J. chem. Soc.*, p. 487, 3273.—(1932*a*) *Ibid.*, p. 456.—(1932*b*) *Proc. Roy. Soc.*, B, **111**, 485.—(1933*a*) *J. chem. Soc.*, 1592.—(1933*b*) *Proc. Roy. Soc.*, B, **113**, 277.—(1939) *Ergebnisse der Vitamin- und Hormonforsch*, **2**, 213.—(1943) Royal Institute of Chemistry, Lecture.
*Idem* and Haslewood, G. A. D.—(1933) *Chem. Ind.*, **52**, 758.—(1934) *J. chem. Soc.*, p. 428.—(1935) *J. Amer. chem. Soc.*, **57**, 1380.
*Iidem*, Hewett, C. L., Hieger, I., Kennaway, E. L., and Mayneord, W. V.—(1937) *Amer. J. Cancer.*, **29**, 219.
*Idem* and Hewett, C. L.—(1933) *J. chem. Soc.*, p. 1408.
*Iidem* and Hieger, I.—(1933) *Ibid.*, p. 395.
*Idem*, Hieger, I., Kennaway, E. L., and Mayneord, W. V.—(1932) *Proc. Roy. Soc.*, B, **111**, 455.
*Idem* and Kennaway, E. L.—(1938) *Amer. J. Cancer*, **33**, 50.—(1940) *Ibid.*, **39**, 381, 521.

*Idem* AND MARTIN, R. H.—(1940) *J. chem. Soc.*, 1125.
*Iidem* AND ROE, E. M. F.—(1939) *Nature*, **143**, 1020.
*Idem* AND SCHOENTAL, R.—(1947) *J. chem. Soc.*, p. 170.—(1948) *Nature*, **161**, 237.
DANSI, A.—(1937) *Gazzetta*, **67**, 85.
*Idem* AND FERRI, C.—(1939) *Ibid.*, **69**, 195.
DOBRINER, K., RHOADS, C. P., AND LAVIN, G. I.—(1942) *Cancer Res.*, **2**, 95.
DODDS, E. C., LAWSON, W., AND WILLIAMS, P. C.—(1941) *Nature*, **148**, 142.—(1945) *Cancer Res.*, **5**, 485.
DUFRAISE, C., AND GÉRARD, M.—(1935) *Compt. rend.*, **201**, 428.—(1936) *Ibid.*, **202**, 1859.—(1937) *Bull. Soc. chim.*, **4**, 2052.
DUNLAP, C. E., AND WARREN, S.—(1941) *Cancer Res.*, **1**, 953.—(1943) *Ibid.*, **3**, 606.—(1946) *Ibid.*, **6**, 454.
EARLE, W. R.—(1943) *J. nat. Cancer Inst.*, **4**, 165.
ECKHARDT, H.-J.—(1940*a*) *Ber. dtsch. chem. Ges.*, **73**, 13.—(1940*b*) *Ibid.*, **73**, 15.
FIESER, L. F.—(1937) 'The Chemistry of Natural Products Related to Phenanthrene.' New York (Reinhold Publishing Corp.). 2nd ed.—(1938) *Amer. J. Cancer*, **34**, 37.—(1944) A.A.A.S. Research Conference on Cancer.
*Idem* AND CAMPBELL, W. P.—(1938) *J. Amer. chem. Soc.*, **60**, 1142.
*Idem* AND DIETZ, E. M.—(1929) *Ber. dtsch. chem. Ges.*, **62**, 1827.—(1931) *J. Amer. chem. Soc.*, **53**, 1128.
*Idem*, FIESER, M., HERSHBERG, E. B., NEWMAN, M. S., SELIGMAN, A. M., AND SHEAR, M. J.—(1937) *Amer. J. Cancer*, **29**, 260.
*Idem* AND HARTWELL, J. L.—(1938) *J. Amer. chem. Soc.*, **60**, 2555.
*Idem* AND HERSHBERG, E. B.—(1937) *Ibid.*, **59**, 2502.—(1938*a*) *Ibid.*, **60**, 940.—(1938*b*) *Ibid.*, **60**, 1658.—(1938*c*) *Ibid.*, **60**, 1893, 2542.—(1939) *Ibid.*, **61**, 1565.
*Idem* AND HEYMANN, H.—(1941) *Ibid.*, **63**, 2333.
*Idem* AND NOVELLO, F. C.—(1940) *Ibid.*, **62**, 1855.
*Idem* AND PUTMAN, S. T.—(1947) *Ibid.*, **69**, 1038, 1041.
*Idem* AND SELIGMAN, A. M.—(1935) *Ibid.*, **57**, 228, 942.
HADDOW, A.—(1947) *Brit. med. Bull.*, **4**, 331.
*Idem* AND KON, G. A. R.—(1947) *Ibid.*, **4**, 314.
HARRIS, P. N., AND BRADSHER, C. K.—(1946) *Cancer Res.*, **6**, 671.
HARTWELL, J. L.—(1941) 'Survey of Compounds which have been Tested for Carcinogenic Activity.' U.S. Publ. Hlth. Service, Nat. Cancer Inst., Washington.
*Idem* AND STEWART, H. L.—(1942) *J. nat. Cancer Inst.*, **3**, 277.
HERSHBERG, E. B., AND FIESER, L. F.—(1941) *J. Amer. chem. Soc.*, **63**, 2561.
HEWETT, C. L.—(1940) *J. chem. Soc.*, p. 293.
HIEGER, I.—(1930) *Biochem. J.*, **24**, 505.—(1937) *Amer. J. Cancer*, **29**, 705.—(1946) *Cancer Res.*, **6**, 660.—(1947) *Brit. med. Bull.*, **4**, 360.
IBALL, J.—(1939) *Amer. J. Cancer*, **35**, 188.—(1940) *Ibid.*, **38**, 372.
IRWIN, J. O., AND GOODMAN, N.—(1946) *J. Hyg., Camb.*, **44**, 362.
JONES, R. N.—(1940) *J. Amer. chem. Soc.*, **62**, 148.—(1941) *Ibid.*, **63**, 151.—(1943) *Chem. Rev.*, **32**, 1.
JOSEPH, L.—(1939) *Proc. Soc. exp. Biol. N.Y.*, **41**, 334.
KENNAWAY, E. L.—(1924*a*) *J. Ind. Hyg.*, **5**, 462.—(1924*b*) *Brit. med. J.*, i, 564.—(1924*c*) *J. Path. Bact.*, **27**, 233.—(1925) *Brit. med. J.*, ii, 1.—(1930) *Biochem. J.*, **24**, 497.
*Idem* AND COOK, J. W.—(1932) *Chem. Ind.*, **51**, 521.
*Idem*, KENNAWAY, N. M., AND WARREN, F. L.—(1942) *Cancer Res.*, **2**, 157.—(1944) *Ibid.*, **4**, 367.
*Idem* AND SAMPSON, B.—(1928) *J. Path. Bact.*, **31**, 609.
KIRBY, A. H. M., AND PEACOCK, P. R.—(1946) *Brit. J. exp. Path.*, **27**, 179.
KLEINENBERG, G. E.—(1938) *Arch. sci. biol.* (*U.S.S.R.*), **51**, 127.—(1939) *Ibid.*, **56**, 39, 48.—(1939) *Chem. Abstracts*, **33**, 6429.—(1940) *Ibid.*, **34**, 5155.
KRUBER, O.—(1940) *Angew. Chem.*, **53**, 69.

LACASSAGNE, A., BUU-HOÏ AND CAGNIANT, P.—(1944) *C. R. Soc. Biol., Paris*, **138**, 16.
*Idem*, BUU-HOÏ, DAUDEL, R., AND RUDALI, G.—(1944) *Ibid.*, **138**, 282.
*Idem*, BUU-HOÏ, N. P., LECOCQ, J., AND RUDALI, G.—(1946) *Bull. Ass. franç. Cancer*, **33**, 48.—(1947) *Ibid.*, **34**, 22.
*Idem*, BUU-HOÏ AND RUDALI, G.—(1945) *Brit. J. exp. Path.*, **26**, 5.
*Idem*, RUDALI, G., BUU-HOÏ, N. P., AND LECOCQ, J.—(1945) *C. R. Soc. Biol., Paris*, **139**, 955.
LAW, L. W., AND LEWISOHN, M.—(1941) *Cancer Res.*, **1**, 695.
LEA, D. E.—(1945) *Ibid.*, **5**, 633.
LETTRÉ, H.—(1944) *Z. physiol. chem.*, **280**, 28.
MAYNEORD.—(1927) Unpublished; see Hieger (1937).
NEWMAN, M. S., AND WHEATLEY, W. B.—(1948) *J. Amer. chem. Soc.*, **70**, 1913.
PULLMAN, A.—(1947*a*) *Ann. Chim.*, **2**, 5.—(1947*b*) *Bull. Ass. franç. Cancer*, **34**, 245.—(1947*c*) *Compt. rend.*, **224**, 120.—(1947*d*) *Ibid.*, **225**, 738.
*Idem* AND PULLMAN, B.—(1946*a*) *Experientia*, **2**, 364.—(1946*b*) *Rev. Sci.*, **84**, 145.
ROBINSON, R.—(1946) *Brit. med. J.*, i, 945.
SANDIN, R. B., AND FIESER, L. F.—(1940) *J. Amer. chem. Soc.*, **62**, 3098.
SCHÜRCH, O., AND WINTERSTEIN, A.—(1935) *Z. physiol. Chem.*, **236**, 79.
SEMPRONJ, A., AND MORELLI, E.—(1939) *Amer. J. Cancer*, **35**, 534.
SHABAD, L. M.—(1945) *Cancer Res.*, **5**, 405.
SHEAR, M. J.—(1936*a*) *Amer. J. Cancer*, **26**, 322.—(1936*b*) *Ibid.*, **28**, 334.—(1938) *Ibid.*, **33**, 499.—(1939) *Ibid.*, **36**, 211.
*Idem* AND LEITER, J.—(1941) *J. nat. Cancer Inst.*, **2**, 241.
*Iidem* AND PERRAULT, A.—(1940) *Ibid.*, **1**, 303.—(1941) *Ibid.*, **2**, 99.
*Idem* AND PERRAULT, A.—(1939) *Amer. J. Cancer*, **36**, 211.
SHIMKIN, M. B.—(1940) *J. nat. Cancer Inst.*, **1**, 211.
*Idem* AND ANDERVONT, H. B.—(1940) *Ibid.*, **1**, 57.
WARREN, F. L.—(1943) *Biochem. J.*, **37**, 338.
WEIGERT, F., AND MOTTRAM, J. C.—(1946*a*) *Cancer Res.*, **6**, 97.—(1946*b*) *Ibid.*, **6**, 109.
WEISS, J.—(1942) *J. chem. Soc.*, p. 245.
WHITE, F. R., AND ESCHENBRENNER, A. B.—(1945) *J, nat. Cancer Inst.*, **6**, 19.
WIELAND, H., AND DANE, E.—(1933) *Z. physiol. Chem.*, **219**, 240.
WINDAUS, A., AND RAICHLE, K.—(1939) *Annalen*, **537**, 157.
*Idem* AND RENNHAK, S.—(1937) *Z. physiol. Chem.*, **249**, 256.
WINTERSTEIN, A.—(1936) Festschrift Emil Barell.
*Idem* AND SCHÖN, K.—(1934) *Naturwiss*, **22**, 237.
*Idem*, VETTER, H., AND SCHÖN, K.—(1935) *Ber. dtsch. chem. Ges.*, **68**, 1079.
WOOD, J. L., AND FIESER, L. F.—(1940) *J. Amer. chem. Soc.*, **62**, 2674.—(1941) *Ibid.*, **63**, 2323.
YOUNG, L.—(1946) *Canad. Chem.*, **30**, 124.—(1947) *Biochem. J.*, **41**, 417.